# Tridepsides as potential bioactives: a review on their chemistry and the global distribution of their lichenic and non-lichenic natural sources

**DOI:** 10.3389/ffunb.2023.1088966

**Published:** 2023-04-19

**Authors:** Hooman Norouzi, Mohammad Sohrabi, Masoud Yousefi, Joel Boustie

**Affiliations:** ^1^ Department of Horticultural Sciences, Faculty of Agriculture, Bu-Ali Sina University, Hamedan, Iran; ^2^ Department of Biotechnology, Iranian Research Organization for Science and Technology, Tehran, Iran; ^3^ Department of Environmental Science, Faculty of Natural Resources, University of Tehran, Karaj, Iran; ^4^ Univ Rennes, Centre National de la Recherche Scientifique (CNRS), ISCR (Institut des Sciences Chimiques de Rennes) - Mixed Research Unit (MRU) 6226, Rennes, France

**Keywords:** lichenic tridepsides, non-lichenic tridepsides, biome-based distribution, global distribution, lichenochemicals, gyrophoric acid

## Abstract

Tridepsides, as fully oxidized polyketides, have been known to exist in lichens for more than a century. Recent studies have showed that these possible defensive lichenochemicals possess various biological activities. Also, a candidate biosynthetic gene cluster was recently reported for gyrophoric acid (GA), an important tridepside. The present study focused on biosynthesis, natural sources, biological activities, and bioanalytical methods of tridepside molecules. Our survey shows that, so far, lichenic tridepsides have been reported from 37 families, 111 genera, and 526 species of lichen. Because many of their species contain tridepsides, the families Parmeliaceae, Lobariaceae, and Peltigeraceae can be considered critical lichenic sources of tridepsides. Furthermore, several species of *Hypotrachyna* in Parmeliaceae family showed lichenic tridepsides, suggesting that this genus is a viable source of tridepsides. This research also explored tridepsides from non-lichenic sources, such as non-lichenized fungi, lichenicolous fungi, endophytes, parasites, and liverworts, which offer substantial potential as biotechnological sources to produce tridepsides, which are produced in small amounts in lichen thalli. Two lichenic tridepsides have also been detected in non-lichenic sources: GA and tenuiorin (TE). Additionally, no significant correlation was found between tridepside biosynthesis and geographical distribution patterns for several potentially tridepside-producing lichens. We further showed that GA is the most studied tridepside with various reported biological activities, including anticancer, wound healing, photoprotection, anti-aging, antioxidant, cardiovascular effect, DNA interaction, anti-diabetes, anti-Alzheimer’s, anti-bacterial, and antifungal. Last but not least, this study provides an overview of some bioanalytical methods used to analyze tridepsides over the past few years.

## 1 Introduction

Lichen is a self-sustaining community composed of a primary mycobiont, morphologically undifferentiated alga and/or cyanobacterium as primary (or secondary) photobiont, and an obligate microbial community (Lücking et al., 2021). The fungal partner of lichens can form long-term partnerships with various algae and/or cyanobacteria through a thallus-shaped structure, which can thrive in even the most extreme conditions (Beckett et al., 2008). Additionally, lichens produce secondary metabolites most of which contribute to their particular environmental endurance (Kosanić and Rankovic, 2015). Depsides (didepsides, tridepsides, and tetradepsides), depsidones, depsones, dibenzofurans, usnic acids, benzyl esters, diphenylethers, terpenoids, steroids, aliphatic acids, xanthones, chromones, quinones, pulvinic acid derivatives, and carotenoids are the main categories of substances synthesized readily by most lichen species (Ahmadjian and Hale, 1973; Huneck and Yoshimura, 1996; Elix and Stocker-Wörgötter, 2008).

There has been an increasing interest in studying the biological activities of various lichenochemicals in recent years. These chemicals have a broad range of biological activities, including anti-inflammatory (Joshi et al., 2019; Lee et al., 2020), anticancer (Cimmino et al., 2019; Mohammadi et al., 2020; Solárová et al., 2020), antiviral (Luzina and Salakhutdinov, 2018; Hassan et al., 2019), antimicrobial (Basile et al., 2015; Nishanth et al., 2015; Varol, 2018), expectorant (De Barros Alves et al., 2014), and antioxidant (Khadhri et al., 2019; Prokopiev and Filippova, 2019; Sahin et al., 2019). Among the therapeutic compounds in lichens are tridepsides, mainly found in the family Umbilicariaceae (Posner et al., 1992; Narui et al., 1996; Narui et al., 1998).

Crustinic acid (CA), gyrophoric acid (GA), hiascic acid (HA), ovoic acid (OA), tenuiorin (TE), umbilicaric acid (UA), lasallic acid (LA), and deliseic acid (DA) are the main tridepsides detected in various lichens so far (Huneck and Yoshimura, 1996; Narui et al., 1996; Narui et al., 1998; Podterob, 2008). GA stands out as the most prominent member of this group, but it is also the most widely reported lichenic acid found in pioneer studies. For example, Stenhouse (1849) reported GA from *Lasallia pustulata* (formerly known as *Gyrophora pustulata*), a lichen that has long been used for dyes in Norway. Orchil (archaic spelling: archil) is the blue or violet color obtained from this lichen species. As noted by Stenhouse (1849), GA is the coloring principle of *L. pustulata*. GA was initially thought to be a didepside until it was disproved in 1925, and its structure was discovered and approved as a tridepside (Canter et al., 1933). In the following years, other tridepsides were discovered in various lichen genera, including *Umbilicaria, Peltigera*, and others (Huneck and Yoshimura, 1996). As a result of their wide distribution in lichens, tridepsides do not follow species- or genus-specific distribution patterns. It might therefore be necessary to carry out a study on their trends over time.

Over the past few years, the biosynthetic gene cluster assignment of fungal secondary metabolites has gained more importance (Singh et al., 2021). It is common for clusters to contain the majority, if not all, of the genes involved in the biosynthesis of a particular secondary metabolite and its probable derivatives (Kim et al., 2021). There are few lichen secondary metabolites with their gene clusters being analyzed and reported. Orsellinic acid (Jørgensen et al., 2014), usnic acid (Abdel-Hameed et al., 2016; Wang et al., 2018), grayanic acid (Bertrand et al., 2018), lecanoric acid (Lünne et al., 2020; Kealey et al., 2021), physodic acid (Singh et al., 2021), olivetoric acid (Singh et al., 2021), and atranorin (Kim et al., 2021) are some of the lichen secondary metabolites with assigned biosynthetic gene clusters. This trend will contribute to further biotechnological activities concerned with lichenic substances. Interestingly, a biosynthetic gene cluster was recently assigned to GA. For the first time, in an elaborate study, Singh et al. (2022) unraveled a candidate gene cluster involved in the biosynthetic pathway of one of the most studied tridepsides. Although these findings have set the ground for further exciting discoveries, they have also raised the level of complexity about the biosynthesis of other tridepsides and their identification. This study will look at how this gene cluster assignment may change the way we harness analytical chemistry techniques to identify lichen substances, especially tridepsides.

Tridepsides have also been found to possess a variety of pharmacological effects. For instance, some lichen substances have been recognized as UV filters (Boehm et al., 2009), and GA is a compound of relevance in this context (Nguyen et al., 2013). In any case, since several other biological activities, including anticancer, antimicrobial, antioxidant, and anti-diabetic, have been attributed to GA or compounds of the same class (Ranković and Kosanić, 2015), these compounds have attracted increased attention. Furthermore, tridepsides are a relatively understudied group of lichenochemicals that may lead us to discover new, promising bioactive compounds with therapeutic potential (Ranković, 2019; Goga et al., 2020; Mohammadi et al., 2020). Recently, some non-lichenic tridepsides with interesting pharmacological activities have also been reported, particularly from cultivable fungal endophytes (Rivera-Chávez et al., 2013; De Medeiros et al., 2015), which could be of considerable importance from a biotechnological point of view. By harnessing these non-lichenic sources, we might produce important tridepsides that are hard to obtain from lichens because of their slow growth. Tridepsides are compounds with diverse biological activities that might inspire the development of biotechnological products. This paper provides an overview of recent trends in lichenic and non-lichenic sources of tridepsides, bioanalytical procedures to obtain them, and their biological effects. In addition, we present the global distribution of some representative tridepside-producing lichen species. Such a study could lead to an overview of tridepsides’ biochemistry and specific biological activities to pave the way for their possible medical or cosmetic applications.

## 2 Methods

We performed an in-depth study of the published literature on tridepsides between 1840 and 2021 in this study. The keywords “tridepside,” “gyrophoric acid,” “umbilicaric acid,” “tenuiorin,” “crustinic acid,” “ovoic acid,” “deliseic acid,” “hiascic acid,” lasallic acid,” “biological activity,” “lichen substances,” “secondary metabolites of lichens,” “lichen chemistry,” “depside,” “depside biosynthesis,” “depside polyketide synthase,” and “tridepside extraction” were searched in several online databases, including Google Scholar^1^, Scopus^2^, PubMed^3^, Recent Literature on Lichens^4^, the Web of Knowledge, and SciFinder^5^. In order to determine the geographical distribution, we paid particular attention to studies where tridepsides had been identified using spectrometric data or modern hyphenated techniques, such as high-performance liquid chromatography (HPLC) and high-performance thin layer chromatography hyphenated (HPTLC) with Diode Array Detector (DAD) or mass spectrometry (MS). As a result, we focused our attention on the literature published over the last decade and selected 24 representative tridepside-containing lichen species to investigate their global distribution. So, we used the spocc package (Chamberlain et al., 2021) in the R 4.0.3 environment (R, 2020) to collect the species distribution records from the following online databases: Atlas of Living Australia (ALA^6^), Integrated Digitized Biocollections (iDigBio^7^), Biodiversity Information Serving Our Nation (BISON), Global Biodiversity Information Facility (GBIF^8^), and iNaturalist^9^. Following that, a global distribution map for tridepside-containing species was created. On this basis, we calculated the occurrence percentage of each species in the following biomes: Boreal Forests Taiga, Deserts and Xeric Shrublands, Flooded Grasslands and Savannas, Mediterranean Forests Woodlands and Scrub, Montane Grasslands and Shrublands, Temperate Broadleaf and Mixed Forests, Temperate Conifer Forests, Temperate Grasslands Savannas and Shrublands, Tropical and Subtropical Coniferous Forests, Tropical and Subtropical Dry Broadleaf Forests, Tropical and Subtropical Grasslands Savannas and Shrublands, Tropical and Subtropical Moist Broadleaf Forests, and Tundra (Olson et al., 2001). A series of bar charts, including frequencies of species within different lichen families and species with specific tridepsides, were created in Excel 2019. We used GBIF’s Species Matching tool for normalizing scientific names. We created the heatmaps using Heatmap Illustrator 1.0 (Deng et al., 2014). Heatmaps were clustered using maximum linkage hierarchical clustering with Euclidean distance as a similarity metric. We also used Mycobank^10^ to check the updated scientific names of the fungi and lichenized fungi mentioned in the manuscript.

## 3 Pharmacological importance of tridepsides

A variety of lichen species are traditionally used in different cultures worldwide for their culinary and ethnomedical potential (Crawford, 2019), consistent with their potential medicinal value. Lichens have been scientifically proven to be the source of various natural products with a wide range of bioactivities and a high potential for medical applications (Ranković, 2019). In our online resources, we have provided information on the different biological activities of tridepsides (ESM-1, **Table S1**). **Table S1** highlights GA as the most studied tridepside as it is evaluated for several biological functions and has proven to be a multifunctional lichenochemical.

Multiple studies have examined the potential antiproliferative effect of GA (ESM-1, **Table S1**). In the most recent study, Mohammadi et al. (2022) showed the potent cytotoxicity of GA against breast cancer cell lines. The antiproliferative activity of this compound has also been demonstrated against several other cancer cell lines. For example, it has indicated significant dose- and time-dependent antiproliferative activity against HeLa cells (Goga et al., 2019) and A375 melanoma cancer cell line (Cardile et al., 2017). It has also been shown that GA is not toxic to human fibroblasts (Cardile et al., 2017; Goga et al., 2019). In addition, IC_50_ values of 151.65, 151.51, 64.01, and 78.45 μg/ml have been reported for GA against LS174 (colorectal cancer), A549 (lung cancer), Fem-X (skin cancer), and K562 (leukemia) human cancer cell lines (Kosanić et al., 2014). Compared to cis-DDP as a positive control, GA exerted moderate inhibition on Fem-X and K562 cancer cells (IC_50_ = 0.86 and 2.22 μg/ml, respectively). Additionally, GA had cytotoxic activity against A2780 and HL-60 cell lines with IC_50_ values of 198.3 and 146.7 μM, respectively (Bačkorová et al., 2011). Thus, GA displays potential antiproliferative activity at higher concentrations, as shown by the examples presented here. A review of the potential mechanisms of action associated with the antiproliferative activity of GA is provided in **Table S1** (ESM-1).

Furthermore, there is evidence suggesting that GA may contribute to tissue regeneration. Burlando et al. (2009) reported a significant wound closure effect on confluent HaCaT keratinocyte layers upon GA application (180 μM). Also, GA increased the number of cell migrations in a Transwell plate where 10^5^ HaCaT cells were seeded. Moreover, GA demonstrated synergistic activity in healing wounds when combined with usnic acid (2 μM). GA may also provide photoprotection since it protected HaCaT cells exposed to UV-B (2.5 J/cm2) in the presence/absence of GA and vulpinic acid (Varol et al., 2016). Alpha-tocopherol’s photoprotective activity as a positive control was significantly lower than that of GA. This compound also showed preventive activity for the aftereffects of UV-B compared to the positive control, but no apoptotic activity was observed.

A study by Varol et al. (2016) concluded that GA could reduce the intensity of absorbed UV radiation by fluorescing but would have limited cosmetic application due to its potential cytotoxicity. Nevertheless, as Lohezic-Le Devehat et al. (2013) reported, GA may act as a potential UV-A filter. The sun protection factor (SPF) for GA was 5.03, higher than Homosalate (SPF = 3.91). GA also exhibited a protection factor-UV-A (PF-UVA) of 1.77, and its photostability after exposure to UV-A and UV-B was significant. The researchers reported no phototoxicity for GA, suggesting that it may have potential use as a UV filter in cosmetics. Besides this, GA has been proven to have potent anti-aging effects. According to Shim (2020), GA prevented skin aging in normal HDF cells exposed to UV-A radiation. Even after the HDF tissue had been exposed to UV-A, GA increased the mRNA level of specific collagen-encoding genes. The second effect of GA was the increased expression of superoxide dismutase 2 (SOD2) in UV-treated fibroblasts. Accordingly, GA photoprotection ambiguity requires a closer look at the contradictory data related to the cytotoxicity of GA. Even more importantly, though, is the need for further research into the photoprotective properties of other tridepsides since they share chemical structures similar to GA.

Other potential therapeutic effects of GA include cardiovascular action as a direct angiotensin II type-1 receptor antagonist (IC_50_ of 29.76 µM) (Huo et al., 2019), DNA interaction as a potential DNA-binding compound (Plsíkova et al., 2014), and anti-diabetic activity through antiglycation and anti-urease activity (Seo et al., 2009; Choudhary et al., 2011). There is also evidence that TE, the di-methylated analog of GA, may prevent Alzheimer’s disease. An experiment by Salgado et al. (2020) showed that TE inhibited tau protein aggregation in 75 and 100 μM doses. Preventing neurodegenerative diseases relies heavily on the inhibiting effect of tau protein aggregation.

Our online resource provides **supplementary information** on the biological activities of various tridepsides, including their possible mechanism of action (ESM-1, **Table S1**). As part of the **supplemental information**, we have also included the results of several studies discussing the antimicrobial activities of GA and TE (ESM-1, **Table S2**). These biological findings suggest that we may have case of interesting bioactive substances found in lichens.

## 4 Biosynthetic pathway and chemistry

**Figure 1:**
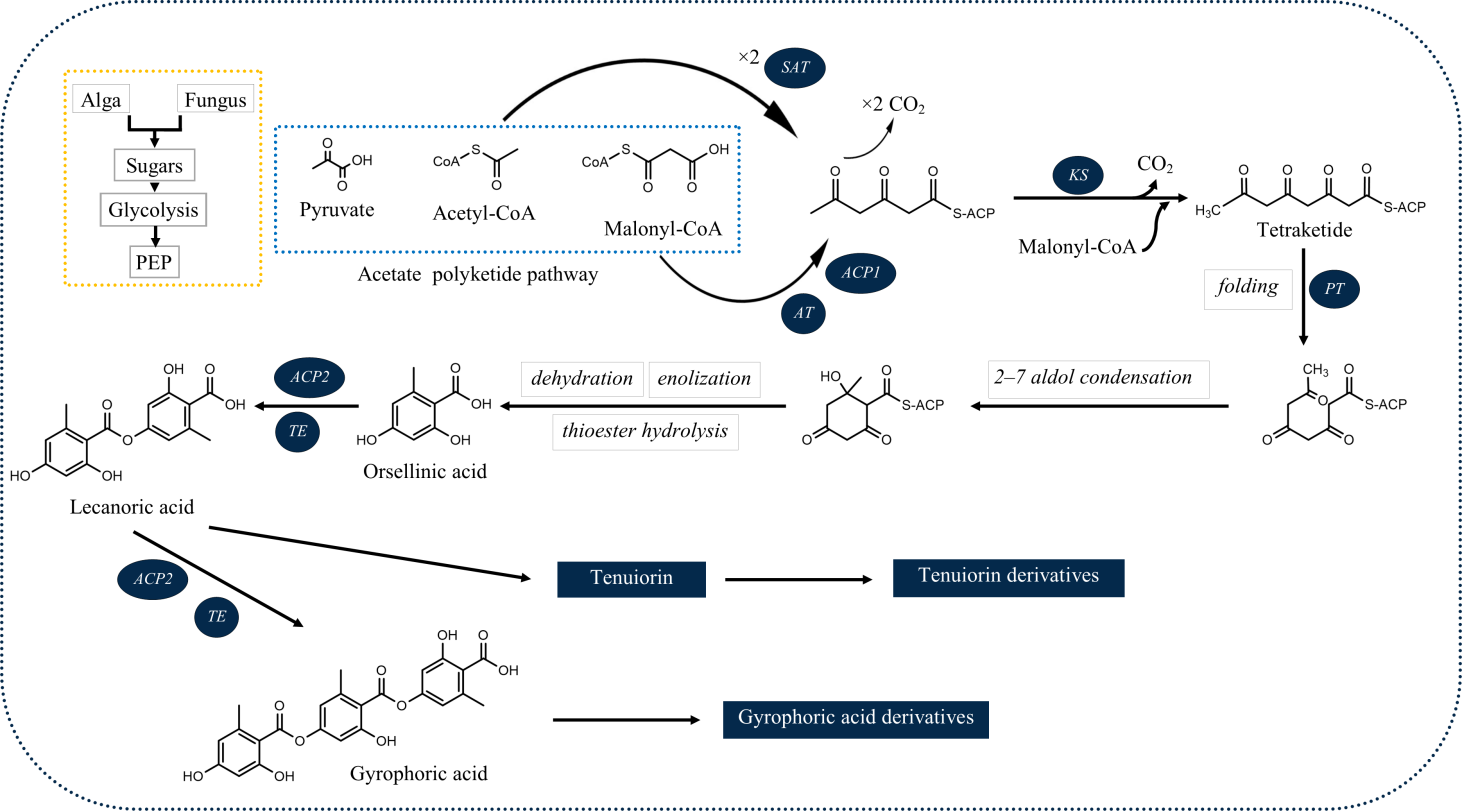
Deduced biosynthetic pathway of some of the common lichenic tridepsides. SAT, starter acetyltransferase; KS, ketosynthase; AT, acyltransferase; PT, product template domain; ACP1, acyl carrier protein 1, ACP2: acyl carrier protein 2; TE, thioesterase).

Among the products of subsequent esterification of orcinol-type phenolic units are tridepsides (Culberson and Elix, 1989). As illustrated in **Figure 1**, for tridepside biosynthesis, malonyl-CoA should be produced in the first stage. The latter, in turn, forms polyketides. A polyketide chain will be formed by intermolecular Claisen condensation of S-CoA tetraketide. During polyketide biosynthesis, polyketide synthases (PKSs) act as the primary catalytic enzymes (Talapatra and Talapatra, 2015). The domain structure of different PKSs has been revealed over the past few years. For instance, Lünne et al. (2020) identified the *PKS7* gene cluster responsible for the biosynthesis of lecanoric acid and ethyl lecanorate in *Claviceps purpurea*. This gene cluster is presumed to encode for different domains of polyketide synthase PKS7, including starter acetyltransferase (SAT), ketosynthase (KS), acyltransferase (AT), product template domain (PT), acyl carrier protein 1 (ACP1), acyl carrier protein 2 (ACP2), and thioesterase (TE). The authors also introduce the ACP2 and TE as the probable dimerization domains of PKS7. This is because of the findings of an earlier study (Jørgensen et al., 2014) in which the domains of PKS14, involved in the biosynthesis of orsellinic acid in *Fusarium graminearum*, were identified. PKS14 lacks the ACP2 and TE domains and its final product is orsellinic acid.

**Figure 2:**
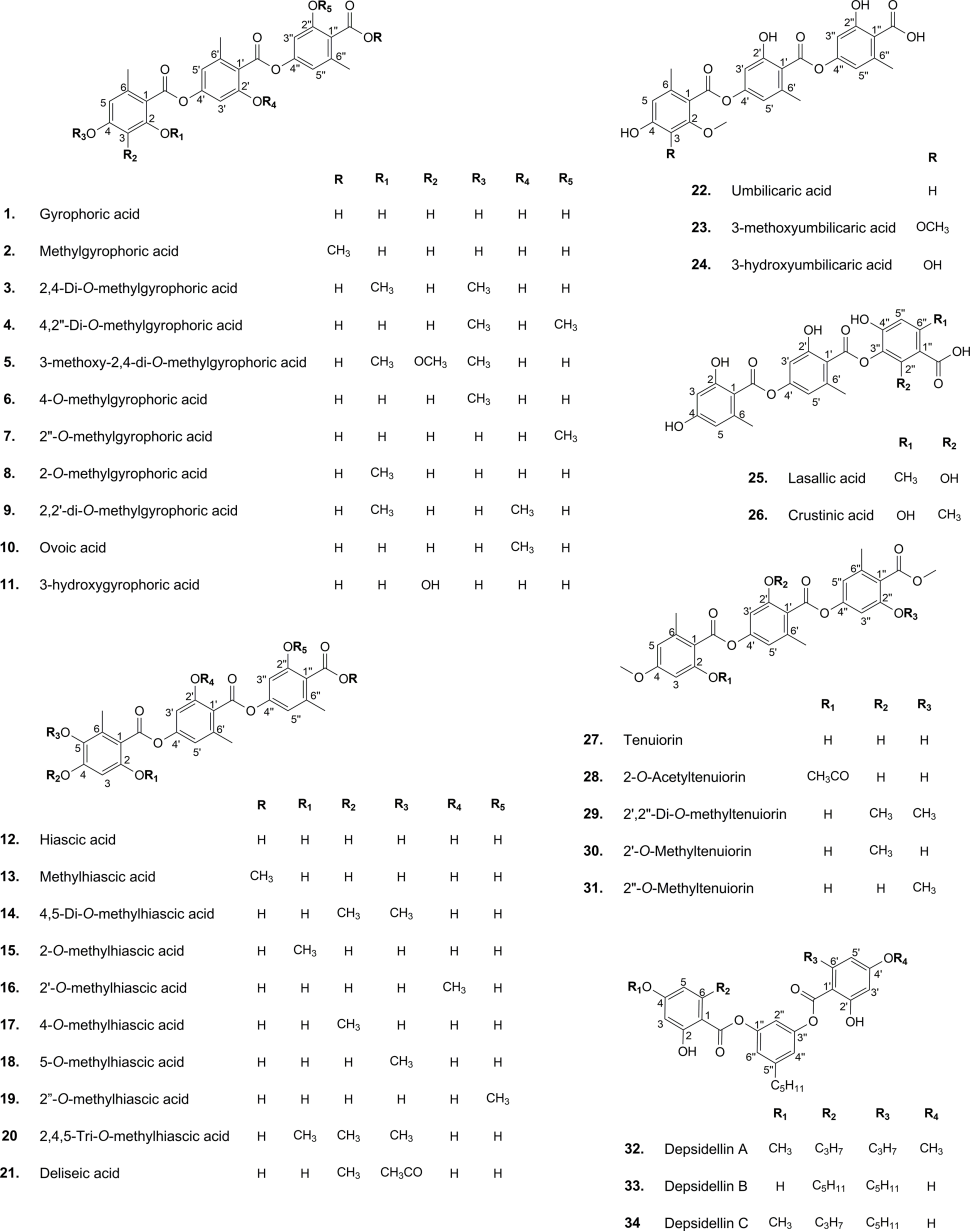
Chemical structure of common lichenic tridepsides and their derivatives.

The results of the research carried out by Singh et al. (2022) have shed some light on this. In their study, the authors have identified a gene cluster (PKS16) involved in the biosynthesis of GA in several species of *Umbilicaria* lichen genus. PKS16 comprises all previously reported domains for PKS7, including SAT, KS, AT, PT, ACP1, ACP2, and TE. They have also proposed the same PKS as the main biosynthetic gene cluster responsible for the biosynthesis of other tridepsides, such as UA and HA. However, it remains to be discovered whether the same PKS underlies the biosynthesis of GA and its derivatives in many other distant taxa reported to be containing such tridepsides (ESM-2, **Table S7**). Building on the fact that one PKS may be responsible for the biosynthesis of several tridepsides, we will face another complexity that further needs to be studied. If PKS16 codes for the synthesis of GA, the backbone compound of many tridepsides, then 24 of the tridepsides depicted in **Figure 2** are probably coded by the same gene cluster, and other genes of unknown function in this cluster are performing the other changes resulting in the synthesis of derivatives. Singh et al. (2022) have also shown that the *PKS* gene is one of the few conserved genes in the GA acid gene cluster across different *Umbilicaria* species. This may approve that all the species containing GA or one of its derivatives may have PKS16 in their genomes, which means that the *PKS* gene is active, and not silent in all these species. In this situation, we expect the backbone (GA) to be produced by *PKS* and other derivatives to be produced as a result of the coexpression of other genes in the cluster. But the problem is that in many studies that have reported the presence of tridepside derivatives, the main tridepside itself has not been detected using classical analytical methods such as TLC (ESM-2, **Table S7**). This becomes more of a problem when we consider that derivatives are primarily reported as minor compounds, which raises the question “what happens to the main building block that we cannot detect it using TLC?” So, biosynthetic gene cluster assignment may confirm that classical analytical methods are not reliable anymore because such a presence/absence pattern cannot be justified by silent genes.

The SAT domain is responsible for selecting and loading the starter unit (acetyl-CoA) in tridepside biosynthesis. Decarboxylative condensation of polyketide chains is catalyzed by the KS and AT domains. KS catalyzes the chain extension of the ACP-bound acyl chain after AT loads the malonate extender units (malonyl-CoA). The PT domain is responsible for cyclization. It is also likely to act as a protective pocket for the growing chain, which would otherwise be highly unstable and cyclized chaotically (Cox et al., 2018).

Once the polyketide chain is formed, it undergoes various modifications by which orsellinic acid will be generated as the primary building block of depsides. Following the esterification of orsellinic acid units, tridepsides of various types can be formed. This is where the ACP2 and TE domains come in. The first esterification of orsellinic acid leads to the *para*-depside lecanoric acid, which is then esterified to give the tridepside gyrophoric acid. Naturally occurring *O*-methylated derivatives of gyrophoric acid are methylgyrophoric acid, 4-*O*-methylgyrophoric acid, umbilicaric acid, ovoic acid, and 2”-*O*-methylgyrophoric acid (Elix et al., 1995). TE seems to be formed by a methylation process of GA. The structural similarity of GA and TE suggests that one or more *O*-methyltransferases may be responsible for the methylation of GA. So, the same PKS may be responsible for the biosynthesis of TE. Hydroxylation of gyrophoric acid at the C-5 position results in a distinct aromatic series represented by hiascic acid (Posner et al., 1992). Lasallic acid and crustinic acid, which are relatively similar in structure, have two orsellinic acid moieties (lecanoric acid), but their third aromatic unit distinguishes them from other tridepsides (Narui et al., 1996). Depsidellin A-C appear to possess an olivetol moiety in their structures that leads to these tridepsides after condensation with different units such as 2-hydroxy-4-methoxy-6-propylbenzoic acid, which gives depsidellin A (Elix and Wardlaw, 1997). The side chain on the C-5″ position of depsidellin A-C may indicate the intervention of other PKSs in their biosynthetic pathway. Therefore, various tridepsides might have different biosynthetic pathways, and not all of them are synthesized the same way (**Figure 1**). Such differences have led to the discovery of many tridepside derivatives in lichens (**Figure 2**) mainly related to gyrophoric acid (di-*O* substituted) and hiascic acid (tri-*O* substituted).

Taken together, the interesting steps taken in the biosynthetic gene cluster assignment of lichen secondary metabolites using genome mining approaches, together with the promising progress in the heterologous expression of depsides (Kealey et al., 2021; Kim et al., 2021) is likely to provide a novel method for the production of valuable compounds, especially tridepsides. The first step in this process is the putative identification of gene clusters, like what has happened for GA (Singh et al., 2022). Then, this putative gene cluster can be directly linked to its corresponding secondary metabolite through heterologous expression. For example, Kealey et al. (2021) successfully produced lecanoric acid by the heterologous expression of its corresponding PKS, from the lichen *Pseudevernia furfuracea*, in *Saccharomyces cerevisiae.* In a relatively similar study, Kim et al. (2021) identified a putative gene cluster for atranorin and heterologously produced it using a plant-pathogenic fungus, *Ascochyta rabiei.* So, the production of biologically active tridepsides, such as GA, is possible using the same procedure.

## 5 Extraction, isolation, and identification of tridepsides

Several advanced extraction methods have been used recently to extract lichen compounds, including accelerated solvent extraction (ASE) (Schinkovitz et al., 2014), supercritical fluid extraction (SFE) (Zizovic et al., 2012; Zugic et al., 2016; Brovko et al., 2017), microwave-assisted extraction (MAE) (Van der Wat and Forbes, 2019), and ultrasound-assisted extraction (UAE) (Khadhri et al., 2019). Mohammadi et al. (2020) presented a detailed review of lichen substances’ characterization. Their observations have shown that conventional extraction methods, namely maceration extraction (ME) and using the Soxhlet apparatus (SE), are dominant among experts, whereas only a few studies have used advanced extraction techniques. The majority of studies we investigated used ME or UAE extraction techniques to extract tridepsides (ESM-1, **Table S3**). Liquid-liquid extraction, SE, and heat reflux extraction (HRE) are the less common approaches, although they have been successfully used to extract tridepsides (ESM-1, **Table S3**). Nevertheless, ME is still the primary extraction method for most lichenic substances, which is also true for tridepsides. This trend can be seen in **Table S3** (ESM-1), where many experts prefer ME for the extraction part of their research.

A relatively advanced extraction method for tridepsides appears to be UAE (ultrasonic bath/probe). Taking advantage of waves of extreme frequency (>20 KHz), UAE can reduce the extraction period of natural substances (Belwal et al., 2018). As a result of UAE’s mechanical feature, cavitation incidence will be facilitated, which will aid in permeabilizing the solvent into the cells resulting in analyte emission in the solvent (Gallo et al., 2018). Because the extraction process can be controlled thermally, it is an efficient method for extracting thermo-labile compounds (Imam et al., 2019). Occasionally, UAE might stimulate the generation of reactive oxygen species (ROS) that could lead to the destruction of several compounds (Belwal et al., 2018). Further, UAE is a promising method for extracting intracellular substances (Gallo et al., 2018), while lichenic substances are mostly extracellular substances (Elix and Stocker-Wörgötter, 2008). It is, therefore, unlikely that UAE can be the best method of extracting lichenic substances. However, UAE can be considered a rapid, advanced extraction method that has been used in recent years for extracting tridepsides according to **Table S3** (ESM-1). Moreover, recent findings suggest that MAE using ionic liquids can provide a convenient extraction method for lichenic substances. Komaty et al. (2021) showed that using ionic liquid-based MAE (IL-MAE) improves the extraction efficiency of lichenic compounds. Although there is no study related to tridepside extraction using IL-MAE, it might be of great importance to investigate the efficiency of this method in tridepside extraction. IL-MAE not only presents a reproducible and eco-friendly method but could also be scaled up to industrial applications due to recyclable solvents, selectivity in extraction, and higher efficiency compared to conventional extraction methods and organic solvents. Overall, it is suggested that when it comes to depside extraction, it would be reasonable to choose an extraction method with mild conditions to reduce the possibility of decomposition and ester bond cleavage in tridepsides’ structure. Huneck et al. (1989) proved that the thermal decomposition of lecanoric acid and GA leads to fragments corresponding to those of mass spectrometry.

Considering purification methods, low-pressure column chromatography (LPCC) with Silica-packed columns and Sephadex^®^ LH-20 (dextran-based gel) has been a common method of pre-concentration and isolation of tridepsides, particularly GA. Furthermore, flash chromatography (FC) has also been used as an accelerated mode of LPCC for the isolation of tridepsides in several studies (see ESM-1, **Table S4**).

Preparative-thin layer chromatography (P-TLC), as another common isolation method, is an inexpensive and readily accessible technique with reliable purification results, which rapidly leads to acceptable amounts of separated compounds (1 mg/g) (Rabel and Sherma, 2017). Despite the popularity of P-TLC in the not-so-distant past, certain cutting-edge technologies, such as preparative-high performance liquid chromatography (P-HPLC), interested researchers more due to their higher accuracy and efficiency. However, despite being inexpensive and easily accessible, P-TLC is not highly effective in separating matrices with more than three major compounds. Thus, a fractionation procedure is required in advance (Gibbons, 2012). **Table S4** (ESM-1) shows that toluene and acetic acid solution is an appropriate P-TLC mobile phase for tridepsides. One of the drawbacks of the isolation of lichenic substances, especially those with acidic nature such as tridepsides, is the possible irreversible adsorption of compounds in isolation methods based on a solid-phase support. This possible process can lead to further compound decomposition. Considering this hypothesis, Oettl et al. (2014) used centrifugal partition chromatography (CPC) as a solid-free support method to isolate some lichenic substances, including six depsides, from *Pseudevernia furfuracea*, and they confirmed the reliable performance of CPC. Therefore, to avoid possible degradations of tridepsides during the isolation procedure, liquid-liquid isolation methods are suggested to be studied.

As presented in **Table S5** (ESM-1), HPLC and LC-MS are the preferred methods of identifying or quantifying tridepsides today. Nevertheless, there has been no study concerning their method validation regarding tridepside identification and quantification. Methanol and acidified water are the most common eluents for HPLC analysis of tridepsides, while acetonitrile and water seem to be the prevalent mobile phase for LC-MS analysis (ESM-1, **Table S5**). Since tridepsides are aromatic compounds, they are often detected using UV, primarily through HPLC-DAD. When mass spectrometry is used to analyze depsides, these compounds are highly fragmented, but they are still recognizable when the ionization parameters are tuned to moderate levels. When it comes to isomers that are frequently encountered in this class of compounds, the *m/z* of generated fragments can be used to distinguish them. Moreover, when trying to differentiate between these isomers, multiple parameters must be taken into account. For example, as shown in **Table S6** (ESM-1), five tridepside isomers of hiascic acid (18, 16, 17, 18, and 24) can typically be distinguished by the combination of physicochemical characteristics and chromatographic behavior. In this regard, we can take advantage of the standardized chemical tests (e.g., TLC, HPLC, and spot test) that have been introduced for lichens. Such standard tests can lead to different TLC Rf and HPLC Rt values, different colors in lichen thalli when reacting with standard reagents, and other useful features that are highly specific to each compound (Elix, 2014). NMR analysis of these compounds shows a lot of overlapped signals, and 2D techniques are particularly indicated to solve these spectra. IR and Raman spectroscopy are other possible techniques for identifying tridepsides. Several studies have successfully used this method to detect gyrophoric acid (Edwards et al., 2003a; Edwards et al., 2003b; Jorge-Villar and Edwards, 2010; Miralles et al., 2015). Due to its non-destructive nature, Raman spectroscopy could be advantageous when identifying lichenic substances. Using Raman spectroscopy, important biomarkers in the lichen thallus can be identified without removing the thallus from its substrate. This obviates, as well, the need for lengthy and, in most cases, harmful solvent-based extraction processes. As an added benefit, Raman spectroscopy offers a convenient way of monitoring lichenic substances in a thallus over a selected period. In addition, it is possible to determine the spatial distribution of lichenic substances in the thallus without destroying it (Edwards et al., 2003b).

On the whole, we can divide the identification methods into two distinct groups, namely destructive and non-destructive methods. The destructive identification methods of TLC, HPLC, and LC-MS require the lichen thallus to be grounded and extracted in organic solvents. However, it remains to be seen whether Raman spectroscopy is as practical as expected when accounting for real-time differentiation between morphologically close taxa.

## 6 Natural sources of tridepsides

### 6.1 Occurrence and ecology in lichenic sources

**Figure 3:**
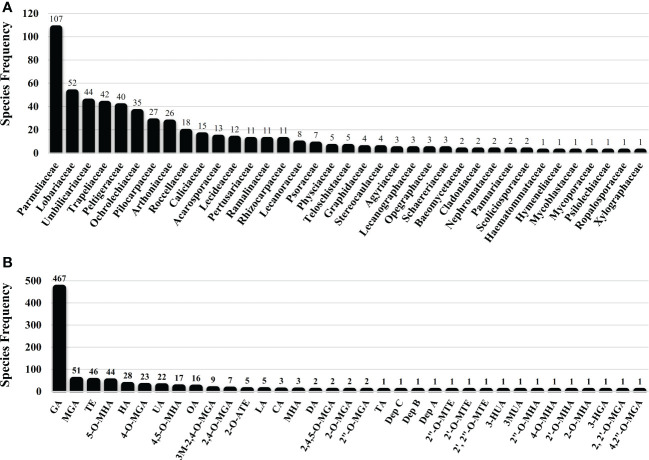
Lichen species frequency; **(A)** Family-based frequency of tridepside-producing lichen species reported in this study; **(B)** Frequency of tridepside-producing lichen species based on their detected tridepside; total number of tridepside-producing lichen species that we could find in the literature was 526; (Tridepsides’ abbreviations → GA: Gyrophoric acid/MGA: Methylgyrophoric acid/TE: Tenuiorin/5-O-MHA: 5-*O*-methylhiascic acid/HA: Hiascic acid/4-O-MGA: 4-*O*-methylgyrophoric acid/UA: Umbilicaric acid/4,5-O-MHA: 4,5-di-*O*-methylhiascic acid/OA: Ovoic acid/3M-2,4-O-MGA: 3-methoxy-2,4-di-*O*-methylgyrophoric acid/2,4-O-MGA: 2,4-di-*O*-methylgyrophoric acid/2-O-ATE: 2-*O*-acetyltenuiorin/LA: Lasallic acid/CA: Crustinic acid/MHA: Methylhiascic acid/DA: Deliseic acid/2,4,5-*O*-MGA: 2,4,5-tri-*O*-methylhiascic acid/2-O-MGA: 2-*O*-methylgyrophoric acid/2”-O-MGA: 2”-*O*-methylgyrophoric acid/TA: Trivaric acid/Dep C: depsidellin C/Dep B: depsidellin B/Dep A: depsidellin A/2”-O-MTE: 2”-*O*-methyltenuiorin/2’-O-MTE: 2’-*O*-methyltenuiorin/2’, 2”-O-MTE: 2’,2”-di-*O*-methyltenuiorin/3-HUA: 3-hydroxyumbilicaric acid/3MUA; 3-methoxyumbilicaric acid/2”-O-MHA: 2’’-*O*-methylhiascic acid/4-O-MHA: 4-*O*-methylhiascic acid/2’-O-MHA: 2’-*O*-Methylhiascic acid/2-O-MHA: 2-*O*-methylhiascic acid/3-HGA: 3-hydroxygyrophoric acid/2, 2’-O-MGA: 2,2’-di-*O*-methylgyrophoric acid/4,2”-O-MGA: 4,2”-di-*O*-methylgyrophoric acid).

**Figure 4:**
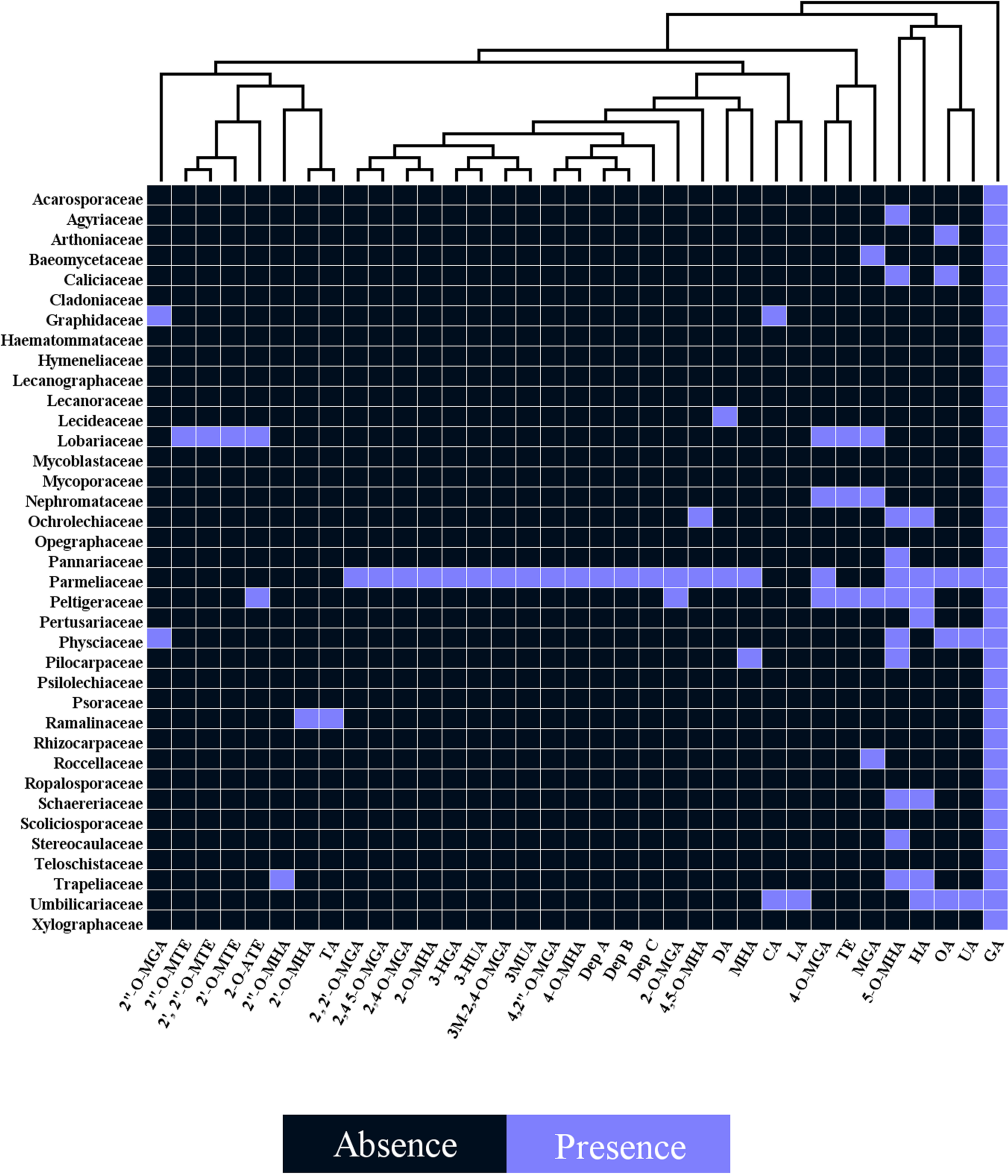
Heatplot of tridepside-producing lichen families. The plot shows the presence of different tridepsides in various lichen families (tridepsides’ abbreviations correspond to those of **Figure 3** caption).

Several studies have uncovered the main species of lichen producing tridepside. We found 526 species of lichen that contain at least one of the many lichenic tridepsides. The list of the species, their specific tridepsides, and the corresponding references can be found in our online resources (ESM-2, **Table S7**). The literature suggests that at least 35 lichenic tridepsides have been described (**Figure 3B**). As shown here, GA is the most commonly detected tridepside in species from 37 lichen families as it has been detected in 467 lichen species out of 526 (**Figure 3A**). It is worth mentioning that most of these species have been chemically investigated by TLC. Therefore, it is not surprising that other known tridepsides are found in fewer species than GA. If the chemistry of these lichens had been analyzed using more modern methods, other tridepsides than GA may have been detected in their thalli. The results also show that most tridepside-containing lichen species (107 out of 526) are from the family Parmeliaceae (**Figure 3A**). Lobariaceae, Umbilicariaceae, Trapeliaceae, Peltigeraceae, and Ochrolechiaceae are other lichen families with high numbers of tridepside-containing species (**Figure 3A**). Additionally, the heatplot in **Figure 4** shows the tridepsides reported from the various families. In Parmeliaceae, 25 out of 38 known lichenic tridepsides have been found, which is the highest. Both Lobariaceae and Peltigeraceae also exhibit high tridepside variation (8 tridepside out of 35 each). The distribution of GA among lichen families was also quite extensive. The heatplot (**Figure 4**) shows that GA has been found in all the tridepside-containing lichen families. It appears that 5-*O*-methylhiascic acid and HA are the other highly distributed tridepsides. Our online resources include a heatmap of the genera-based distribution of tridepsides (ESM-2, **Figure S1**). 111 genera of lichen contain at least one tridepside based on the heatplot. GA appears to have the highest distribution in these 111 genera. Other compounds, such as HA and 5-*O*-methylhiascic acid, also show high distribution among these genera. Furthermore, 24 genera of Parmeliaceae lichen family have been reported to produce tridepsides. Other lichen families with the most tridepside-producing genera include Lobariaceae (with seven genera), Roccellacae (with six genera), and Trapeliaceae (with six genera). At the same time, the tridepside variation observed in *Hypotrachyna*, a genus of Parmeliaceae, appears to be the highest, as 11 of the 35 known tridepsides occur in its species. Overall, in respect of tridepside variation, we could consider Parmeliaceae family the most prominent lichen family.

**Figure 5:**
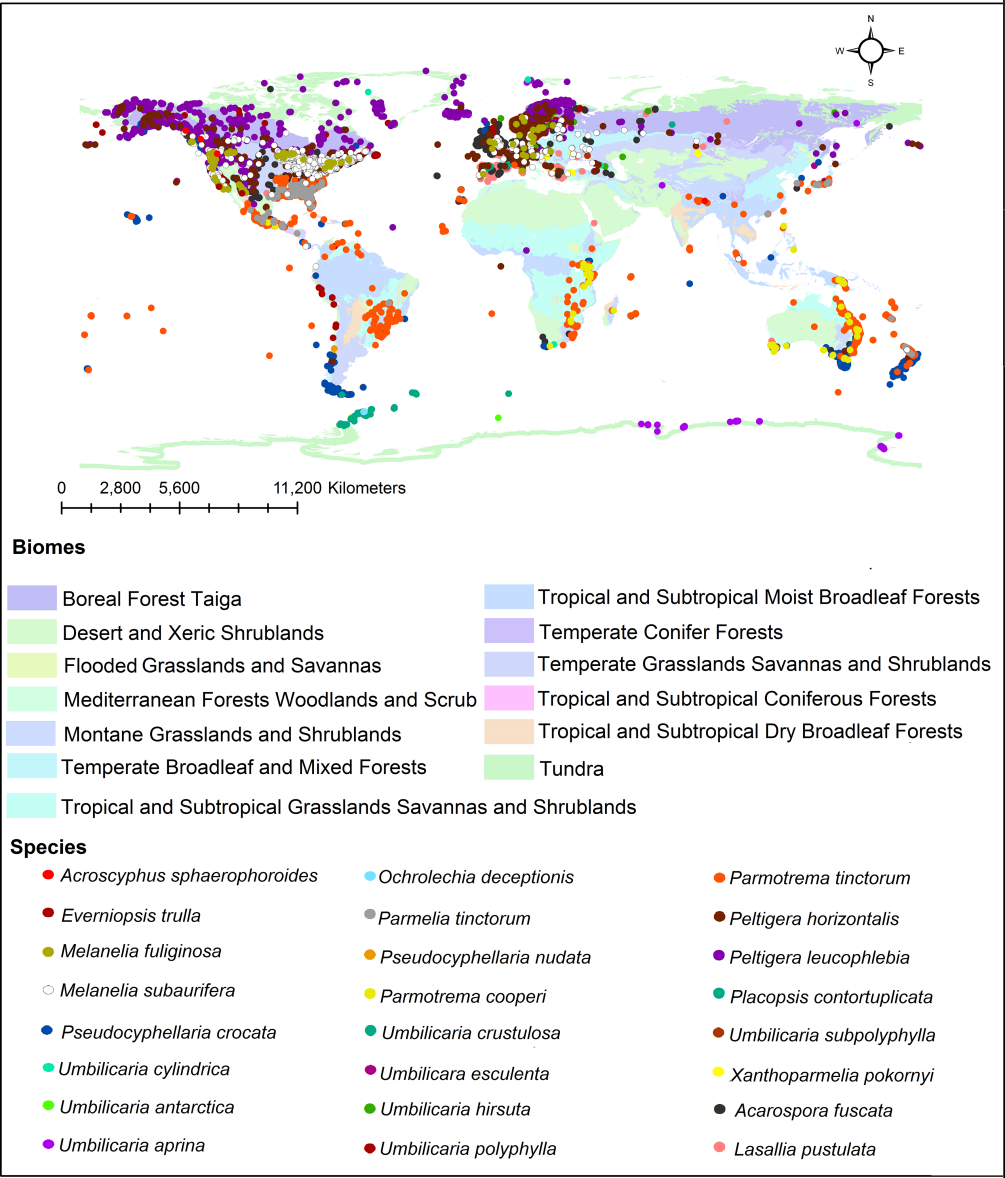
Global distribution of tridepside-containing lichen species based on the occurrence data from ALA, GBIF, BioCollecions, iNaturalists, and BISON databases.

In addition, we investigated the global distribution of 24 representative lichen species from which different tridepsides had been reported. The biome-based geographical distribution of these 24 representative lichen species is depicted in **Figure 5**. There are more occurrences in the northern hemisphere. In lichens, there are mainly 16 patterns of biogeographical distribution, as Galloway (2008) explains. Because of this, there are tangible differences in lichen species distribution. However, we must take into account that public species occurrence databases are not yet comprehensive enough. For example, although thousands of lichen species exist in Iran, such databases do not contain enough information on Iranian lichens’ occurrences. So, this is a gap that affects different aspects of species distribution patterns and because of this we cannot create a complete and precise pattern of tridepsides’ geographical distribution. However, this is where habitat suitability modeling and biogeography can be applied. These approaches can show us the richness and distribution range of different tridepside-containing species and the most important historical and environmental variables driving their global richness and distribution. Such approaches can also provide information on biodiversity hotspots for conservation planning if a species is detected as endangered. Thus, we believe that we can bridge the gaps in occurrence information of many species of lichen using machine learning modeling (i.e., species distribution modeling). This is a field that deserves more attention from lichen specialists.

**Figure 6:**
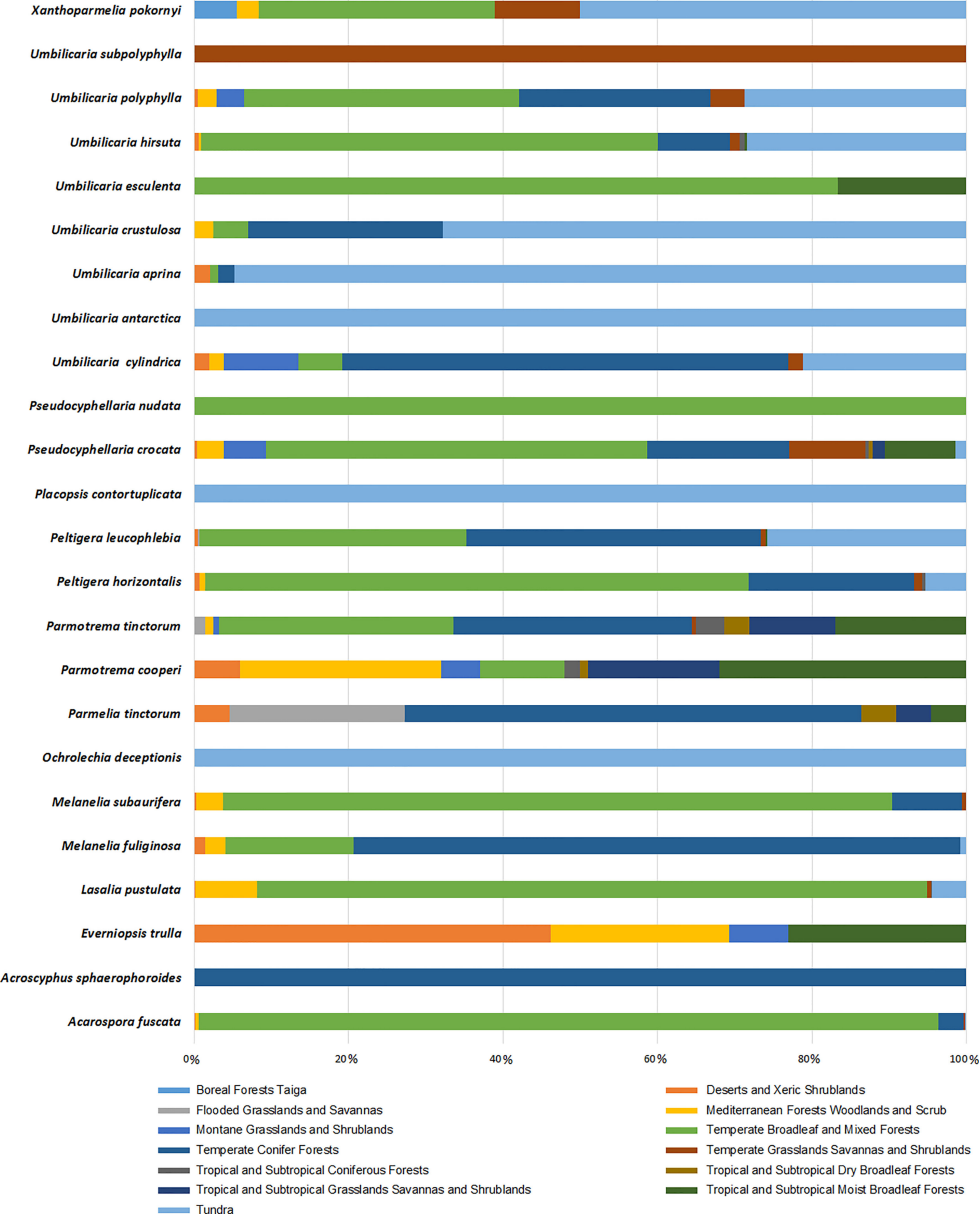
Biome-based occurrence of tridepside-containing lichen species; each bar represents the percentage of geographical occurrence of one lichen species in different biomes defined by Olson et al. (2001); Updated scientiitalic>fic names from Mycobank are shown here as updated name (= synonymy): *Melanelixia fuliginosa* (=*Melanelia fuliginosa*); *Melanelixia subaurifera* (=*Melanelia subaurifera*); *Parmotrema tinctorum* (=*Parmelia tinctorum*).

We have demonstrated the occurrence percentage of each species in different biomes worldwide in **Figure 6**. There are more occurrences in the Temperate Broadleaf and Mixed Forests and Tundra biomes. Some species, including *Umbilicaria subpolyphylla*, *Umbilicaria antarctica*, *Pseudocyphellaria nudata*, *Placopsis contortuplicata*, *Ochrolechia deceptionis*, and *Acroscyphus sphaerophoroides*, indicated limited inter-biome distribution because their occurrences have only been reported from a specific biome. On the other hand, other species showed a wider distribution across biomes. As a result, considering this distribution pattern, tridepside biosynthesis seems unrelated to the Biome-based distribution of the lichen species that produce them. For example, *Parmotrema cooperi* is found in 8 out of the 13 biomes depicted in **Figure 6**. This might suggest that the presence or absence of tridepsides is not strongly correlated with the biome type. Miralles et al. (2015) found widespread evidence for their study’s weak correlation between lichenochemicals and habitat. They investigated the lichenic substances of various lichen species from six different climatic regions. Their results indicated that lichenic substances did not confer an environment-specific fitness advantage. They also assert that common lichenic compounds, such as lecanoric acid and gyrophoric acid, seem to be synthesized by different lichen species in varying climate zones. This suggests a lack of evidence for region-specific biosynthesis of tridepsides. Nevertheless, the hypothesis of a weak correlation between lichenochemicals and lichen species’ habitat calls for further research. In this study we could gather evidence of tridepside production in 526 lichen species. To investigate any hypothesis related to biome- or region-based distribution of tridepsides we need to analyze the occurrence and richness of all these 526 species. In that way, we will have more evidence to prove or reject the hypothesis.

### 6.2 Non-lichenic sources of tridepsides

Lichenochemicals are believed to be exclusively biosynthesized by the interwoven thalli of lichen species (Ranković and Kosanić, 2015), and only a finite number of them have been reported from non-lichenic origins (Elix and Stocker-Wörgötter, 2008). On the whole, the point of the occurrence of lichenic substances in other non-lichenic organisms could be broken down into two separate parts. Firstly, a few lichenic substances are reported from non-lichenic organisms, such as plants and non-lichenized fungi. For example, parietin, a lichenic anthraquinone, has been found in plants and non-lichenized fungi. Also, lecanoric acid, a lichenic depside, has been reported from non-lichenized fungi (Elix and Stocker-Wörgötter, 2008). Secondly, it has been established that other organisms possess counterparts of lichenic substances. It has been reported, for instance, that dibenzofurans produced most commonly by lichens can also be found in plants, myxomycetes, basidiomycetes, edible mushrooms, and marine organisms (Millot et al., 2016). Despite this, we know that the polyketide biosynthetic pathway contributes significantly to lichenic substances, which means organisms with the same biosynthetic pathway are likely to produce similar but not identical compounds.

**Figure 7:**
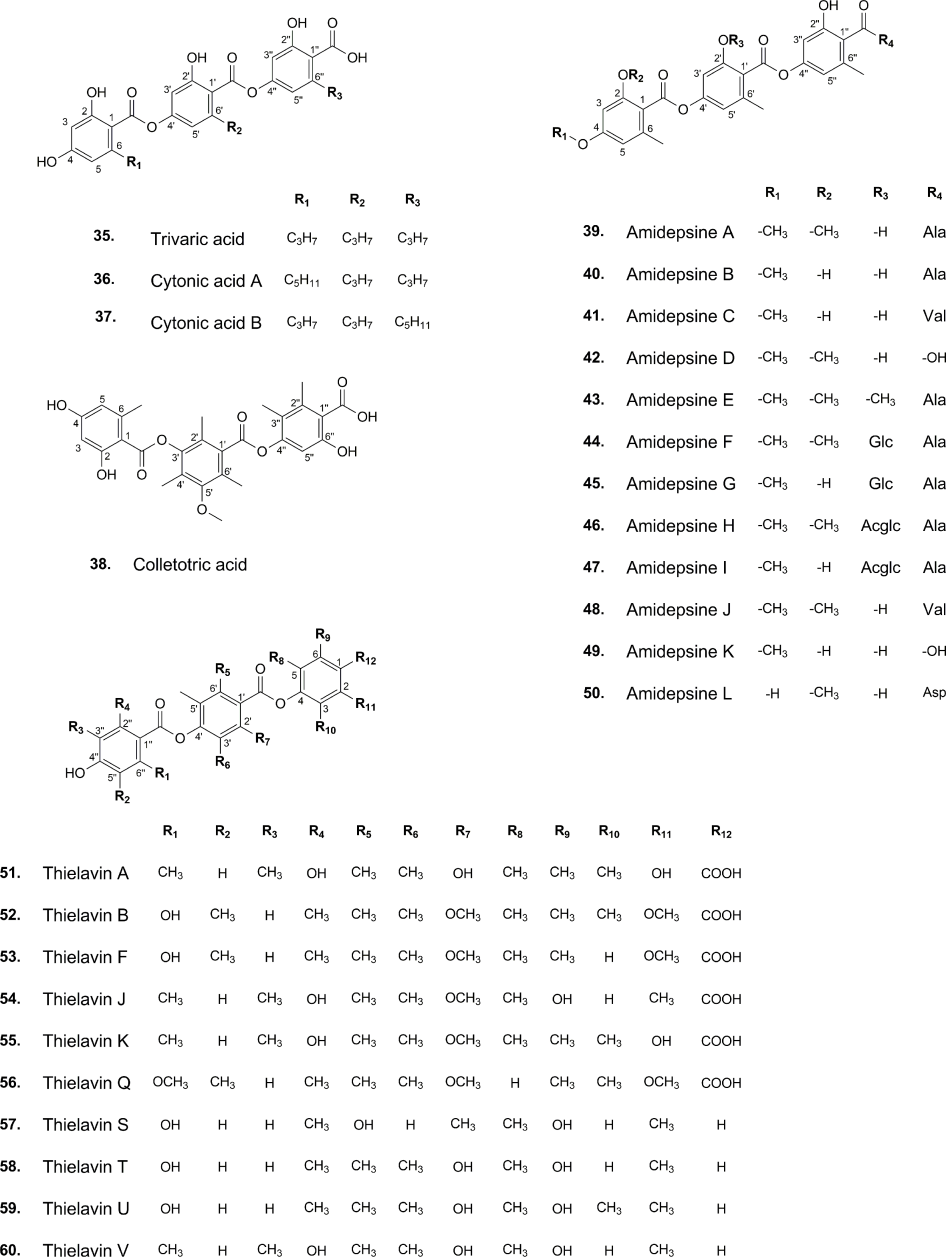
Chemical structures of reported non-lichenic tridepsides.

The development of analytical techniques and new methods has led to the detection of trace amounts of various substances found in natural sources. Literature review, for instance, offers compelling insights into tridepsides of non-lichenic origin. Evidence shows that some endophytic fungi can biosynthesize tridepsides similar to those produced by lichens. In the wake of recent findings from many studies, a list containing novel tridepsides detected in various endophytic fungi or microbial-based fungi and their chemical structures are presented in **Table 1** and **Figure 7**, respectively. Among the recently reported tridepsides of non-lichenic origin are cytonic acid A and B, trivaric acid, colletotric acid, thielavins, and amidepsines. Culberson et al. (1999) detected trivaric acid in the thallus of *Ramalina americana* using TLC and HPLC, but this tridepside has also been detected in a fungal strain from a soil sample and of microbial origin (**Table 1**). Tugizimana et al. (2019) detected colletotric acid in the secretions of *Colletotrichum sublineolum* (updated name *colletotrichum sublineola*; Mycobank) using UHPLC-MS. They have asserted that colletotric acid seems to be a mycotoxin that provides the fungus with convenient nutritional accessibility. *Colletotrichum* is a genus of more than 600 pathogenic plant fungi that affect many plants (Carbú et al., 2020). There is also a group of non-lichenic tridepsides known as thielavins from different sources reported in several studies. Thielavins A and B were found in cultures of the fungus *Thielavia terricola* (updated name *Pseudothielavia terricola*; Mycobank) by Kitahara et al. (1981); both compounds inhibited prostaglandin biosynthesis. Thielavins B, F, and Q were isolated from a soil fungus culture broth named *Coniochaeta* sp. 10F058 and indicated acceptable inhibitory activity against Indoleamine 2,3-dioxygenase, a protein that is involved in cancer and many neurological disorders (Jang et al., 2014). Thielavins A, J, and K were isolated from the culture broth of an endophytic fungus (MEXU 27095), and all showed promising anti-α-glucosidase activity in a concentration-dependent manner (Rivera-Chávez et al., 2013). Besides this, thielavins S, T, U, and V were also found in an endophytic fungus solid media (*Setophoma* sp.) (Rivera-Chávez et al., 2013).

**Table 1:** An overview of the non-lichenic tridepsides reported over the past few years.

Tridepside	Source	Bioactivity results	Ref.
Cytonic acid A	Endophytic fungus: *Cytonaema* sp.	Human cytomegalovirus protease inhibitor (IC_50_: 43 μmol)	(Guo et al., 2000)
Cytonic acid B	Endophytic fungus: *Cytonaema* sp.	Human cytomegalovirus protease inhibitor (IC_50_ of 11 μmol)	(Guo et al., 2000)
Trivaric acid	Soil fungus	Human leukocyte elastase inhibitor (IC_50_: 1.8 μM)	(Zheng et al., 2012)
	Microbial origin-based	Anti-diabetic: protein tyrosine phosphatase 1b (IC_50_: nM)	(Sun et al., 2017)
Colletotric acid	Endophytic fungus: liquid culture of *Colletotrichum gloeosporioides*	Antimicrobial: inhibited the growth of *Bacillus subtilis* (MIC: 25 μg/mL), *Staphylococcus aureus* (MIC: 50 μg/mL), and *Sarcina lutea* (MIC: 50 μg/mL)	(Zou et al., 2000)
Thielavin A	Endophytic fungus: MEXU 27095 isolated from *Hintonia latiflora*	Anti-diabetic: inhibited *Saccharomyces cerevisieae* α-glucosidase (aGHY) (IC_50_: 23.8 μM)	(Rivera-Chávez et al., 2013)
Thielavin B	Soil fungus: *Coniochaeta* sp. 10F058	Indoleamine 2,3-dioxygenase inhibition: (IC_50_: 21.2 μM)	(Jang et al., 2014)
Thielavin F	Soil fungus: *Coniochaeta* sp. 10F058	Indoleamine 2,3-dioxygenase inhibition: (IC_50_: 14.5 μM)	(Jang et al., 2014)
Thielavin J	Endophytic fungus: MEXU 27095 isolated from *Hintonia latiflora*	Anti-diabetic: inhibited *Saccharomyces cerevisiae* α-glucosidase (aGHY) (IC_50_: 15.8 μM)	(Rivera-Chávez et al., 2013)
Thielavin K	Endophytic fungus: MEXU 27095 isolated from *Hintonia latiflora*	Anti-diabetic: inhibited *Saccharomyces cerevisiae* α-glucosidase (aGHY) (IC_50_: 22.1 μM)	(Rivera-Chávez et al., 2013)
Thielavin Q	Soil fungus: *Coniochaeta* sp. 10F058	Indoleamine 2,3-dioxygenase inhibition: (IC_50_: 26.5 μM)	(Jang et al., 2014)
Thielavin S	Endophytic fungus: *Setophoma* sp (CML 2328) isolated from *Psidium guajava* fruits	Anti-bacterial: inhibited the growth of *Staphylococcus aureus* (MIC: 100 μg/mL)	(De Medeiros et al., 2015)
Thielavin T	Endophytic fungus: *Setophoma* sp (CML 2328) isolated from *Psidium guajava* fruits	Anti-bacterial: inhibited the growth of *Staphylococcus aureus* (MIC: 6.25 μg/mL)	(De Medeiros et al., 2015)
Thielavin U	Endophytic fungus: *Setophoma* sp (CML 2328) isolated from *Psidium guajava* fruits	Anti-bacterial: inhibited the growth of *Staphylococcus aureus* (MIC: 50 μg/mL)	(De Medeiros et al., 2015)
Thielavin V	Endophytic fungus: *Setophoma* sp (CML 2328) isolated from *Psidium guajava* fruits	Anti-bacterial: inhibited the growth of *Staphylococcus aureus* (MIC: 25 μg/mL)	(De Medeiros et al., 2015)
Amidepsine A	Soil fungus: *Humicola* sp. FO-2942	Anti-obesity: Inhibition of diacylglycerol acyltransferase (IC_50_: 10.2 μM)	(Tomoda et al., 1995a; Tomoda et al., 1995b)
Amidepsine B	Soil fungus: *Humicola* sp. FO-2942	Anti-obesity: Inhibition of diacylglycerol acyltransferase (IC_50_: 19.2 μM)	(Tomoda et al., 1995a; Tomoda et al., 1995b)
Amidepsine C	Soil fungus: *Humicola* sp. FO-2942	Anti-obesity: Inhibition of diacylglycerol acyltransferase (IC_50_: 51.6 μM)	(Tomoda et al., 1995a; Tomoda et al., 1995b)
Amidepsine D	Soil fungus: *Humicola* sp. FO-2942	Anti-obesity: Inhibition of diacylglycerol acyltransferase (IC_50_: 17.5 μM)	(Tomoda et al., 1995a; Tomoda et al., 1995b)
Amidepsine E	Soil fungus: *Humicola* sp. FO-5969	Anti-obesity: Inhibition of diacylglycerol acyltransferase (IC_50_: 124 μM)	(Tomoda et al., 1996)
Amidepsine F Amidepsine G Amidepsine H Amidepsine I Amidepsine J Amidepsine K Gyrophoric acid	Soil fungus: *Humicola* sp. FO-2942	–	(Inokoshi et al., 2010)
Amidepsine L	Endophytic fungus: *Trichocladium* sp. isolated from roots of *Houttuynia cordata*	–	(Tran-Cong et al., 2019)
Tenuiorin	liverwort: *Frullania nisquallensis*	–	(Kim et al., 1996)
	liverwort: *Bazzania tricrenata*	–	(Asakawa et al., 2008)
	liverwort: *Blasia pusilla*	–	(Yoshida et al., 1996)

a NMR, nuclear magnetic resonance; MS, mass spectrometry; HPLC, high-performance liquid chromatography; IR, infrared spectroscopy; HRMS, high-resolution mass spectrometry.

b IC_50_: half maximal inhibitory concentration; MIC, minimum inhibitory concentration.

In addition, amidepsines, which are primarily composed of a tridepside and an amino acid moiety, were first isolated from the culture broth of a fungal strain named *Humicola* sp. FO-2942, which was separated from a soil sample (Tomoda et al., 1995a). There have so far been 12 amidepsines reported from *Humicola* sp. FO-2942, *Humicola* sp. FO-5969, and *Trichocladium* sp. (**Table 1**). Many amidepsines studied have been found to have inhibitory activity against diacylglycerol acyltransferase, an enzyme essential in lipid metabolism (Shi and Burn, 2004; Tomoda and Omura, 2007). As noted by Tomoda et al. (1995b), amidepsines can be synthesized by taking either L-alanine or L-valine as their amino acid moiety (Inokoshi et al., 2010), except for amidepsine D, which lacks an amino acid moiety and whose structure represents 2,4-di-*O*-methylgyrophoric acid (Tomoda et al., 1995b) (**Figure 7**). Later, Tomoda et al. (1996) could isolate amidepsine E from the culture broth of *Humicola* sp. FO-5969. As for the latter strain, it was only capable to biosynthesize amidepsine A and amidepsine E. In another study by Inokoshi et al. (2010), six new amidepsines were isolated from the culture broth of *Humicola* sp. FO-2942 after static fermentation. Amidopsines F-I also exhibited an additional sugar moiety at their C-11 position, either D-glucose or 2-*O*-acetyl-D-glucose (**Figure 7**). Also, gyrophoric acid was further detected in the chemical profile of *Humicola* sp. FO-2942 after static fermentation (Inokoshi et al., 2010). It is possible that the latter finding marks a biotechnological breakthrough since it describes the presence in a non-lichenized fungal strain of a well-known lichenic tridepside.

There have been other tridepsides found in the culture media of various fungi. For example, De Medeiros et al. (2011) could detect and isolate two unknown tridepsides (PubChem CIDs: 53327802 and 53327822) from the solid-media culture of an endophytic fungus, *Cladosporium uredinicola*, that was separated from *Psidium guajava* fruits. Using spectroscopic (NMR) and spectrometric (ESI-MS/MS) data, they confirmed the chemical structure of the compounds by chromatographing an ethanolic extract of the rice-media culture. The two tridepsides showed bacteriostatic activity against *Escherichia coli*, *Staphylococcus aureus*, *Pseudomonas aeruginosa*, and *Bacillus subtilis*. Moreover, Hawksworth et al. (1993) demonstrated the ability of some lichenicolous fungi to produce lichenic substances. Based on TLC fingerprints, it was determined that *Sclerococcum sphaerale* and *Skyttea nitschkei*, two different lichenicolous fungi, both contain gyrophoric acid.

In addition, previous reports of the presence of tenuiorin in various liverworts introduce a new level of complexity to our knowledge of tridepsides. Tenuiorin has so far been detected in *Frullania nisquallensis*, *Bazzania tricrenata*, and *Blasia pusilla* (Kim et al., 1996; Yoshida et al., 1996; Asakawa et al., 2008). The liverwort *Blasia pusilla* often forms symbiotic relationships with cyanobacterium *Nostoc* spp. (Yoshida et al., 1996).

It appears that the biosynthesis of tridepsides is not confined to lichenized fungi, and free microorganisms are also capable of biosynthesis. *Colletotrichum*, *Cytonaema*, *Setophoma*, *Thielavia*, and *Humicola* are fungal genera belonging to the phylum Ascomycota. These fungi have the same class, Sordariomycetes, but *Colletotrichum* is from the Glomerellales order and Glomerellaceae family. *Thielavia* belongs to the Melanosporales order and the Ceratostomataceae family, while *Humicola* comes from the Sordariales order and Chaetomiaceae family. *Setophoma* is a member of the class Dothideomycetes, Order Pleosporales, and Family Phaeosphaeriaceae (Mycobank). Therefore, these genera do not share much taxonomic overlap except for their phylum. On this basis, we can suggest that tridepside-synthesizing genes are widely distributed within the fungi kingdom. There is a new possibility for biotechnological applications because some endophytic fungi, pathogenic fungi, lichenicolous fungi, and liverworts contain different types of tridepsides. Due to several drawbacks, biotechnological efforts to produce lichenic substances *in-vitro* have not met the needs of large-scale production (Verma and Behera, 2015). However, non-lichenized fungi might provide a more convenient method of obtaining tridepsides.

## 7 Conclusions and future perspectives

Although lichens have been largely explored in respect of their chemical compounds in the past, many of these compounds have not yet been evaluated for their probable biological activities. Thus, in light of the increasing need for new pharmaceutical substances, researchers could comprehensively evaluate the biological activities of lichen secondary metabolites. Among the probable defensive products of lichen’s resistance mechanism are tridepsides. Several tridepsides, including lichenic and non-lichenic, have been reported to be biologically active. As the most thoroughly studied lichenic tridepside, GA exhibits antiproliferative activity and wound healing, photoprotection, antioxidant activity, anti-diabetes, cardiovascular, and DNA binding properties. Due to its efficacy at higher doses, it is not a potent antiproliferative and antioxidant compound. It has been demonstrated that TE has a possible anti-Alzheimer’s activity and that UA also has strong antioxidant properties. However, future studies should focus on other lichenic tridepsides, such as UA, HA, and CA, since they have not been fully studied for their potential therapeutic effects. Non-lichenic tridepsides also demonstrate antiviral, anti-diabetic, and anti-bacterial activities. These sources might provide biotechnologists with precursors for the production of important lichenic tridepsides as well as new sources of tridepsides for pharmacological applications. Thus, future research could focus on non-lichenic sources of tridepsides, such as the *Colletotrichum* fungal genus, which has more than 600 species. In addition, the literature review indicated that most bioanalytical methods used to extract and isolate tridepsides rely on classical approaches. Thus, we believe there is a need to study the extraction and isolation of tridepsides using much more modern methods. Putative identification of biosynthetic gene clusters of depsides and tridepsides (i.e., GA) offers another novel strategy for the large-scale production of lichen secondary metabolites. However, a better integration of genome mining and analytical chemistry is needed to remove the ambiguities discussed in this paper. This may entail a transition from traditional analytical techniques to more modern ones, such as high-resolution mass spectrometry instruments.

We showed that lichenic sources of tridepsides are not limited to a specific region or biome. Therefore, we did not find an association between species distribution and tridepside biosynthesis. Furthermore, GA showed the highest distribution among a substantial number of lichen species (467 out of 526). Parmeliaceae, Lobariaceae, and Peltigeraceae proved to be important lichen families regarding lichenic sources. These families indicated the highest tridepside variation. *Hypotrachyna* was suggested as a potential source of tridepsides in addition to families since many species in this genus might contain different tridepsides. As a result of recent bioanalytical advances, some tridepsides have now been isolated from sources other than lichen, including pathogenic fungi, non-lichenized fungi, lichenicolous fungi, endophytic fungi, and liverworts, all of which might be considered biotechnologically valuable. There are also reports of GA and TE from sources other than lichens. Such findings call for further research regarding the distribution of lichenic substances because they are not consistent with the long-held view that most lichenic substances are exclusively produced by lichens.

## Author contributions

HN collected, analyzed, and interpreted the data, generated the figures and tables, and wrote the manuscript. MS supervised the work, checked the integrity of lichenological part of the paper, and revised the paper. JB supervised the work, provided part of the information, checked the authenticity of the biochemistry part of the paper, and revised the paper. MY contributed to the biome-based distribution of species and provided some of the figures. All authors contributed to the article and approved the submitted version.

## Footnotes

1
https://scholar.google.com/

2
https://www.scopus.com/

3
https://pubmed.ncbi.nlm.nih.gov/

4
https://nhm2.uio.no/botanisk/lav/RLL/RLL.HTM

5
https://scifinder.cas.org/

6
https://www.ala.org.au/

7
https://www.idigbio.org/

8
https://www.gbif.org/

9
https://www.inaturalist.org/

10
https://www.mycobank.org

## Glossary

**Table:**

A2780	Human ovarian carcinoma cell line
A375	Melanoma cancer cell line
A549	Human lung cancer cell line
ALA	Atlas of Living Australia database
ASE	Accelerated Solvent Extraction
AT	AcylTransferase
AT1	Angiotensin
BISON	Biodiversity Information Serving Our Nation database
CA	Crustinic acid
CaV	Capillary Voltage
CE	Collision Energy
CPC	Centrifugal Partiotion Chromatography
CT	Capillary Temperature
CV	Cone Voltage
DA	Deliseic Acid
DAD	Diode Array Detector
DGF	Desolvation Gas Flow
DT	Desolvation Temperature
ESIV	Electron Spray Ioonization Voltage
FC	Flash Chromatography
Fem-X	Human skin cancer cell line
GBIF	Global Biodiversity Information Facility database
HaCaT	Human immortal keratinocytes cell line
HDF	Human Dermal Fibroblast cells
HeLa	Human cervical cancer cell line
HL-60	Promyelocytic leukemia cell line
HPLC	High-Performance Liquid Chromatography
HPTLC	High-Performance Thin Layer Chromatography
HRE	Heat Reflux Extraction
HRMS	High-Resolution Mass Spectrometry
IL-MAE	Ionic Liquid-Microwave Assisted Extarction
IR	Infrared Spectroscopy
K562	Leukemia cell line
KS	KetoacylSynthase
LC-MS	Liquid chromatography-mass spectrometry
LPCC	Low-pressure column chromatography
LS174	Human colorectal cancer cell line
MAE	Microwave-Assisted Extraction
ME	Maceration Extraction
NMR	Nuclear Magnetic Resonance
PF-UVA	Protection Factor-Ultraviolet Radiation A
P-HPLC	Preparative-HPLC
P-TLC	Preparative-TLC
PTP1B	Protein Tyrosine Phosphatase 1B
RNS	Reactive Nitrogen Species
ROS	Reactive Oxygen Species
SE	Soxhlet Extraction
SFE	Supercritical Fluid Extraction
SGF	Sheath Gas Flow
SOD2	Superoxide Dismutase 2
SPE	Solid Phase Extraction
SPF	Sun Protection Factor
TLC	Thin Layer Chromatography
UAE	Ultrasound-Assisted Extraction
UHPLC	Ultra-high Performance Liquid Chromatography
UV-A	Ultraviolet Radiation A
UV-B	Ultraviolet Radiation B
