# Supplementary material for: Tridepsides as potential bioactives: a review on their chemistry and the global distribution of their lichenic and non-lichenic natural sources
Source: Front Fungal Biol. 2023 Apr 19;4:1088966. doi: 10.3389/ffunb.2023.1088966 (PMC10512237; doi:10.3389/ffunb.2023.1088966)
Supplement: Supplementary file 1 [file DataSheet_1.pdf]

Supplementary Material 1

list of supplementary data **page**

1. **Tridepside-mediated biological effects**.. **2**
2. **Antimicrobial activity of tridepsides**... **4**
3. **Extraction methodology of tridepsides** **5**
4. **Isolation of lichenic tridepsides**.. **6**
5. **Identification and quantification of tridepsides** **7**
6. **Chromatographic results of some hiascic acid isomers**............... **8**
7. **References** **9**

Table S1 - An overview of the tridepside-mediated biological effects along with the possible mechanism of action

Results or possible mechanism of action[a]	Compound [b] (effective dose or IC_{50})	Experimental model	Ref.
**Antiproliferative activity**			
GA reduced the cell viability of breast cancer cells from the MCF-7 cell line by 98%,	GA (IC_{50}: 478 µM)	colorimetric MTS assay; MCF-7 cells	(Mohammadi et al. 2022)
GA treatment increased the formation of ROSs (oxidative stress), DNA damage, caspase-3 activity, PARP cleavage, mitochondrial membrane damage, cell cycle arrest (indicated by increase in sub-G0/G1 DNA content), phosphatidyl serin externalization, and phosphorylation of stress/survival pathways proteins (MAPK, Erk and Akt) in HeLa cells.	GA (IC_{50}: 145.42 µM)	colorimetric MTS assay; HeLa cells	(Goga et al. 2019)
GA induced no increase in LDH release; GA increased the caspase-3 activity in high concentrations; GA increased the DNA damage through SCGE assay and TUNEL method, at high concentrations; GA increased the ROS formation in human cancer cell line at high concentrations; GA decreased the expression of Bcl-2, BAX and Hsp70 proteins at high concentrations	GA (25 and 50 µM)	A375 human melanoma cell line	(Cardile et al. 2017)
GA showed very weak activity against A549 and LS174 cells and exerted a moderate cytotoxic effect against Fem-x and K562 cells; GA treated cell lines indicated apoptosis of sub-G1 change in reducing the percentage of cells in G0/G1 and S-G2/M phases of the cell cycle	GA (IC_{50}= 64.01 and 78.45 µg/ml)	Human cell lines (FemX, A549, LS174 and K562)	(Kosanić et al. 2014)
GA decreased the MMP of A2780 at dose of 200 µM; GA significantly increased the proportion of Annexin V positive cells in HT-29 and A2780 cell lines which indicates the influence of GA on phosphatidylserine externalization; GA increased the ROS and RNS production in HT-29 cells at 200 µM; GA also increased the caspase-3 activity; GA induced the Cleavage of PARP in A2780 cells at higher doses; GA led to the down-regulation of p53 in the A2780 cancer cell line and increased the expression of p38 mitogen-activated protein kinases in A2780 cells; GA down-regulated the expression of Bcl-2 in A2780, Bcl-xL in HT-29, and BAX in both cell lines.	GA (100 and 200 µM)	A2780 (human ovarian carcinoma) and HT-29 (human colon adenocarcinoma) cancer cell lines	(Bačkorová et al. 2012)
GA indicated anti-proliferative or pro-apoptotic potential in HL-60 cell line at higher concentrations; GA inhibited the clonogenic activity of SK-BR-3 cells	GA (100 µM)	9 human cancer cell lines	(Bačkorová et al. 2011)
**Wound-healing**			
GA indicated wound closure activity in Scratch wounded HaCaT monolayers and its activity was in accordance with results of cell migration assay in which GA induced a high number of cell migrations	GA (180 µM)	human keratinocyte cell line (HaCaT)	(Burlando et al. 2009)
**Photoprotection**			
Sub-toxic doses of GA indicated photoprotective effects for the HaCaT cells toward UV-B; GA precluded the aftereffects of UV-B; GA increased the mitochondrial membrane damage, LDH release, and cytoskeleton damage	GA (100 µM)	human keratinocyte cell line (HaCaT)	(Varol et al. 2016)
GA indicated maximal UV absorbance in the UV-B range and molar extinction coefficient of 16000 L mol^{-1} cm^{-1}; no photosensitizing cytotoxicity was observed by GA	-	*in-vitro* assays; human keratinocyte cell line (HaCaT)	(Lohézic Le-Devehat et al. 2013)
**Anti-aging**			
GA increased the expression of COL1A1 and COL3A1 and decreased the expression of MMP1 by 77% in UV-A treated dermal fibroblasts; GA increased the production of type I collagen by 61% and reduced MMP1 protein by 33% in UV-A treated dermal fibroblasts; GA increased the expression of SOD2 by 2.2 times	GA (10 µg/mL)	Normal human dermal fibroblasts (HDF)	(Shim 2020)

Table S1 - (continued)

Results or possible mechanism of action[a]	Compound (effective dose or IC_{50})	Experimental model	Ref.
**Antioxidant**			
GA showed concentration dependent antioxidant activity	GA (30 % scavenging in 1 mg/mL)	DPPH radical scavenging capacity	(Shim 2020)
GA demonstrated very strong DPPH radical scavenging capacity; GA indicated the highest superoxide anion radical scavenging activity and reducing power	GA ($IC_{50=}$ 105.75 µg/ml and 196.62 µg/ml)	*in-vitro* antioxidant assays	(Kosanić et al. 2014)
GA and UA showed a dose-dependent DPPH radical scavenging capacity	GA, UA (0.1-5 mg/mL)	*in-vitro* antioxidant assays	(Buçukoglu et al. 2013)
**Cardiovascular effect**			
MD simulation indicated that GA is capable of forming key interactive residues with AT1	GA (IC_{50}: 29.76 µM)	calcium influx assay; HEK293/Ga15/AT1 cell line	(Huo et al. 2019)
**DNA interaction**			
GA indicated lower levels of hypochromism; LD spectra showed that GA can bind to DNA randomly; GA demonstrated Topo I inhibitory activity, while the relaxation of supercoiled DNA was completely inhibited; Topo II inhibition rate of GA was 100% at the concentration of 100 µM and showed no Topo II cleavage activity	GA (25 µM)	calf thymus DNA	(Plsíkova et al. 2014)
**Anti-diabetes**			
GA showed anti-glycation activity and inhibited the urease enzyme; SAR studies showed that the carbonyl and hydroxyl groups may possess anti-glycation activity	GA (IC_{50}: 777.46 µM)	*In-vitro* antiglycation (bovine serum albumin)	(Choudhary et al. 2011)
	GA (IC_{50}: 52.53 µM)	anti-urease assay (Indophenol method)	
GA inhibited the hydrolysis of the *p*NPP catalyzed by PTP1B in a dose-dependent manner; GA decreased the V_{max} value of PTP1B in substrate titration studies	GA (3.6 µM)	*In-vitro* PTP1B inhibitory assay	(Seo et al. 2009)
**Anti-Alzheimer's**			
TE formed hydrogen bonds with lysine and glutamine side chains of ^{306}VQIVYK^{311} motif; TE also showed hydrophobic interactions with valine and lysine side chains of ^{306}VQIVYK^{311} motif	TE (IC_{50}: 100 µM)	Thioflavin T fluorescence assay	(Salgado et al. 2020)

[a] Abbreviations → MCF-7: Human breast cancer cell line; ROS: reactive oxygen species; PARP: Poly (ADP-ribose) polymerase; MAPK: Mitogen-activated protein kinase; ERK: extracellular signal-regulated kinases; Akt: Protein kinase B; LDH: Lactate dehydrogenase; SCGE: single cell gel electrophoresis; TUNEL: Terminal deoxynucleotidyl transferase (TdT) dUTP Nick-End Labeling; Bcl-2: B-cell lymphoma 2; BAX: Bcl-2-associated X; Hsp70: 70 kilodalton heat shock protein; MMP: mitochondrial membrane potential; RNS: reactive nitrogen species; p53: tumor protein p53; Bcl-xL: B-cell lymphoma-extra large; COL3A1: Collagen, type III, alpha 1; COL1A1: Collagen, type I, alpha 1; MMP1: Matrix metalloproteinase-1; SOD2: Superoxide dismutase; DPPH: 2,2-diphenyl-1-picrylhydrazyl; MD: Molecular dynamics; AT1: Angiotensin II receptor type 1; LD: Linear dichroism; TOPO I: Topoisomerase I; TOPO II: Topoisomerase II; SAR: Structure-related studies; *p*NPP: para-Nitrophenylphosphate; PTP1B: Protein tyrosine phosphatase 1B

[b] GA: gyrophoric acid; UA: umbilicaric acid; TE: tenuiorin

Table S2 - Antimicrobial, anti-protozoal, and larvicidal activities mediated by different tridepsides reported recently

Microorganism	MIC/ inhibition/ $LC_{50,90}$[a]	Targeted compound[b]	Ref.
**Anti-bacterial**			
Aeromonas hydrophila	MIC: 1.87 mg/mL	GA	(Candan et al. 2006)
Bacillus cereus (NRRL B-3711)	MIC: 0.46 mg/mL	GA	(Candan et al. 2006)
Bacillus mycoides (ATCC 6462)	MIC: 0.03 mg/mL	GA	(Kosanić et al. 2014)
Bacillus mycoides (IPH 197)	MIC: 0.12 mg/mL	GA	(Kosanić and Ranković 2011)
Bacillus subtilis (ATCC 6633)	MIC: 0.01 mg/mL	GA	(Kosanić et al. 2014)
Bacillus subtilis (IPH 189)	MIC: 0.25 mg/mL	GA	(Kosanić and Ranković 2011)
Bacillus subtilis (NRRL B-744)	MIC: 0.46 mg/mL	GA	(Candan et al. 2006)
Enterobacter cloaceae (IPH 241)	MIC: 0.25 mg/mL	GA	(Kosanić and Ranković 2011)
Escherichia coli (ATCC 25922)	MIC: 0.31 mg/mL	GA	(Kosanić et al. 2014)
Escherichia coli (IPH 246)	MIC: 0.25 mg/mL	GA	(Kosanić and Ranković 2011)
Klebsiella pneumoniae (ATCC 13883)	MIC: 0.15 mg/mL	GA	(Kosanić et al. 2014)
Klebsiella pneumoniae (IPH 251)	MIC: 0.25 mg/mL	GA	(Kosanić and Ranković 2011)
Listeria monocytogenes	MIC: 1.87 mg/mL	GA	(Candan et al. 2006)
Proteus vulgaris (NRRL B-123)	MIC: 0.93 mg/mL	GA	(Candan et al. 2006)
Staphylococcus aureus (ATCC 25923)	MIC: 0.07 mg/mL	GA	(Kosanić et al. 2014)
Staphylococcus aureus (ATCC 43300)	MIC: 0.25 mg/mL	TE	(Celenza et al. 2013)
Staphylococcus aureus (AQ004SA)	MIC: 0.12 mg/mL	TE	(Celenza et al. 2013)
Staphylococcus aureus (AQ013SA)	MIC: >1.02 mg/mL	TE	(Celenza et al. 2013)
Staphylococcus aureus (AQ014SA)	MIC: >1.02 mg/mL	TE	(Celenza et al. 2013)
Staphylococcus aureus (IPH 221)	MIC: 0.25 mg/mL	GA	(Kosanić and Ranković 2011)
Staphylococcus aureus (ATCC 6538)	MIC: 3.74 mg/mL	GA	(Candan et al. 2006)
Staphylococcus haemolyticus (AQ007SH)	MIC: 0.06 mg/mL	TE	(Celenza et al. 2013)
Staphylococcus haemolyticus (AQ012SH)	MIC: 0.25 mg/mL	TE	(Celenza et al. 2013)
Staphylococcus warneri (AQ011SW)	MIC: 0.06 mg/mL	TE	(Celenza et al. 2013)
Staphylococcus warneri (AQ012SW)	MIC: 0.06 mg/mL	TE	(Celenza et al. 2013)
Streptococcus faecalis (NRRL B-14617)	MIC: 1.87 mg/mL	GA	(Candan et al. 2006)
Yersinia enterocolitica	MIC: 7.49 mg/mL	GA	(Candan et al. 2006)
**Anti-fungal**			
Aspergillus flavus (ATCC 9170)	MIC: 1.25 mg/mL	GA	(Kosanić et al. 2014)
	MIC: 0.5 mg/mL	GA	(Kosanić and Ranković 2011)
Aspergillus fumigatus (DBFS 310)	MIC: 0.62 mg/mL	GA	(Kosanić et al. 2014)
	MIC: 0.25 mg/mL	GA	(Kosanić and Ranković 2011)
Botrytis cinerea (DBFS 133)	MIC: 0.25 mg/mL	GA	(Kosanić and Ranković 2011)
Candida albicans (ATCC 10231)	MIC: 0.15 mg/mL	GA	(Kosanić et al. 2014)
Candida albicans (IPH 1316)	MIC: 0.25 mg/mL	GA	(Kosanić and Ranković 2011)
Candida albicans	MIC: 0.46 mg/mL	GA	(Candan et al. 2006)
Candida glabrata	MIC: 0.46 mg/mL	GA	(Candan et al. 2006)
Fusarium oxysporum (DBFS 292)	MIC: 0.25 mg/mL	GA	(Kosanić and Ranković 2011)
Mucor mucedo (ATCC 52568)	MIC: 0.25 mg/mL	GA	(Kosanić and Ranković 2011)
Paecilomyces variotii (ATCC 22319)	MIC: 0.25 mg/mL	GA	(Kosanić and Ranković 2011)
Penicillium purpurescens (DBFS 418)	MIC: 1.25 mg/mL	GA	(Kosanić et al. 2014)
	MIC: 0.5 mg/mL	GA	(Kosanić and Ranković 2011)
Penicillium verrucosum (DBFS 262)	MIC: 0.62 mg/mL	GA	(Kosanić et al. 2014)
	MIC: 0.5 mg/mL	GA	(Kosanić and Ranković 2011)
Trichoderma harsianum (DBFS 379)	MIC: 0.25 mg/mL	GA	(Kosanić and Ranković 2011)
**Trypanocidal**			
Trypanosoma cruzi	10 µM PGI (%)= 14<br>50 µM PGI (%)= 34	TE	(Fritis et al. 2013)
**Larvicidal**			
Culiseta longiareolata	LC_{50}= 0.41 ppm<br>LC_{90}= 1.93 ppm	GA	(Cetin et al. 2012)

[a] PGI: percentage of growth inhibition; LC_{50}: lethal concentration 50 %; LC_{90}: lethal concentration 90 %
[b] GA: gyrophoric acid, TE: tenuiorin

Table S3 - Extraction methodology of tridepsides from various lichen species

Source	Method[a]	Extraction methodology	Targeted compounds[b]	Ref.
Pseudocyphellaria crocata	ME	Sample powder (100 mg) → extracted thrice with acetone (500 µL) at room temp.	GA, TE	(Gadea et al. 2020)
Umbilicaria aprina	UAE	Lichen powder (5 g) → extracted twice with acetone (50 mL) in an ultrasonic bath for 30 min at 20 °C	GA, UA, HA	(Norouzi et al. 2020)
Peltigera horizontalis	UAE	Lichen powder (20 g) → extracted with acetone (150 mL) → 30 min in an ultrasound bath → the extract yield was 1.2 % of thallus dry weight after concentration	GA, TE	(Stojanović et al. 2020)
Umbilicaria subpolyphylla *Umbilicaria polyphylla*	ME	10 mg lichen powder → extracted with 1 mL methanol → for 3-5 days in darkness at room temp.	GA, UA, HA, CA	(Davydov et al. 2019)
Umbilicaria hirsuta	ME	Lichen powder (5 g) → extracted twice with acetone (50 mL) → for 24 h	GA	(Goga et al. 2019)
Parmotrema tinctorum	UAE	Lichen powder (5 mg) → extracted with 1 mL of different solvents of increasing polarity	GA	(Kumar et al. 2018)
Umbilicaria antarctica	ME	lichen powder (50 g) → extracted thrice with MeOH (500 mL) → 3 g dry extract resulted after concentration	GA, TE	(Salgado et al. 2020)
Everniopsis trulla	ME	Lichen powder (60 g) → extracted thrice with MeOH (500 mL) → 4 g dry extract resulted after concentration	GA	(Salgado et al. 2020)
Everniopsis trulla	UAE	Lichen powder (10 g) → extracted thrice with methanol (100 mL) → 30 min in an ultrasound bath at room temperature	GA	(Castro et al. 2017)
Umbilicaria crustulosa *Umbilicaria cylindrica*	UAE	Lichen powder (2 g) → extracted with 10 mL of different solvents (ether, ethyl acetate and dichloromethane) → 30 min in ultrasound bath	GA	(Zlatanovic et al. 2017)
Streptomyces sp. IB 2014/I/78-8.	LLE	Liquid cultures of actinobacteria strain → extracted with ethyl acetate → ratio of 1:1/ Biomass → extracted with an acetone:methanol mixture (ratio 1:1)/ Solid nutrient media → homogenized → extracted with an acetone:methanol mixture	GA	(Axenov-Gribanov et al. 2016)
Umbilicaria esculenta	ME	Lichen powder (38.8 g) → extracted thrice with acetone → for 2 days at room temp.	GA	(Nakashima et al. 2016)
Melanelia subaurifera *Melanelia fuliginosa*	SE	Lichen powder (50 g) → extracted with acetone using Soxhlet apparatus	GA	(Ristić et al. 2016)
Xanthoparmelia pokornyi	UAE	Lichen powder (10 g) → extracted with acetone using an ultrasound bath for 1 h → the extraction solution left at room temperature overnight	GA	(Varol et al. 2016)
Acarospora fuscata	SE	Lichen powder (100 g) → extracted with acetone using Soxhlet apparatus	GA	(Kosanić et al. 2014)
U. aprina var. *halei*	UAE	Lichen powder (10 g) → extracted with 100 mL of solvent (methanol, acetone, chloroform) → 30 min in an ultrasound bath	GA, UA	(Buçukoglu et al. 2013)
Lasallia pustulata	HRE	Lichen powder (4.5 g) → extracted with 4.5 mL of acetone in 3 reflux phases	GA	(Lohézic Le-Devehat et al. 2013)
Xanthoparmelia pokornyi	UAE	Lichen powder (10 g) → extracted with 10 mL of acetone → using an ultrasound bath for 1 h	GA	(Cetin et al. 2012)
Parmotrema cooperi	ME	Lichen powder → extracted with 2 L of 80% ethanol → 250 g extract	GA	(Choudhary et al. 2011)
Umbilicaria polyphylla	SE	Lichen powder (50 g) → extracted with acetone, methanol, and water → using a Soxhlet apparatus	GA	(Kosanić and Ranković 2011)
Lassalia pustulata	ME	Lichen powder → extracted with acetone at room temp.	GA	(Burlando et al. 2009)
Umbilicaria antarctica	ME	Lichen powder (10 g) → extracted twice with 300 mL of MeOH for 24 h	GA	(Seo et al. 2009)
Pseudocyphellaria nudata	ME	Lichen powder → extracted successively with chloroform and acetone (2 L) → 72 h each at room temp.	TE	(Cuellar et al. 2008)

[a] ME: maceration extraction; UAE: ultrasound assisted extraction; LLE: liquid-liquid extraction; SE: Soxhlet extraction

[b] GA: gyrophoric acid; TE: teuiorin; CA: crustinic acid; UA: umbilicaric acid; HA: hiascic acid

Table S4 - Methods of pre-concentration, isolation and purification of tridepsides in several studies over the past few years

Source	Method[a]	Procedure	Targeted compound (yield)[b]	Ref.
Parmotrema tinctorum	LPCC	Different sorbents were used (SiO_2, Al_2O_3, and RP C_{18}) → extracts were eluted using acetonitrile at room temp.	GA (NA)	(Kumar et al. 2018)
Ochrolechia deceptionis *Placopsis contortuplicata*	LPCC	Lichen extracts were isolated using silica gel	GA (NA)	(Cardile et al. 2017)
Umbilicaria antarctica	LPCC P-HPLC	MeOH extract was fractionated using Sephadex LH-20 → MP: MeOH → 3 fractions (A-C) → fraction A chromatographed → MP: *n*-hexane/EtOAc → yielded TE → fraction B → P-HPLC → MP: H2O/ MeOH in gradient mode → yielded GA	GA (30 mg); TE (120 mg)	(Salgado et al. 2020)
Everniopsis trulla	LPCC P-HPLC	MeOH extract was fractionated using Sephadex LH-20 → GA was purified using P-HPLC	GA (70 mg)	(Salgado et al. 2020)
Streptomyces sp. IB 2014/I/78-8.	P-HPLC	Evaporation of extracts using rotary evaporator at 40 °C; residue was dissolved in 500 µL of pure methanol → extract was eluted by a gradient of acetonitrile and 0.1% ammonium formate solution in water → elution was performed at flow rate of 5 mL/min for 23 minutes → 23 fractions	GA (NA)	(Axenov-Gribanov et al. 2016)
Umbilicaria esculenta	Recrystallization	Mother liquor was recrystallized using acetone	GA (0.96 g)	(Nakashima et al. 2016)
Xanthoparmelia pokornyi	P-TLC	Extracts were separated on TLC plates using solvent systems A, C, and G → the substances were identified based on their R_f values	GA (NA)	(Varol et al. 2016)
Acarospora fuscata	LPCC	500 mg of acetone extract was dissolved in benzene → the residue was fractionated using a silica gel 60 column → MP: methanol chloroform gradient solvent (20:1, 10:1 and 5:1) → 9 fractions	GA (NA)	(Kosanić et al. 2014)
U. aprina var. *halei*	P-TLC	extract was separated by TLC plates using solvent system C (contained toluene/glacial acetic acid) → substances were identified based on their R_f values	GA, UA (NA)	(Buçukoglu et al. 2013)
Lasallia pustulata	Precipitation	Lichen powder (100 g) → extracted thrice using 500 mL of solvents with increasing polarity (n-heptane, dichloromethane, tetrahydrofuran) under reflux → GA was precipitated in the fraction of tetrahydrofuran	GA (NA)	(Lohézic Le-Devehat et al. 2013)
Xanthoparmelia pokornyi	P-TLC	347 mg of lichen extract → isolated using P-TLC plates	GA (18 mg)	(Cetin et al. 2012)
Parmotrema cooperi	LPCC	250 g ethanolic extract → fractionation using Silica gel column → ethyl acetate fraction was further eluted by *n*-hexane-ethyl acetate solution → 11 fractions → fractions 7-11 were subjected to LPCC → eluents: *n*-hexane-ethyl acetate solution	GA (NA)	(Choudhary et al. 2011)
Umbilicaria polyphylla	P-TLC	Extracts were separated on TLC plates using toluene:dioxane:vinegar acid (90:25:4)	GA (NA)	(Kosanić and Ranković 2011)
Umbilicaria antarctica	FC P-HPLC	1.1 g of MeOH extract → subjected to C18-functionalized silica gel flash column chromatography (3 × 15 cm) → eluents: MeOH in H2O (400 mL each) → 80% MeOH fraction (49.5 mg) → semi-preparative reversed-phase HPLC → eluents: acetonitrile + water with % formic acid	GA (13.1 mg)	(Seo et al. 2009)
Pseudocyphellaria nudata	LPCC	Chloroform extract (6.08 g) → silica gel column → eluents: dichloromethane and ethyl-acetate with increasing polarity (49:1 and 1:49) → 260 fractions 8 mL each → fractions 16-38 were combined based on TLC → evaporation	TE (1.61 g)	(Cuellar et al. 2008)
Peltigera leucophlebia.	LPCC	Acetonic extract ➔ chromatographed using chloroform and methanol	TE (179 mg)	(Ingólfsdóttir et al. 2002)

[a] MP: mobile phase; LPCC: low pressure column chromatography; P-HPLC: preparative-high performance liquid chromatography; FC: flash chromatography

[b] GA: gyrophoric acid; TE: tenuiorin; NA: not available

Table S5 - Identification and quantification approaches of tridepsides used over the past few years

Source	Column	Eluents[a]	Detection (parameters)[b]	Detected compound[c]	Ref.
**HPLC**					
Peltigera horizontalis	Zorbax Eclipse XDB-C18, 5 μm	Methanol: water: formic acid (80:20:0.2 v/v/v) / f.r.= 0.5 mL/min	DAD (190-400 nm)	GA, TE, MGA	(Stojanović et al. 2020)
Umbilicaria subpolyphylla *Umbilicaria polyphylla*	ZORBAX Eclipse Plus C18, narrowboreRR 2·1 × 150mm, 3·5 μm	A: water with 0·5% orthophosphoric acid/ B: methanol with 0·5% orthophosphoric acid/ f.r.: 1 mL/min	UV-Vis	GA, UA, HA, CA	(Davydov et al. 2019)
Umbilicaria hirsuta	7 μm Kromasil SGX C18	A: 5% acetonitrile + 1% (v/v) trifluoracetic acid)/ B: 80% acetonitrile/ f.r.= 0.7 mL/min	DAD	GA	(Goga et al. 2019)
Umbilicaria crustulosa *Umbilicaria cylindrica*	Agilent, Zorbax Eclipse XDB-C18, 5 μm, 4.6 × 150 mm	-	DAD	GA	(Zlatanovic et al. 2017)
Melanelia subaurifera *Melanelia fuliginosa*	C18; 25 cm × 4.6 mm, 10 μm	methanol–water-phosphoroc acid (85:15:0,9, v/v/v)/ f.r.= 1.0 mL/ min	UV	GA	(Ristić et al. 2016)
Acarospora fuscata	C18; 250 mm × 4.6 mm, 5 μm	Methanol–water–phosphoric acid (90:10:0.9, v/v/v)/ f.r.= 1.0 mL/ min	UV	GA	(Kosanić et al. 2014)
Umbilicaria hirsuta	Tessek SGX C18 5 μm (4 × 250 mm)	A: water: acetonitrile:H3PO4 (80:19:1)/ B: 95% acetonitrile/ f.r.= 0.7 mL/min	UV-Vis	GA	(Bačkorová et al. 2011)
Acroscyphus sphaerophoroides	Zorbax SB-C18 (4.6 × 250 mm, 5 μm)	A: aqua bidest containing 1% orthophosphoric acid/ B: methanol/ f.r.= 0.7 ml/min	PDA	GA	(Niu et al. 2008)
**LC-MS**					
Pseudocyphellaria crocata	Phenomenex®, Kinetex 2.6μ C18 100A	A: water + 0.1% formic acid/ B: acetonitrile + 0.1% formic acid/ f.r.= 0.5 mL.min	DAD-MS (CaV= 180 V; SV= 20 V; ESIV= 3.5 kV; NGP= 60 psig; DGF= 4 L/min; CT: 250 °C)	GA, TE	(Gadea et al. 2020)
Umbilicaria aprina	Atlantis T3 C18 (2.1 mm × 100 mm, 3 μm; Waters)	A: water + 0.1% formic acid, v/v/ B: acetonitrile/ f.r.= 0.25 mL/min	QqQ-MS (ST= 120 °C; DT= 300 °C; CaV= 3.5 kV; CV= 30 V; CE= 30 eV)	GA, UA, HA	(Norouzi et al. 2020)
Parmotrema tinctorum	Acquity BEH C18 column (100 mm × 2.1mm i.d., 1.7 μm)	A: water + 0.1% formic acid/ B: acetonitrile/ f.r.= 0.4 mL/min.	QToF-MS (ST= 120 °C; DT= 350 °C; CaV= 2 kV; CV= 30 V)	GA	(Kumar et al. 2018)
Everniopsis trulla	Acclaim, 150 mm × 4.6 mm ID, 5 μm, Thermo Fisher Scientific	A: 1% formic aqueous solution/ B: acetonitrile/ f.r.= 1.00 mL/min	Q-OT-MS (SGF= 75 units; CT= 400 °C; ESIV= 2500 V; CE= 30 kV)	GA	(Castro et al. 2017)
Streptomyces sp. IB 2014/I/78-8.	C18 column (Affymetrix, Santa Clara, USA)	A: acetonitrile/ B: 0.1% ammonium formate solution in water/ f.r.= 0.5 mL/min	QToF-MS (NA)	GA	(Axenov-Gribanov et al. 2016)

[a] f.r.: flow rate;

[b] DAD: Diode array detector; PDA: photodiode array detector; ST: Source Temperature; DT: Desolvation Temperature; CaV: Capillary Voltage; CV: Cone Voltage; CE: Collision Energy; SV: Source Voltage; ESIV: ESI Voltage; NGP: Nebulizer Gas Pressure; DGF: Desolvation Gas Flow; CT: Capillary Temperature; SGF: Sheath Gas Flow; NA: not available

[c] GA: gyrophoric acid; TE: tenuiorin, MGA: methylgyrophoric acid; UA: umbilicaric acid; HA: hiascic acid; CA: crustinic acid

Table S6 - Some chromatographic and spectrometric behaviors of tridepside isomers with similar NMR spectra (Elix 2014)

Compound	TLC R_f and color reaction of spot [a]						HPLC RI [b]	MS [c]	Spot-test results [d]
	A	B	B'	C	G	Spot color after acid spray (H_2SO_4)			
3-Hydroxyumbilicaric acid	16	-	26	11	46	pale yellow	16	198,181,168,150	KC (pink)
2-*O*-methylhiascic acid	11	-	20	8	39	pale orange, grey halo	17	413,383,196,168	No reaction
2'-*O*-methylhiascic acid	10	-	28	12	-	yellow	18	255,199,165	C (red)
4-*O*-methylhiascic acid	26	26	19	35	-	pale bright blue fading to pale yellow	19	198,196,180	C (red), KC (red)
5-*O*-methylhiascic acid	21	35	36	29	-	pale orange, grey halo	24	348,318,198	C (red)
2" -*O*-methylhiascic acid	NOT FOUND								

[a] Retention factor of compound in TLC analysis of lichen substances using standard solvent systems [A: toluene/ dioxane/ acetic acid (180: 45: 5); B: hexane/ diethyl ether/ formic acid (130: 80: 20); B': hexane/ methyl tert-butyl ether/ formic acid (140: 72: 18); C: toluene/ acetic acid (170: 30); G: toluene/ ethyl acetate/ formic acid (139: 83: 8)]

[b] Retention index of compound in standard HPLC procedure introduced for lichen substances (Elix 2014)

[c] *m/z* of fragments in mass spectrum

[d] Color reactions of lichen medulla with standard reagents→ C: Sodium hypochlorite solution; KC: Potassium hydroxide solution

References

Axenov-Gribanov, D. V. et al. 2016. Actinobacteria isolated from an underground lake and moonmilk speleothem from the biggest conglomeratic karstic cave in Siberia as sources of novel biologically active compounds. *PLoS ONE* 11(2). doi: 10.1371/journal.pone.0149216.

Bačkorová, M., Bačkor, M., Mikeš, J., Jendželovský, R. and Fedoročko, P. 2011. Variable responses of different human cancer cells to the lichen compounds parietin, atranorin, usnic acid and gyrophoric acid. *Toxicology in Vitro* 25(1), pp. 37–44. doi: 10.1016/j.tiv.2010.09.004.

Bačkorová, M., Jendželovský, R., Kello, M., Bačkor, M., Mikeš, J. and Fedoročko, P. 2012. Lichen secondary metabolites are responsible for induction of apoptosis in HT-29 and A2780 human cancer cell lines. *Toxicology in Vitro* 26(3), pp. 462–468. doi: 10.1016/j.tiv.2012.01.017.

Buçukoglu, T.Z., Albayrak, S., Halici, M.G. and Tay, T. 2013. Antimicrobial and antioxidant activities of extracts and lichen acids obtained from some umbilicaria species from central Anatolia, Turkey. *Journal of Food Processing and Preservation* 37(6), pp. 1103–1110. doi: 10.1111/j.1745-4549.2012.00811.x.

Burlando, B., Ranzato, E., Volante, A., Appendino, G., Pollastro, F. and Verotta, L. 2009. Antiproliferative effects on tumour cells and promotion of keratinocyte wound healing by different lichen compounds. *Planta Medica* 75(6), pp. 607–613. doi: 10.1055/s-0029-1185329.

Candan, M., Yilmaz, M., Tay, T., Kivanç, M. and Türk, H. 2006. Antimicrobial activity of extracts of the lichen *Xanthoparmelia pokornyi* and its gyrophoric and stenosporic acid constituents. *Zeitschrift fur Naturforschung - Section C Journal of Biosciences* 61(5–6), pp. 319–323. doi: 10.1515/znc-2006-5-603.

Cardile, V., Graziano, A.C.E., Avola, R., Piovano, M. and Russo, A. 2017. Potential anticancer activity of lichen secondary metabolite physodic acid. *Chemico-Biological Interactions* 263, pp. 36–45. doi: 10.1016/j.cbi.2016.12.007.

Castro, O.N., Benites, J., Rodilla, J., Santiago, J.C., Simirgiotis, M., Sepulveda, B. and Areche, C. 2017. Metabolomic Analysis of the Lichen *Everniopsis trulla* Using Ultra High Performance Liquid Chromatography-Quadrupole-Orbitrap Mass Spectrometry (UHPLC-Q-OT-MS). *Chromatographia* 80(6), pp. 967–973. doi: 10.1007/s10337-017-3304-4.

Celenza, G. et al. 2013. Antibacterial activity of selected metabolites from Chilean lichen species against methicillin-resistant staphylococci. *Natural Product Research* 27(17), pp. 1528–1531. doi: 10.1080/14786419.2012.730043.

Cetin, H., Tufan-Cetin, O., Turk, A.O., Tay, T., Candan, M., Yanikoglu, A. and Sumbul, H. 2012. Larvicidal activity of some secondary lichen metabolites against the mosquito *Culiseta longiareolata* Macquart (Diptera: Culicidae). *Natural Product Research* 26(4), pp. 350–355. doi: 10.1080/14786411003774296.

Choudhary, M.I., Ali, M., Wahab, A.T., Khan, A., Rasheed, S., Shyaula, S.L. and Rahman, A.U. 2011. New antiglycation and enzyme inhibitors from *Parmotrema cooperi*. *Science China Chemistry* 54(12), pp. 1926–1931. doi: 10.1007/s11426-011-4436-2.

Cuellar, M., Quilhot, W., Rubio, C., Soto, C., Espinoza, L. and Carrasco, H. 2008. Phenolics, depsides and triterpenes from the Chilean lichen *Pseudocyphellaria nudata* (Zahlbr.) D.J. Galloway. *Journal of the Chilean Chemical Society* 53(3), pp. 1624–1625. doi: 10.4067/S0717-97072008000300017.

Davydov, E.A., Blum, O.B., Kashevarov, G.P. and Grakhov, V.P. 2019. *Umbilicaria subpolyphylla* Oxner: The correct name for U. iberica Sancho & Krzewicka and its bipolar distribution pattern. *Lichenologist* 51(3), pp. 205–220. Available at: https://doi.org/10.1017/S002428291900015X [Accessed: 3 February 2021].

Elix, J.A. 2014. *A Catalogue of Standardized Chromatographic Data and Biosynthetic Relationships for Lichen Substances*. The.

Fritis, M.C. et al. 2013. Depsides and triterpenes in Pseudocyphellaria coriifolia (lichens) and biological activity against *Trypanosoma cruzi*. *Natural Product Research* 27(17), pp. 1607–1610. doi: 10.1080/14786419.2012.740033.

Gadea, A., Fanuel, M., Lamer, A.C. Le, Boustie, J., Rogniaux, H., Charrier, M. and Lohézic Le-Devehat, F. 2020. Mass spectrometry imaging of specialized metabolites for predicting lichen fitness and snail foraging. *Plants* 9(1). doi: 10.3390/plants9010070.

Goga, M., Kello, M., Vilkova, M., Petrova, K., Bačkor, M., Adlassnig, W. and Lang, I. 2019. Oxidative stress mediated by gyrophoric acid from the lichen *Umbilicaria hirsuta* affected apoptosis and stress/survival pathways in HeLa cells. *BMC Complementary and Alternative Medicine* 19(1). doi: 10.1186/s12906-019-2631-4.

Huo, X., Qiao, L., Chen, Y., Chen, X., He, Y. and Zhang, Y. 2019. Discovery of Novel Multi-target Inhibitor of angiotensin type 1 receptor and neprilysin inhibitors from Traditional Chinese Medicine. *Scientific Reports* 9(1). doi: 10.1038/s41598-019-52309-z.

Ingólfsdóttir, K., Gudmundsdóttir, G.F., Ögmundsdóttir, H.M., Paulus, K., Haraldsdóttir, S., Kristinsson, H. and Bauer, R. 2002. Effects of tenuiorin and methyl orsellinate from the lichen *Peltigera leucophlebia* on 5-/15-lipoxygenases and proliferation of malignant cell lines in vitro. *Phytomedicine* 9(7), pp. 654–658. doi: 10.1078/094471102321616481.

Kosanić, M. and Ranković, B. 2011. Antioxidant and antimicrobial properties of some lichens and their constituents. *Journal of Medicinal Food* 14(12), pp. 1624–1630. doi: 10.1089/jmf.2010.0316.

Kosanić, M., Ranković, B., Stanojković, T., Vasiljević, P. and Manojlović, N. 2014. Biological activities and chemical composition of lichens from Serbia. *EXCLI Journal* 13, pp. 1226–1238. Available at: https://www.ncbi.nlm.nih.gov/pmc/articles/PMC4462831/ [Accessed: 1 January 2019].

Kumar, K. et al. 2018. UPLC–MS/MS quantitative analysis and structural fragmentation study of five Parmotrema lichens from the Eastern Ghats. *Journal of Pharmaceutical and Biomedical Analysis* 156, pp. 45–57. Available at: https://hal-univ-rennes1.archives-ouvertes.fr/hal-01807884 [Accessed: 26 June 2020].

Lohézic Le-Devehat, F., Legouin, B., Couteau, C., Boustie, J. and Coiffard, L. 2013. Lichenic extracts and metabolites as UV filters. *Journal of Photochemistry and Photobiology B: Biology* 120, pp. 17–28. doi: 10.1016/j.jphotobiol.2013.01.009.

Mohammadi, M., Zambare, V., Suntres, Z. and Christopher, L. 2022. Isolation, Characterization, and Breast Cancer Cytotoxic Activity of Gyrophoric Acid from the Lichen *Umbilicaria muhlenbergii*. *Processes* 10(7), p. 1361. Available at: https://www.mdpi.com/2227-9717/10/7/1361/htm [Accessed: 2 November 2022].

Nakashima, K. ichi, Tanabe, H., Fujii-Kuriyama, Y., Hayashi, H. and Inoue, M. 2016. Atranorin and lecanoric acid antagonize TCDD-induced xenobiotic response element-driven activity, but not xenobiotic response element-independent activity. *Journal of Natural Medicines* 70(3), pp. 476–482. doi: 10.1007/s11418-016-0983-3.

Niu, D.-L., Wang, L.-S., Zhang, Y.-J. and Yang, C.R. 2008. *Acroscyphus sphaerophoroides* (lichenized Ascomycota, Caliciaceae) in Hengduanshan Mountains. *Biochemical Systematics and Ecology* 36(5–6), pp. 423–429. doi: 10.1016/j.bse.2008.01.009.

Norouzi, H., Azizi, A., Gholami, M., Sohrabi, M. and Boustie, J. 2020. Chemotype variations among lichen ecotypes of *Umbilicaria aprina* as revealed by LC-ESI-MS/MS: a survey of antioxidant phenolics. *Environmental Science and Pollution Research* 27(32), pp. 40296–40308. Available at: https://link.springer.com/article/10.1007/s11356-020-10053-2 [Accessed: 17 July 2020].

Plsíkova, J., Stepankova, J., Kasparkova, J., Brabec, V., Bačkor, M. and Kozurkova, M. 2014. Lichen secondary metabolites as DNA-interacting agents. *Toxicology in Vitro* 28(2), pp. 182–186. doi: 10.1016/j.tiv.2013.11.003.

Ristić, S. et al. 2016. Phytochemical study and antioxidant, antimicrobial and anticancer activities of *Melanelia subaurifera* and *Melanelia fuliginosa* lichens. *Journal of Food Science and Technology* 53(6), pp. 2804–2816. doi: 10.1007/s13197-016-2255-3.

Salgado, F., Caballero, J., Vargas, R., Cornejo, A. and Areche, C. 2020. Continental and Antarctic Lichens: isolation, identification and molecular modeling of the depside tenuiorin from the Antarctic lichen *Umbilicaria antarctica* as tau protein inhibitor. *Natural Product Research* 34(5), pp. 646–650. doi: 10.1080/14786419.2018.1492576.

Seo, C., Choi, Y.H., Ahn, J.S., Yim, J.H., Lee, H.K. and Oh, H. 2009. PTP1B inhibitory effects of tridepside and related metabolites isolated from the Antarctic lichen *Umbilicaria antarctica*. *Journal of Enzyme Inhibition and Medicinal Chemistry* 24(5), pp. 1133–1137. doi: 10.1080/14756360802667811.

Shim, J.H. 2020. Anti-Aging Effects of Gyrophoric Acid on UVA-Irradiated Normal Human Dermal Fibroblasts. *Natural Product Communications* 15(4). doi: 10.1177/1934578X20919545.

Stojanović, G., Zrnzević, I., Zlatanović, I., Stanković, M., Stankov Jovanović, V., Mitić, V. and Đorđević, A. 2020. Chemical profile and biological activities of *Peltigera horizontalis* (Hudson) Baumg. thallus and apothecia extracts. *Natural Product Research* 34(4), pp. 549–552. doi: 10.1080/14786419.2018.1489386.

Varol, M., Türk, A., Candan, M., Tay, T. and Koparal, A.T. 2016. Photoprotective Activity of Vulpinic and Gyrophoric Acids Toward Ultraviolet B-Induced Damage in Human Keratinocytes. *Phytotherapy Research* 30(1), pp. 9–15. doi: 10.1002/ptr.5493.

Zlatanovic, I., Zrnzevic, I., Jovanovic, O., Stojanovic, I., Petrovica, G. and Stojanovica, G. 2017. Chemical composition of *umbilicaria crustulosa* and *U. cylindrica*. *Natural Product Communications* 12(7), pp. 1105–1106. doi: 10.1177/1934578x1701200726.
