# Supplementary material for: Tridepsides as potential bioactives: a review on their chemistry and the global distribution of their lichenic and non-lichenic natural sources
Source: Front Fungal Biol. 2023 Apr 19;4:1088966. doi: 10.3389/ffunb.2023.1088966 (PMC10512237; doi:10.3389/ffunb.2023.1088966)
Supplement: Supplementary file 2 [file DataSheet_2.pdf]

Supplementary Material 2

list of supplementary data **Page**

1. **List of tridepside-containing lichen species (Table S7)...........2**
2. **Heatplot of lichen genera and their tridepsides.......................25**
3. **References...26**

Table S7 - Species-based distribution of tridepsides from lichens

Species	Family	Order	Class	Identification method	Ref.
**2"-*O*-methylgyrophoric acid**					
Diploschistes gyrophoricus	Graphidaceae	Ostropales	Lecanoromycetes	TLC → HPLC → MS → NMR	(Elix et al. 1995)
Rinodina alba	Physciaceae	Caliciales	Lecanoromycetes	TLC → HPLC → MS → NMR	(Elix et al. 1995)
**2"-*O*-methyltenuiorin**					
Pseudocyphellaria faveolata	Lobariaceae	Peltigerales	Lecanoromycetes	TLC → MS → NMR	(Elix and Lajide 1981)
**2',2"-di-*O*-methyltenuiorin**					
P. faveolata	Lobariaceae	Peltigerales	Lecanoromycetes	TLC → MS → NMR	(Elix and Lajide 1981)
**2,2'-di-*O*-methylgyrophoric acid**					
Evernia prunastri	Parmeliaceae	Lecanorales	Lecanoromycetes	HPLC	(González et al. 2002)
**2,4,5-tri-*O*-methylhiascic acid**					
Hypotrachyna neodamaziana	Parmeliaceae	Lecanorales	Lecanoromycetes	TLC → MS → NMR	(Elix et al. 1981)
H. schindleri	Parmeliaceae	Lecanorales	Lecanoromycetes	TLC → HPLC → MS	(Elix et al. 1991)
**2,4-di-*O*-methylgyrophoric acid**					
H. bonariensis	Parmeliaceae	Lecanorales	Lecanoromycetes	TLC	(Benatti 2012a)
H. neodamaziana	Parmeliaceae	Lecanorales	Lecanoromycetes	TLC → MS → NMR	(Elix et al. 1981)
H. schindleri	Parmeliaceae	Lecanorales	Lecanoromycetes	TLC → HPLC → MS	(Elix et al. 1991)
H. showmanii	Parmeliaceae	Lecanorales	Lecanoromycetes	TLC	(Lendemer and Allen 2020)
H. upretii	Parmeliaceae	Lecanorales	Lecanoromycetes	TLC	(Divakar and Crespo 2018)
Parmelinopsis horrescens	Parmeliaceae	Lecanorales	Lecanoromycetes	TLC	(Benatti 2012a)
P. subfatiscens	Parmeliaceae	Lecanorales	Lecanoromycetes	TLC	(Park 1990)
**2"-*O*-methylhiascic acid**					
Placopsis fusciduloides	Trapeliaceae	Baeomycetales	Lecanoromycetes	TLC → HPLC	(Galloway 2010)
**2-*O*-acetyltenuiorin**					
Peltigera didactyla	Peltigeraceae	Peltigerales	Lecanoromycetes	TLC	(Bryan and Elix 1976)
P. dolichorrhiza	Peltigeraceae	Peltigerales	Lecanoromycetes	TLC → MS → NMR	(Bryan and Elix 1976)
Pseudocyphellaria cinnamomea	Lobariaceae	Peltigerales	Lecanoromycetes	TLC	(Bryan and Elix 1976)
P. crocata	Lobariaceae	Peltigerales	Lecanoromycetes	TLC → MS → NMR	(Bryan and Elix 1976)
P. neglecta	Lobariaceae	Peltigerales	Lecanoromycetes	TLC → MS → NMR	(Bryan and Elix 1976)
**2-*O*-methylgyrophoric acid**					
Bulbothrix amazonensis	Parmeliaceae	Lecanorales	Lecanoromycetes	TLC	(Morales-Mendez et al. 1995)
Peltigeropsis vainioi	Peltigeraceae	Peltigerales	Lecanoromycetes	TLC	(Marcano et al. 1997)
**2'-*O*-Methylhiascic acid**					
Ramalina corymbosa	Ramalinaceae	Lecanorales	Lecanoromycetes	TLC → IR → MS → NMR	(Vinet et al. 1990)
**2-*O*-methylhiascic acid**					

Hypotrachyna neodamaziana	Parmeliaceae	Lecanorales	Lecanoromycetes	TLC → MS → NMR	(Elix et al. 1989a)
**2'-*O*-methyltenuiorin**					
Pseudocyphellaria faveolata	Lobariaceae	Peltigerales	Lecanoromycetes	TLC → MS → NMR	(Elix and Lajide 1981)
**3-hydroxygyrophoric acid**					
Xanthoparmelia fangii	Parmeliaceae	Lecanorales	Lecanoromycetes	TLC	(Elix 2006)
**3-hydroxyumbilicaric acid**					
Hypotrachyna bonariensis	Parmeliaceae	Lecanorales	Lecanoromycetes	TLC → HPLC → MS → NMR	(Elix et al. 1989b)
**3-methoxy-2,4-di-*O*-methylgyrophoric acid**					
H. bonariensis	Parmeliaceae	Lecanorales	Lecanoromycetes	TLC	(Benatti 2012a)
H. mcmulliniana	Parmeliaceae	Lecanorales	Lecanoromycetes	TLC	(Lendemer and Allen 2020)
H. neodamaziana	Parmeliaceae	Lecanorales	Lecanoromycetes	TLC	(Benatti 2012a)
H. schindleri	Parmeliaceae	Lecanorales	Lecanoromycetes	TLC → HPLC → MS	(Elix et al. 1991)
H. showmanii	Parmeliaceae	Lecanorales	Lecanoromycetes	TLC	(Lendemer and Allen 2020)
H. upretii	Parmeliaceae	Lecanorales	Lecanoromycetes	TLC	(Divakar and Crespo 2018)
Parmelinopsis horrescens	Parmeliaceae	Lecanorales	Lecanoromycetes	TLC	(Lendemer and Allen 2020)
P. minarum	Parmeliaceae	Lecanorales	Lecanoromycetes	TLC	(Eliasaro and Adler 2000)
P. subfatiscens	Parmeliaceae	Lecanorales	Lecanoromycetes	TLC → HPLC → MS → NMR	(Elix and Jayanthi 1981)
**3-methoxyumbilicaric acid**					
Hypotrachyna bonariensis	Parmeliaceae	Lecanorales	Lecanoromycetes	TLC → HPLC → MS → NMR	(Elix et al. 1989b)
**4,2"-*O*-methylgyrophoric acid**					
Evernia Prunastri	Parmeliaceae	Lecanorales	Lecanoromycetes	IR → NMR	(Nicollier et al. 1979)
**4,5-di-*O*-methylhiascic acid**					
Hypotrachyna appalachensis	Parmeliaceae	Lecanorales	Lecanoromycetes	TLC	(Lendemer and Allen 2020)
H. bonariensis	Parmeliaceae	Lecanorales	Lecanoromycetes	TLC	(Benatti 2012a)
H. Britannica	Parmeliaceae	Lecanorales	Lecanoromycetes	TLC	(Lendemer and Allen 2020)
H. cryptochlora	Parmeliaceae	Lecanorales	Lecanoromycetes	TLC	(Lendemer and Allen 2020)
H. kauffmaniana	Parmeliaceae	Lecanorales	Lecanoromycetes	TLC	(Lendemer and Allen 2020)
H. neodamaziana	Parmeliaceae	Lecanorales	Lecanoromycetes	TLC	(Benatti 2012a)
H. revoluta	Parmeliaceae	Lecanorales	Lecanoromycetes	TLC	(Lendemer and Allen 2020)
H. schindleri	Parmeliaceae	Lecanorales	Lecanoromycetes	TLC	(Benatti 2012a)
Ochrolechia africana	Ochrolechiaceae	Pertusariales	Lecanoromycetes	TLC	(Ren 2017)
O. alticola	Ochrolechiaceae	Pertusariales	Lecanoromycetes	TLC	(Ren 2017)
O. mexicana	Ochrolechiaceae	Pertusariales	Lecanoromycetes	TLC → HPLC	(Lumbsch et al. 2003)
Parmelia pseudofatiscens	Parmeliaceae	Lecanorales	Lecanoromycetes	TLC → MS → NMR	(Elix and Engkaninan 1976)
Parmelinopsis afrorevoluta	Parmeliaceae	Lecanorales	Lecanoromycetes	TLC	(Lendemer and Allen 2020)
P. horrescens	Parmeliaceae	Lecanorales	Lecanoromycetes	TLC	(Benatti 2012a)

P. minarum	Parmeliaceae	Lecanorales	Lecanoromycetes	TLC	(Lendemer and Allen 2020)
P. spumosa	Parmeliaceae	Lecanorales	Lecanoromycetes	TLC	(Lendemer and Allen 2020)
P. subfatiscens	Parmeliaceae	Lecanorales	Lecanoromycetes	TLC → HPLC → MS → NMR	(Elix and Jayanthi 1981)
**4-*O*-methylgyrophoric acid**					
Dendriscosticta platyphylla	Lobariaceae	Peltigerales	Lecanoromycetes	IR → MS → NMR	(Zhang et al. 2006)
Lobaria adscripturiens	Lobariaceae	Peltigerales	Lecanoromycetes	TLC	(Park 1990)
L. crassior	Lobariaceae	Peltigerales	Lecanoromycetes	TLC → HPLC	(Din et al. 1999)
L. linita	Lobariaceae	Peltigerales	Lecanoromycetes	TLC → NMR	(Maass 1975b)
L. tuberculata	Lobariaceae	Peltigerales	Lecanoromycetes	TLC	(Cornejo and Scheidegger 2015)
Lobariella angustata	Lobariaceae	Peltigerales	Lecanoromycetes	TLC	(Moncada et al. 2013)
L. flavomedullosa	Lobariaceae	Peltigerales	Lecanoromycetes	TLC	(Moncada et al. 2013)
L. isidiata	Lobariaceae	Peltigerales	Lecanoromycetes	TLC	(Moncada et al. 2013)
L. nashi	Lobariaceae	Peltigerales	Lecanoromycetes	TLC	(Moncada et al. 2013)
L. rugulosa	Lobariaceae	Peltigerales	Lecanoromycetes	TLC	(Moncada et al. 2013)
L. spathulifera	Lobariaceae	Peltigerales	Lecanoromycetes	TLC	(Moncada et al. 2013)
L. stenroosiae	Lobariaceae	Peltigerales	Lecanoromycetes	TLC	(Moncada et al. 2013)
L. subcrenulata	Lobariaceae	Peltigerales	Lecanoromycetes	TLC	(Moncada et al. 2013)
Nephroma gyelnikii	Nephromataceae	Peltigerales	Lecanoromycetes	TLC → MS	(Renner et al. 1982)
N. pseudoparile	Nephromataceae	Peltigerales	Lecanoromycetes	TLC → MS	(Renner et al. 1982)
Nipponoparmelia isidioclada	Parmeliaceae	Lecanorales	Lecanoromycetes	TLC	(Kondratyuk et al. 2013)
Peltigera aphthosa	Peltigeraceae	Peltigerales	Lecanoromycetes	TLC-→ IR → MS → NMR	(Maass 1975c)
P. cf. neglecta	Peltigeraceae	Peltigerales	Lecanoromycetes	TLC	(Samsudin et al. 1998)
P. polydactyla	Peltigeraceae	Peltigerales	Lecanoromycetes	TLC	(Samsudin et al. 1998)
Pseudocyphellaria crocata	Lobariaceae	Peltigerales	Lecanoromycetes	HPLC-DAD-MS	(Gadea et al. 2017)
P. quercifolia	Lobariaceae	Peltigerales	Lecanoromycetes	TLC	(Maass 1975)
P. sulphurea	Lobariaceae	Peltigerales	Lecanoromycetes	TLC	(Samsudin et al. 1998)
Yoshimuriella fendleri	Lobariaceae	Peltigerales	Lecanoromycetes	HPLC	(Stenroos et al. 2003)
**4-*O*-methylhiascic acid**					
Hypotrachyna schindleri	Parmeliaceae	Lecanorales	Lecanoromycetes	TLC → HPLC → MS	(Elix et al. 1991)
**5-*O*-methylhiascic acid**					
Aspiciliopsis macrophthalma	Trapeliaceae	Baeomycetales	Lecanoromycetes	TLC → HPLC	(Galloway 2010)
Australiaena streimannii	Caliciaceae	Caliciales	Lecanoromycetes	TLC	(Matzer et al. 1997)
Buellia eganii	Caliciaceae	Caliciales	Lecanoromycetes	HPLC	(Bungartz and Nash 2004)
B. gypsyensis	Caliciaceae	Caliciales	Lecanoromycetes	TLC	(Fryday 2019)
Hypotrachyna appalachensis	Parmeliaceae	Lecanorales	Lecanoromycetes	TLC	(Lendemer and Allen 2020)
H. bonariensis	Parmeliaceae	Lecanorales	Lecanoromycetes	TLC	(Benatti 2012a)

H. Britannica	Parmeliaceae	Lecanorales	Lecanoromycetes	TLC	(Lendemer and Allen 2020)
H. cryptochlora	Parmeliaceae	Lecanorales	Lecanoromycetes	TLC	(Lendemer and Allen 2020)
H. kauffmaniana	Parmeliaceae	Lecanorales	Lecanoromycetes	TLC	(Lendemer and Allen 2020)
H. mcmulliniana	Parmeliaceae	Lecanorales	Lecanoromycetes	TLC	(Lendemer and Allen 2020)
H. neodamaziana	Parmeliaceae	Lecanorales	Lecanoromycetes	TLC → MS → NMR	(Elix et al. 1981)
H. revoluta	Parmeliaceae	Lecanorales	Lecanoromycetes	TLC	(Lendemer and Allen 2020)
H. schindleri	Parmeliaceae	Lecanorales	Lecanoromycetes	TLC → HPLC → MS	(Elix et al. 1991)
H. upretii	Parmeliaceae	Lecanorales	Lecanoromycetes	TLC	(Divakar and Crespo 2018)
Micarea coppinsii	Pilocarpaceae	Lecanorales	Lecanoromycetes	TLC	(Urbanavichus and Urbanavichene 2018)
M. denigrate	Pilocarpaceae	Lecanorales	Lecanoromycetes	TLC	(Urbanavichus et al. 2020)
Ochrolechia africana	Ochrolechiaceae	Pertusariales	Lecanoromycetes	TLC	(Ren 2017)
Pannaria leproloma	Pannariaceae	Peltigerales	Lecanoromycetes	TLC → HPLC	(Galloway 2010)
Parmelia pseudofatiscens	Parmeliaceae	Lecanorales	Lecanoromycetes	NMR → synthesis	(Elix and Jayanthi 1977)
Parmelinopsis afrorevoluta	Parmeliaceae	Lecanorales	Lecanoromycetes	TLC	(Lendemer and Allen 2020)
P. horrescens	Parmeliaceae	Lecanorales	Lecanoromycetes	TLC	(Lendemer and Allen 2020)
P. minarum	Parmeliaceae	Lecanorales	Lecanoromycetes	TLC	(Lendemer and Allen 2020)
P. spumosa	Parmeliaceae	Lecanorales	Lecanoromycetes	TLC	(Lendemer and Allen 2020)
P. subfatiscens	Parmeliaceae	Lecanorales	Lecanoromycetes	TLC → HPLC → MS → NMR	(Elix and Jayanthi 1981)
Peltigeropsis vainioi	Peltigeraceae	Peltigerales	Lecanoromycetes	TLC	(Marcano et al. 1997)
Placopsis fusciduloides	Trapeliaceae	Baeomycetales	Lecanoromycetes	TLC → HPLC	(Galloway 2010)
P. gelidioides	Trapeliaceae	Baeomycetales	Lecanoromycetes	TLC → HPLC	(Galloway 2010)
P. lambii	Trapeliaceae	Baeomycetales	Lecanoromycetes	TLC	(Kukwa et al. 2012)
P. murrayi	Trapeliaceae	Baeomycetales	Lecanoromycetes	TLC	(Galloway 2004)
Placynthiella icmalea	Agyriaceae	Pertusariales	Lecanoromycetes	TLC	(Upreti et al. 2008)
Rinodina aspersa	Physciaceae	Caliciales	Lecanoromycetes	TLC	(Giralt et al. 1996)
Schaereria corticola	Schaereriaceae	Schaereriales	Lecanoromycetes	TLC	(Urbanavichus et al. 2020)
Stereocaulon caespitosum	Stereocaulaceae	Lecanorales	Lecanoromycetes	NMR	(Youn et al. 2018)
Trapelia calvariana	Trapeliaceae	Baeomycetales	Lecanoromycetes	TLC	(Kantvilas et al. 2014)
T. coarctata	Trapeliaceae	Baeomycetales	Lecanoromycetes	TLC	(Orange 2018)
T. collaris	Trapeliaceae	Baeomycetales	Lecanoromycetes	TLC	(Orange 2018)
T. corticola	Trapeliaceae	Baeomycetales	Lecanoromycetes	TLC	(Urbanavichene and Urbanavichus 2019)
T. elacista	Trapeliaceae	Baeomycetales	Lecanoromycetes	TLC	(Orange 2018)
T. glebulosa	Trapeliaceae	Baeomycetales	Lecanoromycetes	TLC	(Orange 2018)
T. involuta	Trapeliaceae	Baeomycetales	Lecanoromycetes	TLC	(Orange 2018)
T. lilacea	Trapeliaceae	Baeomycetales	Lecanoromycetes	TLC → HPLC	(Kantvilas and Elix 2007)

T. obtegens	Trapeliaceae	Baeomycetales	Lecanoromycetes	TLC	(Orange 2018)
T. sitiens	Trapeliaceae	Baeomycetales	Lecanoromycetes	TLC	(Orange 2018)
T. thieleana	Trapeliaceae	Baeomycetales	Lecanoromycetes	TLC	(Kantvilas et al. 2014)
**Crustinic acid**					
Diploschistes gyrophoricus	Graphidaceae	Ostropales	Lecanoromycetes	TLC → HPLC → MS → NMR	(Elix et al. 1995)
Umbilicaria cinereorufescens	Umbilicariaceae	Umbilicariales	Lecanoromycetes	TLC → HPLC → MS → NMR	(Narui et al. 1998)
U. crustulosa	Umbilicariaceae	Umbilicariales	Lecanoromycetes	TLC → HPLC	(Narui et al. 1996a)
**Deliseic acid**					
Cetrariella delisei	Parmeliaceae	Lecanorales	Lecanoromycetes	TLC → HPLC → MS → NMR	(Narui et al. 1998)
Koerberiella wimmeriana	Lecideaceae	Lecideales	Lecanoromycetes	TLC → HPLC → MS → NMR	(Elix et al. 1991)
**Depsidellin A**					
Xanthoparmelia depsidella	Parmeliaceae	Lecanorales	Lecanoromycetes	TLC → HPLC → MS → NMR	(Elix and Wardlaw 1997)
**Depsidellin B**					
X. depsidella	Parmeliaceae	Lecanorales	Lecanoromycetes	TLC → HPLC → MS → NMR	(Elix and Wardlaw 1997)
**Depsidellin C**					
X. depsidella	Parmeliaceae	Lecanorales	Lecanoromycetes	TLC → HPLC → MS → NMR	(Elix and Wardlaw 1997)
**Gyrophoric acid**					
Acarospora admissa	Acarosporaceae	Acarosporales	Lecanoromycetes	TLC	(Huneck and Schreiber 1974)
A. brucei	Acarosporaceae	Acarosporales	Lecanoromycetes	TLC	(Knudsen and Kocourková 2018)
A. bullata	Acarosporaceae	Acarosporales	Lecanoromycetes	Spot test	(Brinker and Knudsen 2019)
A. fulva	Acarosporaceae	Acarosporales	Lecanoromycetes	Spot test	(Nurtai et al. 2018)
A. fuscescens	Acarosporaceae	Acarosporales	Lecanoromycetes	Spot test	(Knudsen 2008)
A.nicolai	Acarosporaceae	Acarosporales	Lecanoromycetes	TLC	(Knudsen and Morse 2009)
A. pseudofuscata	Acarosporaceae	Acarosporales	Lecanoromycetes	TLC	(Sipman and Raus 2002)
A. ramosa	Acarosporaceae	Acarosporales	Lecanoromycetes	TLC	(Knudsen and Flakus 2009)
A. rosulata	Acarosporaceae	Acarosporales	Lecanoromycetes	TLC	(Knudsen et al. 2010)
A. tianshanica	Acarosporaceae	Acarosporales	Lecanoromycetes	Spot test	(Nurtai et al. 2017)
A. umbilicata	Acarosporaceae	Acarosporales	Lecanoromycetes	TLC	(Sipman and Raus 2002)
A. wahlenbergii	Acarosporaceae	Acarosporales	Lecanoromycetes	Spot test	(Timdal 1984)
Acroscyphus sphaerophoroides	Caliciaceae	Caliciales	Lecanoromycetes	TLC	(Niu et al. 2011a)
Ainoa geochroa	Baeomycetaceae	Baeomycetales	Lecanoromycetes	TLC	(Lumbsch et al. 2001)
Amygdalaria panaeola	Lecideaceae	Lecideales	Lecanoromycetes	TLC	(Gowan 1989)
A. pelobotryon	Lecideaceae	Lecideales	Lecanoromycetes	Spot test	(Fryday and Van Den Boom 2019)
A. subdissentien	Lecideaceae	Lecideales	Lecanoromycetes	TLC	(Gowan 1989)
Angiactis banksiae	Roccellaceae	Arthoniales	Arthoniomycetes	TLC → HPLC	(Kantvilas et al. 2020)
A. littoralis	Roccellaceae	Arthoniales	Arthoniomycetes	TLC	(Aptroot et al. 2008)

A. spinicola	Roccellaceae	Arthoniales	Arthoniomycetes	TLC	(Aptroot et al. 2008)
Anzina carneonivea	Agyriaceae	Pertusariales	Lecanoromycetes	TLC	(Lumbsch et al. 2001)
Arthonia anglica	Arthoniaceae	Arthoniales	Arthoniomycetes	TLC	(Frisch et al. 2014)
Aspicilia quartzitica	Hymeneliaceae	Hymeneliales	Lecanoromycetes	Spot test	(Weber 1971)
Aspiciliopsis macrophthalma	Trapeliaceae	Baeomycetales	Lecanoromycetes	TLC → HPLC	(Galloway 2010)
Australiaena streimannii	Caliciaceae	Caliciales	Lecanoromycetes	TLC	(Matzer et al. 1997)
Austromelanelixia subglabra	Parmeliaceae	Lecanorales	Lecanoromycetes	TLC	(Esslinger 1973)
Bellemerea cupreoatra	Lecideaceae	Lecideales	Lecanoromycetes	TLC	(Calatayud and Rambold 1998)
Biatora bacidioides	Ramalinaceae	Lecanorales	Lecanoromycetes	TLC	(Urbanavichus et al. 2020)
B. chrysantha	Ramalinaceae	Lecanorales	Lecanoromycetes	TLC	(Urbanavichus et al. 2020)
B. chrysanthoides	Ramalinaceae	Lecanorales	Lecanoromycetes	TLC	(Urbanavichus et al. 2020)
B. helvola	Ramalinaceae	Lecanorales	Lecanoromycetes	HPTLC	(Ekman 1994)
B. printzenii	Ramalinaceae	Lecanorales	Lecanoromycetes	TLC	(Tønsberg 2002)
B. vernalis	Ramalinaceae	Lecanorales	Lecanoromycetes	TLC	(Davydov and Printzen 2012)
Bryoria implexa	Parmeliaceae	Lecanorales	Lecanoromycetes	TLC	(Velmala et al. 2014)
B. pikei	Parmeliaceae	Lecanorales	Lecanoromycetes	TLC	(Velmala et al. 2014)
B. vrangiana	Parmeliaceae	Lecanorales	Lecanoromycetes	TLC	(Myllys et al. 2016)
Buellia gypsyensis	Caliciaceae	Caliciales	Lecanoromycetes	TLC	(Fryday 2019)
B. imshaugii	Caliciaceae	Caliciales	Lecanoromycetes	HPLC	(Giralt and Elix 2010)
B. lacteoidea	Caliciaceae	Caliciales	Lecanoromycetes	TLC	(Yakovchenko et al. 2021)
B. rhizocarpica	Caliciaceae	Caliciales	Lecanoromycetes	HPLC	(Etayo et al. 2010)
B. saxorum	Caliciaceae	Caliciales	Lecanoromycetes	Spot test	(Fryday and Van Den Boom 2019)
B. sequax	Caliciaceae	Caliciales	Lecanoromycetes	TLC → HPLC	(Giralt et al. 2011)
Bulbothricella amazonensis	Parmeliaceae	Lecanorales	Lecanoromycetes	TLC	(Marcano et al. 1996)
Bulbothrix amazonensis	Parmeliaceae	Lecanorales	Lecanoromycetes	TLC	(Morales-Mendez et al. 1995)
B. bulbillosa	Parmeliaceae	Lecanorales	Lecanoromycetes	TLC	(Benatti et al. 2015)
B. coronata	Parmeliaceae	Lecanorales	Lecanoromycetes	Spot test	(Culberson 1961)
B. fungicola	Parmeliaceae	Lecanorales	Lecanoromycetes	TLC	(Bungartz et al. 2013)
B. goebelii	Parmeliaceae	Lecanorales	Lecanoromycetes	TLC → HPLC	(Louwhoff and Elix 2000)
B. lacinulata	Parmeliaceae	Lecanorales	Lecanoromycetes	TLC → HPLC	(Jungbluth et al. 2008)
B. lobarica	Parmeliaceae	Lecanorales	Lecanoromycetes	TLC → HPLC	(Jungbluth et al. 2008)
B. papyrina	Parmeliaceae	Lecanorales	Lecanoromycetes	TLC → HPLC	(Benatti and Elix 2012)
B. pseudofungicola	Parmeliaceae	Lecanorales	Lecanoromycetes	TLC	(Benatti 2012b)
B. silicisrea	Parmeliaceae	Lecanorales	Lecanoromycetes	TLC	(Benatti 2012b)
B. suffixa	Parmeliaceae	Lecanorales	Lecanoromycetes	TLC	(Alstrup et al. 2010)
Byssoloma rubromarginatum	Pilocarpaceae	Lecanorales	Lecanoromycetes	HPTLC → HPLC	(Messuti and De La Rosa 2007)

Caloplaca gyrophorica	Teloschistaceae	Teloschistales	Lecanoromycetes	TLC	(Joshi et al. 2012)
C. teicholyta	Teloschistaceae	Teloschistales	Lecanoromycetes	Raman spectroscopy	(Miralles et al. 2015)
Canoparmelia martinicana	Parmeliaceae	Lecanorales	Lecanoromycetes	TLC → HPLC	(Canez et al. 2009)
C. martinicana	Parmeliaceae	Lecanorales	Lecanoromycetes	TLC	(Hale 1971)
Cetrariella delisei	Parmeliaceae	Lecanorales	Lecanoromycetes	TLC → HPLC → MS → NMR	(Narui et al. 1998)
Chiodecton Japonicum	Roccellaceae	Arthoniales	Arthoniomycetes	Spot test	(Culberson 1963)
Cladonia cervicornis	Cladoniaceae	Lecanorales	Lecanoromycetes	HPLC	(Din et al. 2010)
C. squamosa	Cladoniaceae	Lecanorales	Lecanoromycetes	TLC	(Kusmoro et al. 2018)
Crypthonia olivacea	Arthoniaceae	Arthoniales	Arthoniomycetes	HPTLC	(Frisch and Thor 2010)
Cryptothecia bartlettii	Arthoniaceae	Arthoniales	Arthoniomycetes	TLC	(Jagadeesh Ram and Sinha 2016)
C. culbersoniae	Arthoniaceae	Arthoniales	Arthoniomycetes	TLC	(Jagadeesh Ram and Sinha 2016)
C. dispersa	Arthoniaceae	Arthoniales	Arthoniomycetes	TLC	(Bajpai et al. 2017)
C. elongata	Arthoniaceae	Arthoniales	Arthoniomycetes	TLC	(Jagadeesh Ram and Sinha 2016)
C. emergens	Arthoniaceae	Arthoniales	Arthoniomycetes	TLC	(Jagadeesh Ram and Sinha 2016)
C. eungellae	Arthoniaceae	Arthoniales	Arthoniomycetes	TLC	(Jagadeesh Ram and Sinha 2016)
C. faveolata	Arthoniaceae	Arthoniales	Arthoniomycetes	TLC	(Bajpai et al. 2017)
C. faveomaculata	Arthoniaceae	Arthoniales	Arthoniomycetes	TLC → MS → NMR	(Ngoc Tuan et al. 2019)
C. inexpectata	Arthoniaceae	Arthoniales	Arthoniomycetes	TLC	(Jagadeesh Ram and Sinha 2016)
C. macrospora	Arthoniaceae	Arthoniales	Arthoniomycetes	TLC	(Jagadeesh Ram and Sinha 2016)
C. obtecta	Arthoniaceae	Arthoniales	Arthoniomycetes	TLC	(Jagadeesh Ram and Sinha 2016)
C. punctosorediata	Arthoniaceae	Arthoniales	Arthoniomycetes	TLC	(Sparrius and Saipunkaew 2005)
C. rosaeiselae	Arthoniaceae	Arthoniales	Arthoniomycetes	TLC	(Jagadeesh Ram and Sinha 2016)
C. scripta	Arthoniaceae	Arthoniales	Arthoniomycetes	TLC	(Bajpai et al. 2017)
C. subnidulans	Arthoniaceae	Arthoniales	Arthoniomycetes	TLC	(Yakovchenko et al. 2019)
Dactylina arctica	Parmeliaceae	Lecanorales	Lecanoromycetes	HPLC-DAD	(Stocker-Wörgötter and Elix 2006)
Dendriscosticta platyphylla	Lobariaceae	Peltigerales	Lecanoromycetes	TLC	(Rao et al. 1967)
Dimelaena mayrhoferiana	Caliciaceae	Caliciales	Lecanoromycetes	TLC	(Aptroot and Cáceres 2018)
D. oreina	Caliciaceae	Caliciales	Lecanoromycetes	TLC → HPLC	(Culberson et al. 1984)
D. subsquamulosa	Caliciaceae	Caliciales	Lecanoromycetes	TLC	(Giralt et al. 2014)
D. tenuis	Caliciaceae	Caliciales	Lecanoromycetes	TLC	(Upreti et al. 2010)
Diploschistes actinostomus	Graphidaceae	Ostropales	Lecanoromycetes	TLC	(Joshi et al. 2011)
D. badius	Graphidaceae	Ostropales	Lecanoromycetes	TLC	(Lumbsch and Elix 1989)
D. gyrophoricus	Graphidaceae	Ostropales	Lecanoromycetes	TLC → HPLC → MS → NMR	(Elix et al. 1995)
D. sticticus	Graphidaceae	Ostropales	Lecanoromycetes	TLC → HPLC	(Lumbsch and Elix 1989)
Dirinaria aegialita	Caliciaceae	Caliciales	Lecanoromycetes	Raman spectroscopy	(Miralles et al. 2015)
D. applanata	Caliciaceae	Caliciales	Lecanoromycetes	Raman spectroscopy	(Miralles et al. 2015)

Enterographa bradleyana	Roccellaceae	Arthoniales	Arthoniomycetes	TLC	(Seavey and Seavey 2014)
E. deslooveri	Roccellaceae	Arthoniales	Arthoniomycetes	TLC	(Seavey and Seavey 2014)
E. elaborata	Roccellaceae	Arthoniales	Arthoniomycetes	TLC	(Seavey and Seavey 2014)
E. glaucotremoides	Roccellaceae	Arthoniales	Arthoniomycetes	TLC	(Aptroot and Cáceres 2018)
E. lecanoracea	Roccellaceae	Arthoniales	Arthoniomycetes	TLC	(Seavey and Seavey 2014)
E. leucolyta	Roccellaceae	Arthoniales	Arthoniomycetes	TLC	(Yakovchenko et al. 2019)
E. murrayana	Roccellaceae	Arthoniales	Arthoniomycetes	TLC	(Seavey and Seavey 2014)
E. oregonensis	Roccellaceae	Arthoniales	Arthoniomycetes	TLC	(Sparrius and Björk 2008)
E. pallidella	Roccellaceae	Arthoniales	Arthoniomycetes	TLC	(Seavey and Seavey 2014)
E. zephyri	Roccellaceae	Arthoniales	Arthoniomycetes	TLC	(Cáceres et al. 2014)
Fellhanera gyrophorica	Pilocarpaceae	Lecanorales	Lecanoromycetes	TLC	(Sérusiaux et al. 2001)
Flavoparmelia baltimorensis	Parmeliaceae	Lecanorales	Lecanoromycetes	TLC	(Culberson and Culberson 1982)
F. caperata	Parmeliaceae	Lecanorales	Lecanoromycetes	TLC	(Culberson and Culberson 1982)
Gyrographa gyrocarpa	Roccellaceae	Arthoniales	Arthoniomycetes	TLC	(Schiefelbein et al. 2014)
Haematomma persoonii	Haematommataceae	Lecanorales	Lecanoromycetes	TLC	(Kusmoro et al. 2018)
Herpothallon biacidum	Arthoniaceae	Arthoniales	Arthoniomycetes	HPTLC → HPLC	(Frisch et al. 2010)
H. fertile	Arthoniaceae	Arthoniales	Arthoniomycetes	TLC	(Frisch et al. 2014)
H. himalayanum	Arthoniaceae	Arthoniales	Arthoniomycetes	TLC	(Jagadeesh Ram and Sinha 2009)
H. japonicum	Arthoniaceae	Arthoniales	Arthoniomycetes	TLC	(Frisch et al. 2014)
H. minutum	Arthoniaceae	Arthoniales	Arthoniomycetes	TLC	(Jagadeesh Ram 2014)
H. philippinum	Arthoniaceae	Arthoniales	Arthoniomycetes	TLC	(Jagadeesh Ram 2014)
H. queenslandicum	Arthoniaceae	Arthoniales	Arthoniomycetes	TLC	(Frisch et al. 2014)
H. rubrocinctoides	Arthoniaceae	Arthoniales	Arthoniomycetes	TLC	(Frisch et al. 2014)
Hypotrachyna appalachensis	Parmeliaceae	Lecanorales	Lecanoromycetes	TLC	(Lendemer and Allen 2020)
H. bonariensis	Parmeliaceae	Lecanorales	Lecanoromycetes	TLC	(Benatti 2012a)
H. Britannica	Parmeliaceae	Lecanorales	Lecanoromycetes	TLC	(Lendemer and Allen 2020)
H. catawbiense	Parmeliaceae	Lecanorales	Lecanoromycetes	TLC	(Culberson and Culberson 1981)
H. cryptochlora	Parmeliaceae	Lecanorales	Lecanoromycetes	TLC	(Lendemer and Allen 2020)
H. ectypa	Parmeliaceae	Lecanorales	Lecanoromycetes	TLC	(Benatti 2012a)
H. kauffmaniana	Parmeliaceae	Lecanorales	Lecanoromycetes	TLC	(Lendemer and Allen 2020)
H. mcmulliniana	Parmeliaceae	Lecanorales	Lecanoromycetes	TLC	(Lendemer and Allen 2020)
H. megadactyla	Parmeliaceae	Lecanorales	Lecanoromycetes	TLC	(Benatti 2012a)
H. neodamaziana	Parmeliaceae	Lecanorales	Lecanoromycetes	TLC → MS → NMR	(Elix et al. 1981)
H. neodissecta	Parmeliaceae	Lecanorales	Lecanoromycetes	TLC	(Alstrup et al. 2010)
H. nigrociliata	Parmeliaceae	Lecanorales	Lecanoromycetes	TLC	(Culberson and Culberson 1981)
H. nodakensis	Parmeliaceae	Lecanorales	Lecanoromycetes	TLC	(Jayalal et al. 2013)

H. oostingii	Parmeliaceae	Lecanorales	Lecanoromycetes	TLC	(Dey 1978)
H. pindarensis	Parmeliaceae	Lecanorales	Lecanoromycetes	TLC	(Benatti 2012a)
H. revoluta	Parmeliaceae	Lecanorales	Lecanoromycetes	TLC	(Lendemer and Allen 2020)
H. schindleri	Parmeliaceae	Lecanorales	Lecanoromycetes	TLC → HPLC → MS	(Elix et al. 1991)
H. showmanii	Parmeliaceae	Lecanorales	Lecanoromycetes	TLC	(Lendemer and Allen 2020)
H. spathulata	Parmeliaceae	Lecanorales	Lecanoromycetes	TLC	(Benatti 2012a)
H. thysanota	Parmeliaceae	Lecanorales	Lecanoromycetes	TLC	(Dey 1978)
H. upretii	Parmeliaceae	Lecanorales	Lecanoromycetes	TLC	(Divakar and Crespo 2018)
Immersaria cf. olivacea	Lecideaceae	Lecideales	Lecanoromycetes	TLC	(Pandit 2014)
I. usbekica	Lecideaceae	Lecideales	Lecanoromycetes	TLC	(Calatayud and Rambold 1998)
Jasonhuria bogilana	Teloschistaceae	Teloschistales	Lecanoromycetes	TLC	(Joshi et al. 2010)
J. bogilana	Teloschistaceae	Teloschistales	Lecanoromycetes	TLC	(Kondratyuk et al. 2015)
Lambiella furvella	Xylographaceae	Baeomycetales	Lecanoromycetes	TLC	(Nordin 2004)
Lasallia papulosa	Umbilicariaceae	Umbilicariales	Lecanoromycetes	TLC → HPLC → MS → NMR	(Narui et al. 1998)
L. pustulata	Umbilicariaceae	Umbilicariales	Lecanoromycetes	TLC	(Rao et al. 1967)
Lecanactis rubra	Roccellaceae	Arthoniales	Arthoniomycetes	TLC	(Ertz and Sérusiaux 2009)
Lecanographa dimelaenoides	Lecanographaceae	Arthoniales	Arthoniomycetes	HPLC	(Egea et al. 1992)
L. hypothallina	Lecanographaceae	Arthoniales	Arthoniomycetes	HPLC	(Egea et al. 1992)
L. werneri	Lecanographaceae	Arthoniales	Arthoniomycetes	TLC → HPLC	(Egea et al. 1993)
Lecanora microloba	Lecanoraceae	Lecanorales	Lecanoromycetes	TLC	(Śliwa and Flakus 2011)
L. munzii	Lecanoraceae	Lecanorales	Lecanoromycetes	TLC	(Knudsen and Lendemer 2009)
L. peninsularis	Lecanoraceae	Lecanorales	Lecanoromycetes	TLC → HPLC	(Knudsen et al. 2011)
L. psarophana	Lecanoraceae	Lecanorales	Lecanoromycetes	TLC	(Sipman and Raus 2002)
Lecidea fuscoatra	Lecideaceae	Lecideales	Lecanoromycetes	Raman spectroscopy	(Miralles et al. 2015)
L. grisella	Lecideaceae	Lecideales	Lecanoromycetes	Spot test	(Fryday and Van Den Boom 2019)
L. gypsicola	Lecideaceae	Lecideales	Lecanoromycetes	TLC	(Casares-Porcel et al. 1996)
L. sciatropha	Lecideaceae	Lecideales	Lecanoromycetes	Raman spectroscopy	(Miralles et al. 2015)
L. uniformis	Lecideaceae	Lecideales	Lecanoromycetes	TLC	(McCune et al. 2017)
Lepraria cacuminum	Stereocaulaceae	Lecanorales	Lecanoromycetes	HPLC	(Knudsen et al. 2006)
L. maderensis	Stereocaulaceae	Lecanorales	Lecanoromycetes	TLC	(Flakus and Kukwa 2011)
Lethariella cashmeriana	Parmeliaceae	Lecanorales	Lecanoromycetes	TLC	(Obermayer 2001)
L. cladonioides	Parmeliaceae	Lecanorales	Lecanoromycetes	TLC → HPLC	(Niu et al. 2011b)
L. sernanderi	Parmeliaceae	Lecanorales	Lecanoromycetes	TLC	(Obermayer 2001)
L. sinensis	Parmeliaceae	Lecanorales	Lecanoromycetes	TLC	(Obermayer 2001)
L. zahlbruckneri	Parmeliaceae	Lecanorales	Lecanoromycetes	TLC	(Obermayer 2001)
Lithographa marionensis	Trapeliaceae	Baeomycetales	Lecanoromycetes	TLC	(Coppins and Fryday 2006)

Lobaria adscripturiens	Lobariaceae	Peltigerales	Lecanoromycetes	TLC	(Ren et al. 2012)
L. clemensiae	Lobariaceae	Peltigerales	Lecanoromycetes	TLC	(Gruezo 1983)
L. discolor	Lobariaceae	Peltigerales	Lecanoromycetes	TLC → HPLC	(Din et al. 1999)
L. fuscotomentosa	Lobariaceae	Peltigerales	Lecanoromycetes	TLC	(Pandit and Sharma 2012)
L. immixta	Lobariaceae	Peltigerales	Lecanoromycetes	TLC	(Cornejo and Scheidegger 2015)
L. insularis	Lobariaceae	Peltigerales	Lecanoromycetes	TLC	(Samsudin et al. 1998)
L. isidiophora	Lobariaceae	Peltigerales	Lecanoromycetes	TLC → HPLC	(Din et al. 1999)
L. isidiosa	Lobariaceae	Peltigerales	Lecanoromycetes	TLC	(Ren et al. 2012)
L. linita	Lobariaceae	Peltigerales	Lecanoromycetes	TLC → NMR	(Maass 1975b)
L. orientalis	Lobariaceae	Peltigerales	Lecanoromycetes	TLC	(Cornejo and Scheidegger 2015)
L. pindarensis	Lobariaceae	Peltigerales	Lecanoromycetes	TLC	(Cornejo and Scheidegger 2015)
L. ravenelii	Lobariaceae	Peltigerales	Lecanoromycetes	TLC	(Jordan 1972)
L. spathulata	Lobariaceae	Peltigerales	Lecanoromycetes	TLC	(Cornejo and Scheidegger 2015)
L. tuberculata	Lobariaceae	Peltigerales	Lecanoromycetes	TLC	(Cornejo and Scheidegger 2015)
L. yunnanensis	Lobariaceae	Peltigerales	Lecanoromycetes	Spectral techniques	(Zhang et al. 2008)
Lobariella angustata	Lobariaceae	Peltigerales	Lecanoromycetes	TLC	(Moncada et al. 2013)
L. crenulata	Lobariaceae	Peltigerales	Lecanoromycetes	TLC	(Moncada et al. 2013)
L. exornata	Lobariaceae	Peltigerales	Lecanoromycetes	TLC	(Moncada et al. 2013)
L. flavomedullosa	Lobariaceae	Peltigerales	Lecanoromycetes	TLC	(Moncada et al. 2013)
L. isidiata	Lobariaceae	Peltigerales	Lecanoromycetes	TLC	(Moncada et al. 2013)
L. nashi	Lobariaceae	Peltigerales	Lecanoromycetes	TLC	(Moncada et al. 2013)
L. pallida	Lobariaceae	Peltigerales	Lecanoromycetes	TLC	(Moncada et al. 2013)
L. pallidocrenulata	Lobariaceae	Peltigerales	Lecanoromycetes	TLC	(Moncada et al. 2013)
L. rugulosa	Lobariaceae	Peltigerales	Lecanoromycetes	TLC	(Moncada et al. 2013)
L. sipmanii	Lobariaceae	Peltigerales	Lecanoromycetes	TLC	(Moncada et al. 2013)
L. soredians	Lobariaceae	Peltigerales	Lecanoromycetes	TLC	(Moncada et al. 2013)
L. spathulifera	Lobariaceae	Peltigerales	Lecanoromycetes	TLC	(Moncada et al. 2013)
L. stenroosiae	Lobariaceae	Peltigerales	Lecanoromycetes	TLC	(Moncada et al. 2013)
L. subcrenulata	Lobariaceae	Peltigerales	Lecanoromycetes	TLC	(Moncada et al. 2013)
Micarea bebourensis	Pilocarpaceae	Lecanorales	Lecanoromycetes	Spot test	(Brand et al. 2014)
M. borbonica	Pilocarpaceae	Lecanorales	Lecanoromycetes	Spot test	(Brand et al. 2014)
M. boryana	Pilocarpaceae	Lecanorales	Lecanoromycetes	Spot test	(Brand et al. 2014)
M. cilaoensis	Pilocarpaceae	Lecanorales	Lecanoromycetes	Spot test	(Brand et al. 2014)
M. cinerea	Pilocarpaceae	Lecanorales	Lecanoromycetes	Spot test	(Brand et al. 2014)
M. coppinsii	Pilocarpaceae	Lecanorales	Lecanoromycetes	TLC	(Urbanavichus and Urbanavichene 2018)

M. denigrata	Pilocarpaceae	Lecanorales	Lecanoromycetes	TLC → HPLC	(Kantvilas and Coppins 2019)
M. globulosella	Pilocarpaceae	Lecanorales	Lecanoromycetes	TLC	(Urbanavichus et al. 2020)
M. isidiosa	Pilocarpaceae	Lecanorales	Lecanoromycetes	Spot test	(Brand et al. 2014)
M. kartana	Pilocarpaceae	Lecanorales	Lecanoromycetes	TLC	(Kantvilas 2018)
M. levicula	Pilocarpaceae	Lecanorales	Lecanoromycetes	Spot test	(Brand et al. 2014)
M. melaena	Pilocarpaceae	Lecanorales	Lecanoromycetes	TLC → HPLC	(Kantvilas and Coppins 2019)
M. mutabilis	Pilocarpaceae	Lecanorales	Lecanoromycetes	TLC → HPLC	(Kantvilas and Coppins 2019)
M. oreina	Pilocarpaceae	Lecanorales	Lecanoromycetes	TLC → HPLC	(Kantvilas and Coppins 2019)
M. peliocarpa	Pilocarpaceae	Lecanorales	Lecanoromycetes	Spot test	(Brand et al. 2014)
M. prasinastra	Pilocarpaceae	Lecanorales	Lecanoromycetes	TLC → HPLC	(Kantvilas and Coppins 2019)
M. prasinella	Pilocarpaceae	Lecanorales	Lecanoromycetes	TLC → HPLC	(Kantvilas and Coppins 2019)
M. pseudocoppinsii	Pilocarpaceae	Lecanorales	Lecanoromycetes	TLC → HPLC	(Kantvilas and Coppins 2019)
M. pycnidiophora	Pilocarpaceae	Lecanorales	Lecanoromycetes	TLC	(Czarnota 2015)
M. sambuci	Pilocarpaceae	Lecanorales	Lecanoromycetes	TLC	(Van Den Boom et al. 2018)
M. sipmanii	Pilocarpaceae	Lecanorales	Lecanoromycetes	TLC	(Serusiaux and Coppins 2009)
M. subcinerea	Pilocarpaceae	Lecanorales	Lecanoromycetes	TLC	(van den Boom 2004)
M. tenuispora	Pilocarpaceae	Lecanorales	Lecanoromycetes	Spot test	(Brand et al. 2014)
M. usneae	Pilocarpaceae	Lecanorales	Lecanoromycetes	Microcrystal test	(Van Den Boom and Ertz 2014)
M. viridileprosa	Pilocarpaceae	Lecanorales	Lecanoromycetes	TLC → HPLC	(Kantvilas and Coppins 2019)
Mikhtomia subflavorubescens	Teloschistaceae	Teloschistales	Lecanoromycetes	TLC	(Joshi et al. 2010)
Mischoblastia oxydata	Physciaceae	Caliciales	Lecanoromycetes	TLC	(Sipman 2010)
Mycoporum awasthii	Mycoporaceae	Pleosporales	Dothideomycetes	TLC	(Jagadeesh Ram and Sinha 2016)
Myriolecis congesta	Lecanoraceae	Lecanorales	Lecanoromycetes	TLC	(Sipman and Raus 2015)
Myrionora vacciniicola	Lecanoraceae	Lecanorales	Lecanoromycetes	TLC	(Urbanavichus et al. 2020)
Neobrownliella brownlieae	Teloschistaceae	Teloschistales	Lecanoromycetes	Spot test	(Kantvilas 2016)
Nephroma gyelnikii	Nephromataceae	Peltigerales	Lecanoromycetes	TLC → MS	(Renner et al. 1982)
N. pseudoparile	Nephromataceae	Peltigerales	Lecanoromycetes	TLC → MS	(Renner et al. 1982)
Nephromopsis cucullata	Parmeliaceae	Lecanorales	Lecanoromycetes	TLC → HPLC → FT-IR → MS	(Prokopiev et al. 2018)
Neuropogon sphacelatus	Parmeliaceae	Lecanorales	Lecanoromycetes	TLC → HPLC	(Elix et al. 2007)
Nipponoparmelia isidioclada	Parmeliaceae	Lecanorales	Lecanoromycetes	TLC	(Kondratyuk et al. 2013)
Ochrolechia aegaea	Ochrolechiaceae	Pertusariales	Lecanoromycetes	TLC	(Kukwa 2009)
O. africana	Ochrolechiaceae	Pertusariales	Lecanoromycetes	TLC	(Ren 2017)
O. akagiensis	Ochrolechiaceae	Pertusariales	Lecanoromycetes	TLC	(Park et al. 2019)
O. alaskana	Ochrolechiaceae	Pertusariales	Lecanoromycetes	TLC	(Kukwa 2009)
O. alboflavescens	Ochrolechiaceae	Pertusariales	Lecanoromycetes	TLC	(Stepanchikova et al. 2010)
O. alticola	Ochrolechiaceae	Pertusariales	Lecanoromycetes	TLC	(Ren 2017)

O. androgyna	Ochrolechiaceae	Pertusariales	Lecanoromycetes	TLC	(Ren 2017)
O. arborea	Ochrolechiaceae	Pertusariales	Lecanoromycetes	TLC	(Ren 2017)
O. balcanica	Ochrolechiaceae	Pertusariales	Lecanoromycetes	TLC	(Sipman and Raus 2015)
O. brodoi	Ochrolechiaceae	Pertusariales	Lecanoromycetes	TLC	(Brodo and McCune 2017)
O. dalmatica	Ochrolechiaceae	Pertusariales	Lecanoromycetes	TLC	(Sipman and Raus 2015)
O. frigida	Ochrolechiaceae	Pertusariales	Lecanoromycetes	TLC	(Ren 2017)
O. glacialis	Ochrolechiaceae	Pertusariales	Lecanoromycetes	TLC	(Ren 2017)
O. inaequatula	Ochrolechiaceae	Pertusariales	Lecanoromycetes	TLC	(Fryday and Coppins 1997)
O. isidiata	Ochrolechiaceae	Pertusariales	Lecanoromycetes	TLC → HPLC	(Lumbsch et al. 2003)
O. kerguelensis	Ochrolechiaceae	Pertusariales	Lecanoromycetes	TLC	(Ertz et al. 2016)
O. laevigata	Ochrolechiaceae	Pertusariales	Lecanoromycetes	TLC	(Brodo 1984)
O. lijiangensis	Ochrolechiaceae	Pertusariales	Lecanoromycetes	TLC	(Ren 2017)
O. longispora	Ochrolechiaceae	Pertusariales	Lecanoromycetes	TLC	(Ren 2017)
O. mahluensis	Ochrolechiaceae	Pertusariales	Lecanoromycetes	TLC	(Ren 2017)
O. margarita	Ochrolechiaceae	Pertusariales	Lecanoromycetes	TLC	(Ren 2017)
O. mexicana	Ochrolechiaceae	Pertusariales	Lecanoromycetes	TLC → HPLC	(Lumbsch et al. 2003)
O. pallentiisidiata	Ochrolechiaceae	Pertusariales	Lecanoromycetes	TLC	(Ren 2017)
O. pallescens	Ochrolechiaceae	Pertusariales	Lecanoromycetes	TLC	(Schmitt et al. 2008)
O. parella	Ochrolechiaceae	Pertusariales	Lecanoromycetes	TLC	(Ovstedal 1988)
O. parellula	Ochrolechiaceae	Pertusariales	Lecanoromycetes	TLC	(Park et al. 2019)
O. Peruensis	Ochrolechiaceae	Pertusariales	Lecanoromycetes	TLC → HPLC	(Lumbsch et al. 2003)
O. rugomarginata	Ochrolechiaceae	Pertusariales	Lecanoromycetes	TLC	(Ren 2017)
O. splendens	Ochrolechiaceae	Pertusariales	Lecanoromycetes	TLC → HPLC	(Lumbsch et al. 2003)
O. subrosella	Ochrolechiaceae	Pertusariales	Lecanoromycetes	TLC	(Ren 2017)
O. subviridis	Ochrolechiaceae	Pertusariales	Lecanoromycetes	TLC	(Schiefelbein et al. 2014)
O. tartarea	Ochrolechiaceae	Pertusariales	Lecanoromycetes	TLC	(Bjelland and Thorseth 2002)
O. trochophora	Ochrolechiaceae	Pertusariales	Lecanoromycetes	TLC	(Park et al. 2019)
O. yasudae	Ochrolechiaceae	Pertusariales	Lecanoromycetes	TLC	(Ren 2017)
Opegrapha gyrophorica	Opegraphaceae	Arthoniales	Arthoniomycetes	TLC	(Seavey et al. 2014)
O. keyensis	Opegraphaceae	Arthoniales	Arthoniomycetes	TLC	(Seavey et al. 2014)
O. ramisorediata	Opegraphaceae	Arthoniales	Arthoniomycetes	TLC	(Aptroot and Cáceres 2017)
Pannaria leproloma	Pannariaceae	Peltigerales	Lecanoromycetes	TLC → HPLC	(Galloway 2010)
Parmelia dubia	Parmeliaceae	Lecanorales	Lecanoromycetes	Spot test	(Culberson and Culberson 1956)
P. minandairana	Parmeliaceae	Lecanorales	Lecanoromycetes	Physicochemical	(Rangaswami and Rao 1954)
P. pseudofatiscens	Parmeliaceae	Lecanorales	Lecanoromycetes	TLC → MS → NMR	(Elix and Engkaninan 1976)
P. shinanoana	Parmeliaceae	Lecanorales	Lecanoromycetes	TLC	(Park 1990)

Parmelina atricha	Parmeliaceae	Lecanorales	Lecanoromycetes	TLC	(Clerc and Truong 2008)
P. quercina	Parmeliaceae	Lecanorales	Lecanoromycetes	TLC	(Clerc and Truong 2008)
Parmelinopsis afrorevoluta	Parmeliaceae	Lecanorales	Lecanoromycetes	TLC	(Lendemer and Allen 2020)
P. horrescens	Parmeliaceae	Lecanorales	Lecanoromycetes	TLC	(Lendemer and Allen 2020)
P. minarum	Parmeliaceae	Lecanorales	Lecanoromycetes	TLC	(Lendemer and Allen 2020)
P. spumosa	Parmeliaceae	Lecanorales	Lecanoromycetes	TLC	(Lendemer and Allen 2020)
P. subfatiscens	Parmeliaceae	Lecanorales	Lecanoromycetes	TLC → HPLC → MS → NMR	(Elix and Jayanthi 1981)
P. subinflata	Parmeliaceae	Lecanorales	Lecanoromycetes	TLC	(Benatti 2012a)
Parmotrema catarinae	Parmeliaceae	Lecanorales	Lecanoromycetes	TLC	(Eliasaro and Donha 2003)
P. clavuliferum	Parmeliaceae	Lecanorales	Lecanoromycetes	TLC	(Ahn and Moon 2016)
P. endosulphureum	Parmeliaceae	Lecanorales	Lecanoromycetes	TLC → HPLC	(Louwhoff and Elix 2000)
P. flavomedullosum	Parmeliaceae	Lecanorales	Lecanoromycetes	TLC	(Eliasaro and Donha 2003)
P. madagascariaceum	Parmeliaceae	Lecanorales	Lecanoromycetes	TLC	(Almeda and Dey 1973)
P. melanochaetum	Parmeliaceae	Lecanorales	Lecanoromycetes	TLC	(Benatti 2012a)
P. permutatum	Parmeliaceae	Lecanorales	Lecanoromycetes	TLC	(Eliasaro and Donha 2003)
P. pseudocrinitum	Parmeliaceae	Lecanorales	Lecanoromycetes	TLC	(Biju et al. 2010)
P. reticulatum	Parmeliaceae	Lecanorales	Lecanoromycetes	HPLC	(Din et al. 2010)
P. sancti-angelii	Parmeliaceae	Lecanorales	Lecanoromycetes	TLC	(Eliasaro and Donha 2003)
P. subschimperi	Parmeliaceae	Lecanorales	Lecanoromycetes	TLC	(Alstrup et al. 2010)
P. upretii	Parmeliaceae	Lecanorales	Lecanoromycetes	TLC	(Divakar and Upreti 2003)
P. vartakii	Parmeliaceae	Lecanorales	Lecanoromycetes	TLC	(Bawingan et al. 2017)
P. xanthinum	Parmeliaceae	Lecanorales	Lecanoromycetes	TLC	(Eliasaro and Donha 2003)
Peltigera aphthosa	Peltigeraceae	Peltigerales	Lecanoromycetes	TLC-→ IR → MS → NMR	(Maass 1975c)
P. britannica	Peltigeraceae	Peltigerales	Lecanoromycetes	TLC	(Goward et al. 1995)
P. cf. neglecta	Peltigeraceae	Peltigerales	Lecanoromycetes	TLC	(Samsudin et al. 1998)
P. cichoracea	Peltigeraceae	Peltigerales	Lecanoromycetes	TLC	(Sérusiaux and Goffinet 2009)
P. collina	Peltigeraceae	Peltigerales	Lecanoromycetes	TLC	(Wei et al. 2009)
P. didactyla	Peltigeraceae	Peltigerales	Lecanoromycetes	TLC	(Goffinet and Hastings 1995)
P. dissecta	Peltigeraceae	Peltigerales	Lecanoromycetes	TLC	(Purvis and James 1993)
P. dolichorhiza	Peltigeraceae	Peltigerales	Lecanoromycetes	TLC	(Sérusiaux and Goffinet 2009)
P. elisabethae	Peltigeraceae	Peltigerales	Lecanoromycetes	TLC	(Goffinet and Miadlikowska 1999)
P. extenuata	Peltigeraceae	Peltigerales	Lecanoromycetes	TLC	(Sérusiaux and Goffinet 2009)
P. hymenina	Peltigeraceae	Peltigerales	Lecanoromycetes	TLC	(Goward et al. 1995)
P. lambinonii	Peltigeraceae	Peltigerales	Lecanoromycetes	TLC	(Goffinet and Hastings 1995)
P. leucophlebia	Peltigeraceae	Peltigerales	Lecanoromycetes	TLC	(Goward et al. 1995)
P. malacea	Peltigeraceae	Peltigerales	Lecanoromycetes	TLC	(Goward et al. 1995)

P. melanorrhiza	Peltigeraceae	Peltigerales	Lecanoromycetes	TLC	(Purvis and James 1993)
P. nana	Peltigeraceae	Peltigerales	Lecanoromycetes	TLC	(Sérusiaux and Goffinet 2009)
P. neckeri	Peltigeraceae	Peltigerales	Lecanoromycetes	TLC	(Goward et al. 1995)
P. neopolydactyla	Peltigeraceae	Peltigerales	Lecanoromycetes	TLC	(Goward et al. 1995)
P. occidentalis	Peltigeraceae	Peltigerales	Lecanoromycetes	TLC	(Goward et al. 1995)
P. pacijca	Peltigeraceae	Peltigerales	Lecanoromycetes	TLC	(Goward et al. 1995)
P. phyllidiosa	Peltigeraceae	Peltigerales	Lecanoromycetes	TLC	(Goffinet and Miadlikowska 1999)
P. polydacrylon	Peltigeraceae	Peltigerales	Lecanoromycetes	TLC	(Goward et al. 1995)
P. polydactyla	Peltigeraceae	Peltigerales	Lecanoromycetes	TLC	(Samsudin et al. 1998)
P. retifoveata	Peltigeraceae	Peltigerales	Lecanoromycetes	TLC	(Goward et al. 1995)
P. scabrosa	Peltigeraceae	Peltigerales	Lecanoromycetes	TLC	(Goward et al. 1995)
P. tartarea	Peltigeraceae	Peltigerales	Lecanoromycetes	TLC	(Vitikainen 2006)
P. ulcerata	Peltigeraceae	Peltigerales	Lecanoromycetes	TLC	(Sérusiaux and Goffinet 2009)
P. venosa	Peltigeraceae	Peltigerales	Lecanoromycetes	TLC	(Goward et al. 1995)
Peltigeropsis vainioi	Peltigeraceae	Peltigerales	Lecanoromycetes	TLC	(Marcano et al. 1997)
Pertusaria Carneopallida	Pertusariaceae	Pertusariales	Lecanoromycetes	TLC	(Zhao et al. 2004)
P. conglobata	Pertusariaceae	Pertusariales	Lecanoromycetes	TLC	(Sipman and Raus 2015)
P. dispar	Pertusariaceae	Pertusariales	Lecanoromycetes	TLC	(Boqueras and Llimona 2003)
P. glaucomela	Pertusariaceae	Pertusariales	Lecanoromycetes	TLC	(Zhao et al. 2014)
P. hymenea	Pertusariaceae	Pertusariales	Lecanoromycetes	TLC	(Lumbsch et al. 1999)
P. montana	Pertusariaceae	Pertusariales	Lecanoromycetes	TLC	(Zhang and Ren 2016)
P. oculata	Pertusariaceae	Pertusariales	Lecanoromycetes	TLC	(Zhao et al. 2004)
P. pentelici	Pertusariaceae	Pertusariales	Lecanoromycetes	TLC	(Sipman and Raus 2002)
P. pseudoparotica	Pertusariaceae	Pertusariales	Lecanoromycetes	TLC	(Sipman and Raus 2002)
P. rubefacta	Pertusariaceae	Pertusariales	Lecanoromycetes	TLC	(Lumbsch et al. 1999)
Placopsis auriculata	Trapeliaceae	Baeomycetales	Lecanoromycetes	TLC → HPLC	(Lumbsch et al. 1993)
P. fusciduloides	Trapeliaceae	Baeomycetales	Lecanoromycetes	TLC → HPLC	(Galloway 2010)
P. gelida	Trapeliaceae	Baeomycetales	Lecanoromycetes	TLC	(Eigler and Poelt 1965)
P. gelidioides	Trapeliaceae	Baeomycetales	Lecanoromycetes	TLC → HPLC	(Galloway 2010)
P. lambii	Trapeliaceae	Baeomycetales	Lecanoromycetes	TLC	(Kukwa et al. 2012)
P. macrospora	Trapeliaceae	Baeomycetales	Lecanoromycetes	TLC	(Galloway 2004)
P. murrayi	Trapeliaceae	Baeomycetales	Lecanoromycetes	TLC	(Galloway 2004)
P. parellina	Trapeliaceae	Baeomycetales	Lecanoromycetes	TLC	(Huneck 1974)
P. polycarpa	Trapeliaceae	Baeomycetales	Lecanoromycetes	TLC	(Galloway 2004)
P. subparellina	Trapeliaceae	Baeomycetales	Lecanoromycetes	TLC	(Lumbsch et al. 2001)
P. venosa	Trapeliaceae	Baeomycetales	Lecanoromycetes	TLC	(Galloway 2004)

Placopsis perrugasa	Trapeliaceae	Baeomycetales	Lecanoromycetes	TLC	(Renner 1982)
Placynthiella dasaea	Agyriaceae	Pertusariales	Lecanoromycetes	TLC	(Tønsberg 1997)
P. icmalea	Agyriaceae	Pertusariales	Lecanoromycetes	TLC	(Upreti et al. 2008)
Polyozosia hagenii	Ramalinaceae	Lecanorales	Lecanoromycetes	TLC	(Printzen and TØnsberg 1999)
Polysporinopsis rugulosa	Acarosporaceae	Acarosporales	Lecanoromycetes	TLC	(Huneck and Schreiber 1974)
Protoparmeliopsis gyrophorica	Lecanoraceae	Lecanorales	Lecanoromycetes	TLC	(Knudsen and Lendemer 2009)
Pseudocyphellaria argyracea	Lobariaceae	Peltigerales	Lecanoromycetes	TLC	(Pandit and Sharma 2012)
P. aurata	Lobariaceae	Peltigerales	Lecanoromycetes	TLC	(Kusmoro et al. 2018)
P. carpoloma	Lobariaceae	Peltigerales	Lecanoromycetes	TLC	(Wilkins and James 1979)
P. cinnamomea	Lobariaceae	Peltigerales	Lecanoromycetes	TLC	(Bryan and Elix 1976)
P. crocata	Lobariaceae	Peltigerales	Lecanoromycetes	HPLC-DAD-MS	(Gadea et al. 2017)
P. homalosticta	Lobariaceae	Peltigerales	Lecanoromycetes	TLC	(Din et al. 2008)
P. multifida	Lobariaceae	Peltigerales	Lecanoromycetes	TLC	(Kusmoro et al. 2018)
P. pilosella	Lobariaceae	Peltigerales	Lecanoromycetes	HPTLC	(Messuti et al. 2016)
P. quercifolia	Lobariaceae	Peltigerales	Lecanoromycetes	TLC	(Maass 1975)
P. sulphurea	Lobariaceae	Peltigerales	Lecanoromycetes	TLC	(Samsudin et al. 1998)
Pseudohepatica duidensis	Ramalinaceae	Lecanorales	Lecanoromycetes	TLC → HPTLC	(Marcano et al. 2000)
P. fernandezii	Ramalinaceae	Lecanorales	Lecanoromycetes	TLC	(Jørgensen 1993)
Psilolechia leprosa	Psilolechiaceae	Lecanorales	Lecanoromycetes	TLC	(Fryday and Coppins 1997)
Psora altotibetica	Psoraceae	Lecanorales	Lecanoromycetes	TLC	(Timdal et al. 2016)
P. californica	Psoraceae	Lecanorales	Lecanoromycetes	TLC	(Timdal et al. 2017)
P. hyporubescens	Psoraceae	Lecanorales	Lecanoromycetes	TLC	(Timdal et al. 2016)
P. indigirkae	Psoraceae	Lecanorales	Lecanoromycetes	TLC	(Timdal et al. 2016)
P. nipponica	Psoraceae	Lecanorales	Lecanoromycetes	Spot test	(Anderson 1962)
P. nitida	Psoraceae	Lecanorales	Lecanoromycetes	TLC	(Timdal et al. 2016)
P. pacifica	Psoraceae	Lecanorales	Lecanoromycetes	TLC	(Timdal et al. 2016)
Punctelia bolliana	Parmeliaceae	Lecanorales	Lecanoromycetes	TLC	(Spielmann and Marcelli 2008)
P. borreri	Parmeliaceae	Lecanorales	Lecanoromycetes	TLC	(Truong and Clerc 2003)
P. colombiana	Parmeliaceae	Lecanorales	Lecanoromycetes	TLC	(Spielmann and Marcelli 2008)
P. constantimontium	Parmeliaceae	Lecanorales	Lecanoromycetes	TLC	(Spielmann and Marcelli 2008)
P. microsticta	Parmeliaceae	Lecanorales	Lecanoromycetes	MS → NMR	(Maier et al. 1999)
P. stictica	Parmeliaceae	Lecanorales	Lecanoromycetes	TLC	(Truong and Clerc 2003)
P. subpraesignis	Parmeliaceae	Lecanorales	Lecanoromycetes	TLC	(Spielmann and Marcelli 2008)
Relicina luteoviridis	Parmeliaceae	Lecanorales	Lecanoromycetes	TLC → HPLC	(Din et al. 1999)
R. xanthoparmeliformis	Parmeliaceae	Lecanorales	Lecanoromycetes	TLC	(Elix and Nash 1995)
Remototrachyna sipmaniana	Parmeliaceae	Lecanorales	Lecanoromycetes	TLC	(Flakus et al. 2012)

Rhizocarpon alpicola	Rhizocarpaceae	Rhizocarpales	Lecanoromycetes	TLC	(Joshi et al. 2010)
R. austroamphibium	Rhizocarpaceae	Rhizocarpales	Lecanoromycetes	TLC	(Elix 2012)
R. distinctum	Rhizocarpaceae	Rhizocarpales	Lecanoromycetes	TLC	(Matwiejuk 2010)
R. effiguratum	Rhizocarpaceae	Rhizocarpales	Lecanoromycetes	TLC	(Poelt et al. 1988)
R. eupetraeum	Rhizocarpaceae	Rhizocarpales	Lecanoromycetes	TLC → HPLC	(Hamada et al. 1997)
R. geographicum	Rhizocarpaceae	Rhizocarpales	Lecanoromycetes	TLC	(Matwiejuk 2010)
R. grande	Rhizocarpaceae	Rhizocarpales	Lecanoromycetes	TLC	(Matwiejuk 2010)
R. inarense	Rhizocarpaceae	Rhizocarpales	Lecanoromycetes	TLC	(Poelt et al. 1988)
R. lecanorinum	Rhizocarpaceae	Rhizocarpales	Lecanoromycetes	TLC	(Matwiejuk 2010)
R. pusillum	Rhizocarpaceae	Rhizocarpales	Lecanoromycetes	HPLC	(Geyer et al. 1984)
R. viridiatrum	Rhizocarpaceae	Rhizocarpales	Lecanoromycetes	TLC	(Matwiejuk 2010)
Rhizoplaca melanophthalma	Lecanoraceae	Lecanorales	Lecanoromycetes	HPLC	(Leavitt et al. 2011)
Ricasolia quercizans	Lobariaceae	Peltigerales	Lecanoromycetes	TLC	(Ren et al. 2012)
R. tenuis	Lobariaceae	Peltigerales	Lecanoromycetes	TLC	(Moore 1969)
Rimularia cerebriformis	Trapeliaceae	Baeomycetales	Lecanoromycetes	TLC	(Kantvilas et al. 2008)
R. geumodoensis	Trapeliaceae	Baeomycetales	Lecanoromycetes	TLC → HPTLC	(Kondratyuk et al. 2016)
R. limborina	Trapeliaceae	Baeomycetales	Lecanoromycetes	TLC	(Yakovchenko et al. 2021)
R. Paradoxa	Trapeliaceae	Baeomycetales	Lecanoromycetes	TLC	(Timdal 2002)
R. ramboldiana	Trapeliaceae	Baeomycetales	Lecanoromycetes	TLC → HPLC	(Kantvilas and Elix 2007)
R. Subconcava	Trapeliaceae	Baeomycetales	Lecanoromycetes	TLC	(Timdal 2002)
Rinodina alba	Physciaceae	Caliciales	Lecanoromycetes	TLC → HPLC → MS → NMR	(Elix et al. 1995)
R. aspersa	Physciaceae	Caliciales	Lecanoromycetes	TLC	(Giralt et al. 1996)
R. canariensis	Physciaceae	Caliciales	Lecanoromycetes	TLC	(Matzer et al. 1994)
R. sicula	Physciaceae	Caliciales	Lecanoromycetes	TLC	(Sipman and Raus 2015)
Ropalospora hibernica	Ropalosporaceae	Umbilicariales	Lecanoromycetes	HPTLC	(Ekman 1993)
Schaereria cinereorufa	Schaereriaceae	Schaereriales	Lecanoromycetes	TLC	(Kantvilas 1999)
S. corticola	Schaereriaceae	Schaereriales	Lecanoromycetes	TLC	(Urbanavichus et al. 2020)
S. fuscocinerea	Schaereriaceae	Schaereriales	Lecanoromycetes	TLC	(Lumbsch et al. 2001)
Scoliciosporum gallurae	Scoliciosporaceae	Lecanorales	Lecanoromycetes	TLC	(Dymytrova 2011)
S. sarothamni	Scoliciosporaceae	Lecanorales	Lecanoromycetes	TLC	(Dymytrova 2011)
Solorina crocea	Peltigeraceae	Peltigerales	Lecanoromycetes	MS → NMR	(Okuyama et al. 1991)
Stereocaulon exutum	Stereocaulaceae	Lecanorales	Lecanoromycetes	TLC	(Park 1990)
Sticta lingulata	Lobariaceae	Peltigerales	Lecanoromycetes	TLC	(Kusmoro et al. 2018)
Stirtonia neotropica	Arthoniaceae	Arthoniales	Arthoniomycetes	TLC	(Seavey and Seavey 2015)
Syncesia graphica	Roccellaceae	Arthoniales	Arthoniomycetes	TLC	(Aptroot and Sparrius 2008)
Tephromela atra	Mycoblastaceae	Lecanorales	Lecanoromycetes	TLC	(Sipman and Raus 2002)

Trapelia calyciformis	Trapeliaceae	Baeomycetales	Lecanoromycetes	TLC	(Dou et al. 2021)
T. coarctata	Trapeliaceae	Baeomycetales	Lecanoromycetes	TLC	(Orange 2018)
T. collaris	Trapeliaceae	Baeomycetales	Lecanoromycetes	TLC	(Orange 2018)
T. corticola	Trapeliaceae	Baeomycetales	Lecanoromycetes	TLC	(Urbanavichene and Urbanavichus 2019)
T. crystallifera	Trapeliaceae	Baeomycetales	Lecanoromycetes	TLC → HPLC	(Kantvilas and Elix 2007)
T. elacista	Trapeliaceae	Baeomycetales	Lecanoromycetes	TLC	(Orange 2018)
T. glebulosa	Trapeliaceae	Baeomycetales	Lecanoromycetes	TLC	(Orange 2018)
T. involuta	Trapeliaceae	Baeomycetales	Lecanoromycetes	TLC	(Orange 2018)
T. lilacea	Trapeliaceae	Baeomycetales	Lecanoromycetes	TLC → HPLC	(Kantvilas and Elix 2007)
T. macrospora	Trapeliaceae	Baeomycetales	Lecanoromycetes	TLC → HPLC	(Kantvilas and Elix 2007)
T. obtegens	Trapeliaceae	Baeomycetales	Lecanoromycetes	TLC	(Orange 2018)
T. placodioides	Trapeliaceae	Baeomycetales	Lecanoromycetes	TLC	(Orange 2018)
T. sitiens	Trapeliaceae	Baeomycetales	Lecanoromycetes	TLC	(Orange 2018)
T. thieleana	Trapeliaceae	Baeomycetales	Lecanoromycetes	TLC	(Kantvilas et al. 2014)
T. tristis	Trapeliaceae	Baeomycetales	Lecanoromycetes	TLC	(Orange 2018)
Trapeliopsis bisorediata	Trapeliaceae	Baeomycetales	Lecanoromycetes	TLC	(McCune et al. 2002)
T. flexuosa	Trapeliaceae	Baeomycetales	Lecanoromycetes	TLC	(Lumbsch et al. 2001)
T. granulosa	Trapeliaceae	Baeomycetales	Lecanoromycetes	TLC	(Lumbsch et al. 2001)
T. pseudogranulosa	Trapeliaceae	Baeomycetales	Lecanoromycetes	TLC	(Lumbsch et al. 2001)
T. studerae	Trapeliaceae	Baeomycetales	Lecanoromycetes	TLC	(Aptroot et al. 2018)
T. wallrothii	Trapeliaceae	Baeomycetales	Lecanoromycetes	Spot test	(Fryday and Van Den Boom 2019)
Umbilicaria antarctica	Umbilicariaceae	Umbilicariales	Lecanoromycetes	Raman spectroscopy	(Miralles et al. 2015)
U. cinereorufescens	Umbilicariaceae	Umbilicariales	Lecanoromycetes	NMR	(Narui et al. 1996b)
U. hyperborea	Umbilicariaceae	Umbilicariales	Lecanoromycetes	HPLC	(Fahselt and Alstrup 1997)
U. indica	Umbilicariaceae	Umbilicariales	Lecanoromycetes	TLC	(Rao and Shripathy 1976)
U. kisovana	Umbilicariaceae	Umbilicariales	Lecanoromycetes	TLC	(Park 1990)
U. krascheninnikovii	Umbilicariaceae	Umbilicariales	Lecanoromycetes	HPLC	(Fahselt and Alstrup 1997)
U. mammulata	Umbilicariaceae	Umbilicariales	Lecanoromycetes	TLC	(BeGora and Fahselt 2000)
U. muhlenbergii	Umbilicariaceae	Umbilicariales	Lecanoromycetes	TLC	(Mirando and Fahselt 1978)
U. pulvinaria	Umbilicariaceae	Umbilicariales	Lecanoromycetes	TLC → HPLC	(Davydov et al. 2011)
U. spodochroa	Umbilicariaceae	Umbilicariales	Lecanoromycetes	TLC → IR → MS → NMR	(Solberg 1975)
U. torrefacta	Umbilicariaceae	Umbilicariales	Lecanoromycetes	TLC → MS/MS → negative fast atom bombardment (NI-FAB)	(Holzmann and Leuckert 1990)
Upretia squamulosa	Umbilicariaceae	Umbilicariales	Lecanoromycetes	HPLC	(Zhang et al. 2019)
Usnea ceratina	Parmeliaceae	Lecanorales	Lecanoromycetes	TLC	(Kusmoro et al. 2018)
U. subfloridana	Parmeliaceae	Lecanorales	Lecanoromycetes	TLC	(Kusmoro et al. 2018)

U. trachycarpa	Parmeliaceae	Lecanorales	Lecanoromycetes	TLC → HPLC	(Elix et al. 2007)
Varicellaria hemisphaerica	Ochrolechiaceae	Pertusariales	Lecanoromycetes	TLC	(Urbanavichus et al. 2020)
Vigneronia spieri	Roccellaceae	Arthoniales	Arthoniomycetes	TLC	(Aptroot and Sparrius 2008)
Xanthoparmelia aff. keralensis	Parmeliaceae	Lecanorales	Lecanoromycetes	TLC	(Alstrup et al. 2010)
X. attica	Parmeliaceae	Lecanorales	Lecanoromycetes	TLC	(Sipman and Raus 2015)
X. delisei	Parmeliaceae	Lecanorales	Lecanoromycetes	TLC	(Tsurykau et al. 2018)
X. fangii	Parmeliaceae	Lecanorales	Lecanoromycetes	TLC	(Elix 2006)
X. glabrans	Parmeliaceae	Lecanorales	Lecanoromycetes	TLC	(Giordani et al. 2003)
X. halei	Parmeliaceae	Lecanorales	Lecanoromycetes	TLC → HPLC	(Esslinger et al. 1993)
X. loxodes	Parmeliaceae	Lecanorales	Lecanoromycetes	TLC	(Tsurykau et al. 2018)
X. luteonotata	Parmeliaceae	Lecanorales	Lecanoromycetes	TLC	(Giordani et al. 2003)
X. peltata	Parmeliaceae	Lecanorales	Lecanoromycetes	TLC	(Henssen 1991)
X. perrugata	Parmeliaceae	Lecanorales	Lecanoromycetes	TLC	(Giordani et al. 2003)
X. pulla	Parmeliaceae	Lecanorales	Lecanoromycetes	TLC	(Tsurykau et al. 2018)
X. sleei	Parmeliaceae	Lecanorales	Lecanoromycetes	TLC → HPLC	(Elix 2004)
X. teydea	Parmeliaceae	Lecanorales	Lecanoromycetes	TLC → HPLC	(Perez-Vargas et al. 2010)
X. verruculifera	Parmeliaceae	Lecanorales	Lecanoromycetes	TLC	(Sipman and Raus 2015)
Yoshimuriella fendleri	Lobariaceae	Peltigerales	Lecanoromycetes	HPLC	(Stenroos et al. 2003)
**Hiascic acid**					
Aspiciliopsis macrophthalma	Trapeliaceae	Baeomycetales	Lecanoromycetes	TLC → HPLC	(Galloway 2010)
Bulbothrix goebelii	Parmeliaceae	Lecanorales	Lecanoromycetes	TLC	(Bungartz et al. 2013)
Cetrariella delisei	Parmeliaceae	Lecanorales	Lecanoromycetes	TLC → HPLC → MS → NMR	(Narui et al. 1998)
Hypotrachyna revoluta	Parmeliaceae	Lecanorales	Lecanoromycetes	TLC	(Dey 1978)
Lasallia caroliniana	Umbilicariaceae	Umbilicariales	Lecanoromycetes	TLC → HPLC	(Narui et al. 1996a)
Ochrolechia splendens	Ochrolechiaceae	Pertusariales	Lecanoromycetes	TLC → HPLC	(Lumbsch et al. 2003)
Parmelinopsis minarum	Parmeliaceae	Lecanorales	Lecanoromycetes	TLC	(Eliasaro and Adler 2000)
P. Spumosa	Parmeliaceae	Lecanorales	Lecanoromycetes	TLC	(Benatti 2012a)
Peltigeropsis vainioi	Peltigeraceae	Peltigerales	Lecanoromycetes	TLC	(Marcano et al. 1997)
Pertusaria corallina	Pertusariaceae	Pertusariales	Lecanoromycetes	Spot test → Microcrystal	(Kantvilas 2016)
Placopsis auriculata	Trapeliaceae	Baeomycetales	Lecanoromycetes	TLC → HPLC	(Lumbsch et al. 1993)
P. fusciduloides	Trapeliaceae	Baeomycetales	Lecanoromycetes	TLC → HPLC	(Galloway 2010)
Placopsis gelidioides	Trapeliaceae	Baeomycetales	Lecanoromycetes	TLC → HPLC	(Galloway 2010)
Schaereria fuscocinerea	Schaereriaceae	Schaereriales	Lecanoromycetes	TLC	(Lunke et al. 1996)
Trapelia lilacea	Trapeliaceae	Baeomycetales	Lecanoromycetes	TLC → HPLC	(Kantvilas and Elix 2007)
Umbilicaria africana	Umbilicariaceae	Umbilicariales	Lecanoromycetes	TLC → HPLC	(Narui et al. 1996a)
U. angulata	Umbilicariaceae	Umbilicariales	Lecanoromycetes	TLC → HPLC	(Narui et al. 1996a)

U. arctica	Umbilicariaceae	Umbilicariales	Lecanoromycetes	TLC → HPLC	(Narui et al. 1996a)
U. deusta	Umbilicariaceae	Umbilicariales	Lecanoromycetes	TLC → HPLC	(Narui et al. 1996a)
U. esculenta	Umbilicariaceae	Umbilicariales	Lecanoromycetes	TLC → HPLC	(Narui et al. 1996a)
U. kisovana	Umbilicariaceae	Umbilicariales	Lecanoromycetes	TLC → HPLC	(Narui et al. 1996a)
U. mammulata	Umbilicariaceae	Umbilicariales	Lecanoromycetes	TLC → HPLC	(Narui et al. 1996a)
U. muhlenbergii	Umbilicariaceae	Umbilicariales	Lecanoromycetes	TLC → HPLC	(Narui et al. 1996a)
U. nylanderiana	Umbilicariaceae	Umbilicariales	Lecanoromycetes	TLC → HPLC	(Narui et al. 1996a)
U. proboscidea	Umbilicariaceae	Umbilicariales	Lecanoromycetes	TLC → HPLC	(Narui et al. 1996a)
U. spodochroa	Umbilicariaceae	Umbilicariales	Lecanoromycetes	TLC → HPLC	(Narui et al. 1996a)
U. yunnana	Umbilicariaceae	Umbilicariales	Lecanoromycetes	TLC → HPLC	(Narui et al. 1996a)
Usnea pectinata	Parmeliaceae	Lecanorales	Lecanoromycetes	Spot test	(Jannah et al. 2020)
**Lasallic acid**					
Lasallia asiae-orientalis	Umbilicariaceae	Umbilicariales	Lecanoromycetes	TLC → HPLC → MS → NMR	(Narui et al. 1998)
L. mayebarae	Umbilicariaceae	Umbilicariales	Lecanoromycetes	TLC → HPLC → MS → NMR	(Narui et al. 1998)
L. papulosa	Umbilicariaceae	Umbilicariales	Lecanoromycetes	TLC → HPLC → MS → NMR	(Narui et al. 1998)
Umbilicaria cinereorufescens	Umbilicariaceae	Umbilicariales	Lecanoromycetes	TLC → HPLC	(Narui et al. 1996a)
U. sinorientalis	Umbilicariaceae	Umbilicariales	Lecanoromycetes	TLC → HPLC	(Narui et al. 1996a)
**Methylgyrophoric acid**					
Angiactis spinicola	Roccellaceae	Arthoniales	Arthoniomycetes	TLC	(Aptroot et al. 2008)
Baeomyces placophyllus	Baeomycetaceae	Baeomycetales	Lecanoromycetes	HPLC-DAD	(Yoshimura et al. 1994)
Enterographa zephyri	Roccellaceae	Arthoniales	Arthoniomycetes	TLC	(Cáceres et al. 2014)
Lobaria linita	Lobariaceae	Peltigerales	Lecanoromycetes	TLC → NMR	(Maass 1975b)
L. ravenelii	Lobariaceae	Peltigerales	Lecanoromycetes	TLC	(Jordan 1972)
Lobariella crenulata	Lobariaceae	Peltigerales	Lecanoromycetes	TLC	(Moncada et al. 2013)
L. ecoticata	Lobariaceae	Peltigerales	Lecanoromycetes	TLC	(Moncada et al. 2013)
L. pallida	Lobariaceae	Peltigerales	Lecanoromycetes	TLC	(Moncada et al. 2013)
L. peltata	Lobariaceae	Peltigerales	Lecanoromycetes	TLC	(Moncada et al. 2013)
L. soredians	Lobariaceae	Peltigerales	Lecanoromycetes	TLC	(Moncada et al. 2013)
Nephroma gyelnikii	Nephromataceae	Peltigerales	Lecanoromycetes	TLC → MS	(Renner et al. 1982)
N. pseudoparile	Nephromataceae	Peltigerales	Lecanoromycetes	TLC → MS	(Renner et al. 1982)
Peltigera aphthosa	Peltigeraceae	Peltigerales	Lecanoromycetes	TLC-→ IR → MS → NMR	(Maass 1975c)
P. britannica	Peltigeraceae	Peltigerales	Lecanoromycetes	TLC	(Goward et al. 1995)
P. chionophila	Peltigeraceae	Peltigerales	Lecanoromycetes	TLC	(Goward and Goffinet 2000)
P. cichoracea	Peltigeraceae	Peltigerales	Lecanoromycetes	TLC	(Sérusiaux and Goffinet 2009)
P. collina	Peltigeraceae	Peltigerales	Lecanoromycetes	TLC	(Wei et al. 2009)
P. didactyla	Peltigeraceae	Peltigerales	Lecanoromycetes	TLC	(Goffinet and Hastings 1995)

P. dissecta	Peltigeraceae	Peltigerales	Lecanoromycetes	TLC	(Purvis and James 1993)
P. dolichorhiza	Peltigeraceae	Peltigerales	Lecanoromycetes	TLC	(Sérusiaux and Goffinet 2009)
P. elisabethae	Peltigeraceae	Peltigerales	Lecanoromycetes	TLC	(Goffinet and Miadlikowska 1999)
P. extenuata	Peltigeraceae	Peltigerales	Lecanoromycetes	TLC	(Sérusiaux and Goffinet 2009)
P. frippii	Peltigeraceae	Peltigerales	Lecanoromycetes	TLC	(Holtan-Hartwig 1988)
P. hymenina	Peltigeraceae	Peltigerales	Lecanoromycetes	TLC	(Goward et al. 1995)
P. lambinonii	Peltigeraceae	Peltigerales	Lecanoromycetes	TLC	(Goffinet and Hastings 1995)
P. leucophlebia	Peltigeraceae	Peltigerales	Lecanoromycetes	TLC	(Goward et al. 1995)
P. malacea	Peltigeraceae	Peltigerales	Lecanoromycetes	TLC	(Goward et al. 1995)
P. melanorrhiza	Peltigeraceae	Peltigerales	Lecanoromycetes	TLC	(Purvis and James 1993)
P. nana	Peltigeraceae	Peltigerales	Lecanoromycetes	TLC	(Sérusiaux and Goffinet 2009)
P. neckeri	Peltigeraceae	Peltigerales	Lecanoromycetes	TLC	(Goward et al. 1995)
P. neopolydactyla	Peltigeraceae	Peltigerales	Lecanoromycetes	TLC	(Goward et al. 1995)
P. occidentalis	Peltigeraceae	Peltigerales	Lecanoromycetes	TLC	(Goward et al. 1995)
P. pacijca	Peltigeraceae	Peltigerales	Lecanoromycetes	TLC	(Goward et al. 1995)
P. phyllidiosa	Peltigeraceae	Peltigerales	Lecanoromycetes	TLC	(Goffinet and Miadlikowska 1999)
P. polydacrylon	Peltigeraceae	Peltigerales	Lecanoromycetes	TLC	(Goward et al. 1995)
P. retifoveata	Peltigeraceae	Peltigerales	Lecanoromycetes	TLC	(Goward et al. 1995)
P. scabrosa	Peltigeraceae	Peltigerales	Lecanoromycetes	TLC	(Goward et al. 1995)
P. scabrosella	Peltigeraceae	Peltigerales	Lecanoromycetes	TLC	(Holtan-Hartwig 1988)
P. seneca	Peltigeraceae	Peltigerales	Lecanoromycetes	TLC	(Magain et al. 2016)
P. serusiauxii	Peltigeraceae	Peltigerales	Lecanoromycetes	TLC	(Magain et al. 2020)
P. sumatrana	Peltigeraceae	Peltigerales	Lecanoromycetes	TLC	(Sérusiaux and Goffinet 2009)
P. tartarea	Peltigeraceae	Peltigerales	Lecanoromycetes	TLC	(Vitikainen 2006)
P. ulcerata	Peltigeraceae	Peltigerales	Lecanoromycetes	TLC	(Sérusiaux and Goffinet 2009)
P. venosa	Peltigeraceae	Peltigerales	Lecanoromycetes	TLC	(Goward et al. 1995)
P. weberi	Peltigeraceae	Peltigerales	Lecanoromycetes	TLC	(Sérusiaux and Goffinet 2009)
Pseudocyphellaria carpoloma	Lobariaceae	Peltigerales	Lecanoromycetes	TLC	(Wilkins and James 1979)
P. crocata	Lobariaceae	Peltigerales	Lecanoromycetes	TLC → MS → NMR	(Maass 1975a)
P. homalosticta	Lobariaceae	Peltigerales	Lecanoromycetes	TLC	(Din et al. 2008)
P. quercifolia	Lobariaceae	Peltigerales	Lecanoromycetes	TLC	(Maass 1975)
P. sulphurea	Lobariaceae	Peltigerales	Lecanoromycetes	TLC	(Samsudin et al. 1998)
Solorina crocea	Peltigeraceae	Peltigerales	Lecanoromycetes	MS → NMR	(Okuyama et al. 1991)
**Methylhiascic acid**					
Micarea borbonica	Pilocarpaceae	Lecanorales	Lecanoromycetes	Spot test	(Brand et al. 2014)
M. peliocarpa	Pilocarpaceae	Lecanorales	Lecanoromycetes	Spot test	(Brand et al. 2014)

Parmelinopsis afrorevoluta	Parmeliaceae	Lecanorales	Lecanoromycetes	TLC	(Divakar and Upreti 2005)
**Ovoic acid**					
Buellia imshaugii	Caliciaceae	Caliciales	Lecanoromycetes	HPLC	(Giralt and Elix 2010)
Cryptothecia scripta	Arthoniaceae	Arthoniales	Arthoniomycetes	TLC	(Bajpai et al. 2017)
Dimelaena oreina	Caliciaceae	Caliciales	Lecanoromycetes	TLC	(Obermayer et al. 2004)
Herpothallon japonicum	Arthoniaceae	Arthoniales	Arthoniomycetes	TLC	(Frisch et al. 2014)
H. rubrocinctoides	Arthoniaceae	Arthoniales	Arthoniomycetes	TLC	(Frisch et al. 2014)
Lasallia caroliniana	Umbilicariaceae	Umbilicariales	Lecanoromycetes	TLC → HPLC	(Narui et al. 1996a)
Montanelia tominii	Parmeliaceae	Lecanorales	Lecanoromycetes	TLC → HPLC → MS → NMR	(Narui et al. 1998)
Rinodina aspersa	Physciaceae	Caliciales	Lecanoromycetes	TLC	(Giralt et al. 1996)
Umbilicaria angulata	Umbilicariaceae	Umbilicariales	Lecanoromycetes	TLC → HPLC	(Narui et al. 1996a)
U. cylindrica	Umbilicariaceae	Umbilicariales	Lecanoromycetes	TLC → HPLC	(Narui et al. 1996a)
U. decussata	Umbilicariaceae	Umbilicariales	Lecanoromycetes	TLC → HPLC	(Narui et al. 1996a)
U. havaasii	Umbilicariaceae	Umbilicariales	Lecanoromycetes	TLC → HPLC	(Narui et al. 1996a)
U. krascheninnikovii	Umbilicariaceae	Umbilicariales	Lecanoromycetes	HPLC	(Fahselt and Alstrup 1997)
U. proboscidea	Umbilicariaceae	Umbilicariales	Lecanoromycetes	TLC → HPLC	(Narui et al. 1996a)
U. torrefacta	Umbilicariaceae	Umbilicariales	Lecanoromycetes	TLC → MS/MS → negative fast atom bombardment (NI-FAB)	(Holzmann and Leuckert 1990)
**Tenuiorin**					
Leioderma pycnophorum	Pannariaceae	Peltigerales	Lecanoromycetes	LPCC → TLC → NMR	(Piovano et al. 1995)
Lobaria linita	Lobariaceae	Peltigerales	Lecanoromycetes	TLC → NMR	(Maass 1975b)
Nephroma gyelnikii	Nephromataceae	Peltigerales	Lecanoromycetes	TLC → MS	(Renner et al. 1982)
N. pseudoparile	Nephromataceae	Peltigerales	Lecanoromycetes	TLC → MS	(Renner et al. 1982)
Peltigera aphthosa	Peltigeraceae	Peltigerales	Lecanoromycetes	TLC-→ IR → MS → NMR	(Maass 1975c)
P. britannica	Peltigeraceae	Peltigerales	Lecanoromycetes	TLC	(Goward et al. 1995)
P. canina	Peltigeraceae	Peltigerales	Lecanoromycetes	TLC → IR → MS → NMR	(Solberg 1975)
P. cf. neglecta	Peltigeraceae	Peltigerales	Lecanoromycetes	TLC	(Samsudin et al. 1998)
P. chionophila	Peltigeraceae	Peltigerales	Lecanoromycetes	TLC	(Goward and Goffinet 2000)
P. cichoracea	Peltigeraceae	Peltigerales	Lecanoromycetes	TLC	(Sérusiaux and Goffinet 2009)
P. collina	Peltigeraceae	Peltigerales	Lecanoromycetes	TLC	(Wei et al. 2009)
P. dissecta	Peltigeraceae	Peltigerales	Lecanoromycetes	TLC	(Purvis and James 1993)
P. dolichorhiza	Peltigeraceae	Peltigerales	Lecanoromycetes	TLC	(Sérusiaux and Goffinet 2009)
P. elisabethae	Peltigeraceae	Peltigerales	Lecanoromycetes	TLC	(Goward et al. 1995)
P. frippii	Peltigeraceae	Peltigerales	Lecanoromycetes	TLC	(Holtan-Hartwig 1988)
P. hymenina	Peltigeraceae	Peltigerales	Lecanoromycetes	TLC	(Goward et al. 1995)
P. leucophlebia	Peltigeraceae	Peltigerales	Lecanoromycetes	TLC	(Goward et al. 1995)

P. malacea	Peltigeraceae	Peltigerales	Lecanoromycetes	TLC	(Goward et al. 1995)
P. melanorrhiza	Peltigeraceae	Peltigerales	Lecanoromycetes	TLC	(Purvis and James 1993)
P. nana	Peltigeraceae	Peltigerales	Lecanoromycetes	TLC	(Sérusiaux and Goffinet 2009)
P. neckeri	Peltigeraceae	Peltigerales	Lecanoromycetes	TLC	(Goward et al. 1995)
P. neopolydactyla	Peltigeraceae	Peltigerales	Lecanoromycetes	TLC	(Goward et al. 1995)
P. occidentalis	Peltigeraceae	Peltigerales	Lecanoromycetes	TLC	(Goward et al. 1995)
P. pacijca	Peltigeraceae	Peltigerales	Lecanoromycetes	TLC	(Goward et al. 1995)
P. phyllidiosa	Peltigeraceae	Peltigerales	Lecanoromycetes	TLC	(Goffinet and Miadlikowska 1999)
P. polydacrylon	Peltigeraceae	Peltigerales	Lecanoromycetes	TLC	(Goward et al. 1995)
P. polydactyla	Peltigeraceae	Peltigerales	Lecanoromycetes	TLC	(Goffinet and Miadlikowska 1999)
P. praetextata	Peltigeraceae	Peltigerales	Lecanoromycetes	TLC	(Wei et al. 2009)
P. retifoveata	Peltigeraceae	Peltigerales	Lecanoromycetes	TLC	(Goward et al. 1995)
P. scabrosa	Peltigeraceae	Peltigerales	Lecanoromycetes	TLC	(Goward et al. 1995)
P. scabrosella	Peltigeraceae	Peltigerales	Lecanoromycetes	TLC	(Holtan-Hartwig 1988)
P. seneca	Peltigeraceae	Peltigerales	Lecanoromycetes	TLC	(Magain et al. 2016)
P. serusiauxii	Peltigeraceae	Peltigerales	Lecanoromycetes	TLC	(Magain et al. 2020)
P. sumatrana	Peltigeraceae	Peltigerales	Lecanoromycetes	TLC	(Sérusiaux and Goffinet 2009)
P. venosa	Peltigeraceae	Peltigerales	Lecanoromycetes	TLC	(Goward et al. 1995)
P. weberi	Peltigeraceae	Peltigerales	Lecanoromycetes	TLC	(Sérusiaux and Goffinet 2009)
Pseudocyphellaria carpoloma	Lobariaceae	Peltigerales	Lecanoromycetes	TLC	(Wilkins and James 1979)
P. coriifolia	Lobariaceae	Peltigerales	Lecanoromycetes	NA	(Fritis et al. 2013)
P. crocata	Lobariaceae	Peltigerales	Lecanoromycetes	TLC → MS → NMR	(Bryan and Elix 1976)
P. faveolata	Lobariaceae	Peltigerales	Lecanoromycetes	TLC → MS → NMR	(Elix and Lajide 1981)
P. homalosticta	Lobariaceae	Peltigerales	Lecanoromycetes	TLC	(Din et al. 2008)
P. mougeotiana	Lobariaceae	Peltigerales	Lecanoromycetes	TLC	(Corbett and Cumming 1971)
P. neglecta	Lobariaceae	Peltigerales	Lecanoromycetes	TLC → MS → NMR	(Bryan and Elix 1976)
P. nudata	Lobariaceae	Peltigerales	Lecanoromycetes	TLC → NMR	(Cuellar et al. 2008)
P. quercifolia	Lobariaceae	Peltigerales	Lecanoromycetes	TLC	(Maass 1975)
P. sulphurea	Lobariaceae	Peltigerales	Lecanoromycetes	TLC	(Samsudin et al. 1998)
**Trivaric acid**					
Ramalina americana	Ramalinaceae	Lecanorales	Lecanoromycetes	TLC → HPLC	(Culberson et al. 1999)
**Umbilicaric acid**					
Bulbothricella amazonensis	Parmeliaceae	Lecanorales	Lecanoromycetes	TLC	(Marcano et al. 1996)
Rinodina alba	Physciaceae	Caliciales	Lecanoromycetes	TLC → HPLC → MS → NMR	(Elix et al. 1995)
R. aspersa	Physciaceae	Caliciales	Lecanoromycetes	TLC	(Giralt et al. 1996)
Umbilicaria arctica	Umbilicariaceae	Umbilicariales	Lecanoromycetes	TLC → HPLC	(Narui et al. 1996a)

U. cinerascens	Umbilicariaceae	Umbilicariales	Lecanoromycetes	TLC → HPLC	(Narui et al. 1996a)
U. freyi	Umbilicariaceae	Umbilicariales	Lecanoromycetes	TLC → HPLC	(Narui et al. 1996a)
U. grisea	Umbilicariaceae	Umbilicariales	Lecanoromycetes	TLC → HPLC	(Narui et al. 1996a)
U. hyperborea	Umbilicariaceae	Umbilicariales	Lecanoromycetes	HPLC	(Fahselt and Alstrup 1997)
U. hypococcinea	Umbilicariaceae	Umbilicariales	Lecanoromycetes	TLC → HPLC	(Narui et al. 1996a)
U. laevis	Umbilicariaceae	Umbilicariales	Lecanoromycetes	TLC → HPLC	(Narui et al. 1996a)
U. magnussonii	Umbilicariaceae	Umbilicariales	Lecanoromycetes	TLC → HPLC	(Narui et al. 1996a)
U. microphylla	Umbilicariaceae	Umbilicariales	Lecanoromycetes	TLC → HPLC	(Narui et al. 1996a)
U. muhlenbergii	Umbilicariaceae	Umbilicariales	Lecanoromycetes	TLC → HPLC	(Narui et al. 1996a)
U. nylanderiana	Umbilicariaceae	Umbilicariales	Lecanoromycetes	TLC → HPLC	(Narui et al. 1996a)
U. pallens	Umbilicariaceae	Umbilicariales	Lecanoromycetes	TLC → HPLC	(Narui et al. 1996a)
U. polyphylla	Umbilicariaceae	Umbilicariales	Lecanoromycetes	TLC → HPLC → MS → NMR	(Narui et al. 1998)
U. polyrrhiza	Umbilicariaceae	Umbilicariales	Lecanoromycetes	TLC → HPLC	(Narui et al. 1996a)
U. ruebeliana	Umbilicariaceae	Umbilicariales	Lecanoromycetes	TLC → HPLC	(Narui et al. 1996a)
U. spodochroa	Umbilicariaceae	Umbilicariales	Lecanoromycetes	TLC → HPLC	(Narui et al. 1996a)
U. subglabra	Umbilicariaceae	Umbilicariales	Lecanoromycetes	TLC → HPLC	(Narui et al. 1996a)
U. vellea	Umbilicariaceae	Umbilicariales	Lecanoromycetes	TLC → HPLC	(Narui et al. 1996a)
U. virginis	Umbilicariaceae	Umbilicariales	Lecanoromycetes	TLC → HPLC	(Narui et al. 1996a)

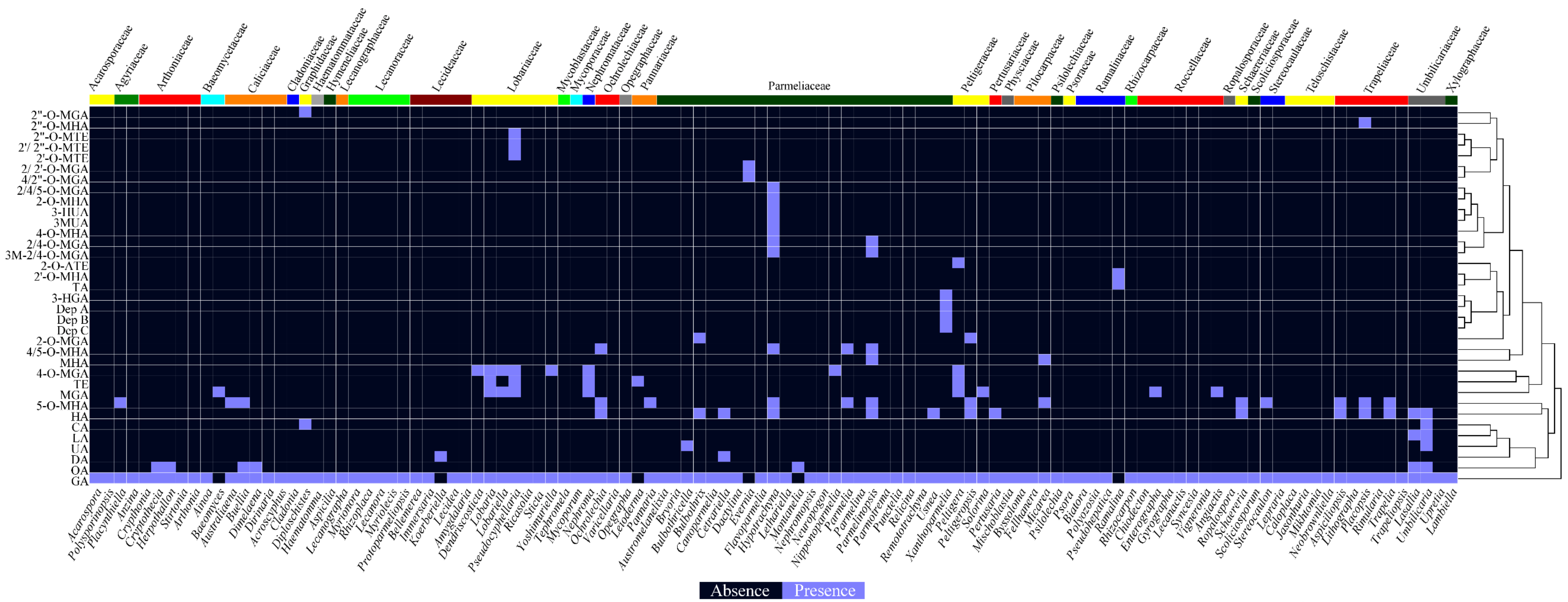

Figure S1 Genera-based distribution of different tridepsides (Tridepsides' abbreviations → **GA**: Gyrophoric acid/ **MGA**: Methylgyrophoric acid/ **TE**: Tenuiorin/ **5-O-MHA**: 5-*O*-methylhiascic acid/ **HA**: Hiascic acid**/ 4-O-MGA**: 4-*O*-methylgyrophoric acid/ **UA**: Umbilicaric acid/ **4,5-O-MHA**: 4,5-di-*O*-methylhiascic acid/ **OA**: Ovoic acid/ **3M-2,4-O-MGA**: 3-methoxy-2,4-di-*O*-methylgyrophoric acid/ **2,4-O-MGA**: 2,4-di-*O*-methylgyrophoric acid/ **2-O-ATE**: 2-*O*-acetyltenuiorin/ **LA**: Lasallic acid/ **CA**: Crustinic acid/ **MHA**: Methylhiascic acid/ **DA**: Deliseic acid/ **2,4,5-*O*-MGA**: 2,4,5-tri-*O*-methylhiascic acid/ **2-O-MGA**: 2-*O*-methylgyrophoric acid/ **2"-O-MGA**: 2"-*O*-methylgyrophoric acid/ **TA**: Trivaric acid/ **Dep C**: depsidellin C/ **Dep B**: depsidellin B/ **Dep A**: depsidellin A/ **2"-O-MTE**: 2"-*O*-methyltenuiorin/ **2'-O-MTE**: 2'-*O*-methyltenuiorin/ **2', 2"-O-MTE**: 2',2"-di-*O*-methyltenuiorin/ **3-HUA**: 3-hydroxyumbilicaric acid/ **3MUA**: 3-methoxyumbilicaric acid/ **2"-O-MHA**: 2"-*O*-methylhiascic acid**/ 4-O-MHA**: 4-*O*-methylhiascic acid/ **2'-O-MHA**: 2'-*O*-Methylhiascic acid/ **2-O-MHA**: 2-*O*-methylhiascic acid/ **3-HGA**: 3-hydroxygyrophoric acid/ **2, 2'-O-MGA**: 2,2'-di-*O*-methylgyrophoric acid/ **4,2"-O-MGA**: 4,2"-di-*O*-methylgyrophoric acid)

References

Ahn, C., Moon, K.H., 2016. *Parmotrema clavuliferum* and *P. reticulatum* are independent species. J. Species Res. 5, 254–260. https://doi.org/10.12651/jsr.2016.5.2.254

Almeda, F., Dey, J.P., 1973. Chemical and Nomenclatural Notes on the *Parmelia xanthina* Complex. Bryologist 76, 541. https://doi.org/10.2307/3241414

Alstrup, V., Aptroot, A., Divakar, P.K., LaGreca, S., Tibell, L., 2010. Lichens from Tanzania and Kenya III. Macrolichens and calicioid lichens. Cryptogam. Mycol. 31, 333–351.

Anderson, R.A., 1962. The Lichen Flora of the Dakota Sandstone in North-Central Colorado. Bryologist 65, 242. https://doi.org/10.2307/3241047

Aptroot, A., Barreto, F.M.O., Penã, D.A.R., Cáceres, M.E.D.S., Peña, D.A.R., Cáceres, M.E.D.S., 2018. A new lineage of fruticose lichens that belongs to the Trapeliaceae (Trapeliales, Ascomycota) from Alagoas, NE Brazil. Bryologist 121, 529–535. https://doi.org/10.1639/0007-2745-121.4.529

Aptroot, A., Cáceres, M.E.D.S., 2018. New Species and New Records of Lichens from Inselbergs and Surrounding Atlantic Rain Forest in the Chapada Diamantina (Bahia, Brazil). Herzogia 31, 359. https://doi.org/10.13158/heia.31.1.2018.359

Aptroot, A., Cáceres, M.E.D.S., 2017. New Arthoniales from Amapá (Amazonian North Brazil) show unexpected relationships. Lichenologist 49, 607–615. https://doi.org/10.1017/S0024282917000500

Aptroot, A., Sparrius, L.B., 2008. Crustose Roccellaceae in the Galapagos Islands, with the new species *Schismatomma spierii*. Bryologist 111, 659–666. https://doi.org/10.1639/0007-2745-111.4.659

Aptroot, A., Sparrius, L.B., LaGreca, S., Bungartz, F., 2008. *Angiactis*, a new crustose lichen genus in the Roccellaceae, with species from Bermuda, the Galápagos Islands and Australia. Bryologist 111, 510–516. https://doi.org/10.1639/0007-2745(2008)111[510:AANCLG]2.0.CO;2

Asahina, Y., Kusaka, K., 1942. Lichen substances. XCVII. Hiascinic acid, a new tridepside. Yakugaku Zasshi 62, 339–343.

Bajpai, R., Joseph, S., Upreti, D.K., 2017. Additional distributional records of the lichen genus *Cryptothecia* in India. Cryptogam Biodivers. Assess. 2. https://doi.org/10.21756/cab.v2i02.11118

Bawingan, P.A., Lardizaval, M.P., Rosuman, P.F., Fajardo, W.T., Azuelo, A., Elix, J.A., Hur, J.-S., 2017. Philippine species of *Parmotrema* (Ascomycota, Parmeliaceae). Philipp. J. Sci. 146, 145–158.

BeGora, M.D., Fahselt, D., 2000. An alternative method for the quantification of lichen secondary products. Bryologist 103, 563–567. https://doi.org/10.1639/0007-2745(2000)103[0563:AAMFTQ]2.0.CO;2

Benatti, M.N., 2012a. New species of *Bulbothrix* Hale containing gyrophoric acid from Brazil. Mycology 3, 127–131. https://doi.org/10.1080/21501203.2011.637089

Benatti, M.N., 2012b. A worldwide key for the genus *Parmelinopsis* Elix & Hale (Parmeliaceae; Lichenized Ascomycetes). Opusc. Philolichenum 11, 304–312.

Benatti, M.N., Elix, J.A., 2012. The true identity of *Bulbothrix goebelii* (Zenker) Hale and the re-establishment of some of its synonyms as accepted species. Lichenologist 44, 813–826. https://doi.org/10.1017/S0024282912000436

Benatti, M.N., Käffer, M.I., De Azevedo Martins, S.M., De Lemos, A.B., 2015. *Bulbothrix bulbillosa*, a Presumed Galapagos Endemic, is common in Rio Grande do sul State, Brazil (Parmeliaceae, Lichenized Ascomycota). Cryptogam. Mycol. 36, 109–114. https://doi.org/10.7872/crym.v36.iss1.2015.109

Biju, H., Bagool, R.G., Nayaka, S., 2010. Additions to the lichen flora of Kerala State I: Parmelioid macro-lichens. J. Econ. Taxon. Bot. 34, 890–897.

Bjelland, T., Thorseth, I.H., 2002. Comparative studies of the lichen-rock interface of four lichens in Vingen, western Norway. Chem. Geol. 192, 81–98. https://doi.org/10.1016/S0009-2541(02)00193-6

Boqueras, M., Llimona, X., 2003. The genus *Pertusaria* (lichenised ascomycotina) on the Iberian Peninsula and Balearic

Islands. I. Subgenus Pertusaria. Mycotaxon 88, 471–492.

Brand, A.M., Van Den Boom, P.P.G., Sérusiaux, E., 2014. Unveiling a surprising diversity in the lichen genus *Micarea* (Pilocarpaceae) in Réunion (Mascarenes archipelago, Indian Ocean). Lichenologist 46, 413–439. https://doi.org/10.1017/S0024282913000911

Brinker, S.R., Knudsen, K., 2019. The first confirmed report of Acarospora bullata from North America. Opusc. Philolichenum 18, 11–16.

Brodo, I.M., 1984. Lichenes *Canadenses Exsiccati*: Fascicle III. Bryologist 87, 97. https://doi.org/10.2307/3243115

Brodo, I.M., McCune, B., 2017. *Ochrolechia brodoi* , a New Lichen for North America from Alaska, with Updates to the Key of Corticolous North American Species . Evansia 34, 110–113. https://doi.org/10.1639/0747-9859-34.3.110

Bryan, A.J., Elix, J.A., 1976. 2-O-Acetyltenuiorin, a new tridepside from the lichen *Pseudocyphellaria australiensis*. Aust. J. Chem. 29, 1147–1151. https://doi.org/10.1071/CH9761147

Bungartz, F., Benatti, M.N., Spielmann, A.A., 2013. The genus *Bulbothrix* (Parmeliaceae, Lecanoromycetes) in the Galapagos Islands: A case study of superficially similar, but overlooked macrolichens. Bryologist 116, 358–372. https://doi.org/10.1639/0007-2745-116.4.358

Bungartz, F., Nash, T.H., 2004. The *Buellia aethalea*-group in the Greater Sonoran Desert Region with reference to similar species in North America. Bryologist 107, 441–458. https://doi.org/10.1639/0007-2745(2004)107[441:TBAITG]2.0.CO;2

Cáceres, M.E.D.S., Aptroot, A., Ertz, D., 2014. New species and interesting records of Arthoniales from the Amazon, Rondônia, Brazil. Lichenologist 46, 573–588. https://doi.org/10.1017/S0024282914000036

Calatayud, V., Rambold, G., 1998. Two new species of the lichen genus *Immersaria* (Porpidiaceae). Lichenologist 30, 231–244. https://doi.org/10.1006/lich.1997.0133

Canez, L., Marcelli, M.P., Elix, J.A., 2009. New Brazilian species of *Canoparmelia* with medullary olivetoric, anziaic and sekikaic complexes. Mycotaxon 110, 465–472. https://doi.org/10.5248/110.465

Casares-Porcel, M., Hafellner, J., Gutierrez-Carretero, L., 1996. Species of the genus *Lecidea* (Lecanorales) on gypsum in Spain. Lichenologist 28, 37–47. https://doi.org/10.1006/lich.1996.0004

Clerc, P., Truong, C., 2008. The non-sorediate and non-isidiate *Parmelina* species (lichenized ascomycetes, Parmeliaceae) in Switzerland - Parmelina atricha (NYL.) P. CLERC reinstated in the European lichen flora. Sauteria 15, 175–194.

Coppins, B.J., Fryday, A.M., 2006. New or previously misunderstood species of *Lithographa* and *Rimularia* (Agyriaceae) from the southern subpolar region and western Canada. Lichenologist 38, 93–107. https://doi.org/10.1017/S0024282906005512

Corbett, R.E., Cumming, S.D., 1971. Lichens and fungi. Part VII. Extractives from the lichen *Sticta* mougeotiana var. *dissecta* del. J. Chem. Soc. C Org. Chem. 955–960. https://doi.org/10.1039/J39710000955

Cornejo, C., Scheidegger, C., 2015. Multi-gene phylogeny of the genus *Lobaria*: Evidence of species-pair and allopatric cryptic speciation in east asia. Am. J. Bot. 102, 2058–2073. https://doi.org/10.3732/ajb.1500207

Cuellar, M., Quilhot, W., Rubio, C., Soto, C., Espinoza, L., Carrasco, H., 2008. Phenolics, depsides and triterpenes from the Chilean lichen *Pseudocyphellaria nudata* (Zahlbr.) D.J. Galloway. J. Chil. Chem. Soc. 53, 1624–1625. https://doi.org/10.4067/S0717-97072008000300017

Culberson, C.F., Hale, M.E., Tønsberg, T., Johnson, A., 1984. New Depsides from the Lichens *Dimelaena Oreina* and *Fuscidea Viridis* . Mycologia 76, 148–160. https://doi.org/10.1080/00275514.1984.12023819

Culberson, C.F., LaGreca, S., Johnson, A., Culberson, W.L., 1999. Trivaric acid, a new tridepside in the *Ramalina americana* chemotype complex (Lichenized ascomycota: Ramalinaceae). Bryologist 102, 595–601. https://doi.org/10.2307/3244249

Culberson, W.L., 1963. A Summary of the Lichen Genus *Haematomma* in North America. Bryologist 66, 224.

https://doi.org/10.2307/3240633

Culberson, W.L., 1961. The *Parmelia quercina* Group in North America. Am. J. Bot. 48, 168–174. https://doi.org/10.1002/j.1537-2197.1961.tb11621.x

Culberson, W.L., Culberson, C.F., 1982. Evolutionary Modification of Ecology in a Common Lichen Species. Syst. Bot. 7, 158. https://doi.org/10.2307/2418324

Culberson, W.L., Culberson, C.F., 1981. The Genera *Cetrariastrum* and *Concamerella* (Parmeliaceae): A Chemosystematic Synopsis. Bryologist 84, 273. https://doi.org/10.2307/3242843

Culberson, W.L., Culberson, C.F., 1956. The Systematics of the *Parmelia dubia* Group in North America. Am. J. Bot. 43, 678. https://doi.org/10.2307/2438833

Czarnota, P., 2015. Lecania cuprea and *Micarea pycnidiophora* (lichenized Ascomycota) new to Poland. Acta Soc. Bot. Pol. 84, 303–307. https://doi.org/10.5586/asbp.2015.014

Davydov, E., Tchabanenko, S., Makryi, T., Khanin, V., 2011. The second confirmed record of *Umbilicaria pulvinaria* (lichenized Ascomycota) since its original description in 1914. Turczaninowia 14.

Davydov, E.A., Printzen, C., 2012. Rare and noteworthy boreal lichens from the Altai Mountains (South Siberia, Russia). Bryologist 115, 61–73. https://doi.org/10.1639/0007-2745.115.1.61

Dey, J.P., 1978. Fruticose and Foliose Lichens of the High-Mountain Areas of the Southern Appalachians. Bryologist 81, 1. https://doi.org/10.2307/3242271

Din, L., Latiff, A., Said, M., Elix, J., 2008. Additional lichen records from Indonesia and Malaysia 6.Lichens from Maliau Basin, Sabah, Malaysia. Australas. Lichenol.

Din, L.B., Ismail, G., Elix, J.A., 1999. The lichens in Bario Highlands: Their natural occurence and secondary metabolites. ASEAN Rev. Biodivers. Environ. Conserv. 1–6.

Din, L.B., Zakaria, Z., Samsudin, M.W., Elix, J.A., 2010. Chemical profile of compounds from Lichens of Bukit Larut, Peninsular Malaysia. Sains Malaysiana 39, 901–908.

Divakar, P.K., Crespo, A., 2018. Molecular phylogeny uncovers an overlooked species in the macrolichen family Parmeliaceae (Ascomycota) from India. Cryptogam Biodivers. Assess. https://doi.org/10.21756/cab.esp4

Divakar, P.K., Upreti, D.K., 2005. A new species in *Melanohalea* (Parmeliaceae, Ascomycotina) and new lichen records from India. Lichenologist 37, 511–517. https://doi.org/10.1017/S0024282905015215

Divakar, P.K., Upreti, D.K., 2003. New species and new records of *Parmotrema* (Parmeliaceae) from India. Lichenologist 35, 21–26. https://doi.org/10.1006/lich.2002.0426

Dou, M.-Z., Zhao, X., Jia, Z.-F., 2021. *Trapelia calyciformis* sp. nov. from China . Mycotaxon 135, 817–823. https://doi.org/10.5248/135.817

Dymytrova, L. V., 2011. Notes on the genus *Scoliciosporum* (Lecanorales, Ascomycota) in ukraine. Polish Bot. J. 56, 61–75.

Egea, J.M., Torrente, P., 1992. Two New Species of *Lecanactis* from Baja California. Bryologist 95, 161. https://doi.org/10.2307/3243429

Egea, J.M., Torrente, P., Manrique, E., 1993. The *Lecanactis rumulosa* group (Opegraphaceae) in the Mediterranean region. Plant Syst. Evol. 187, 103–114. https://doi.org/10.1007/BF00994093

Eigler, G., Poelt, J., 1965. Lichen substances and systematics of the lobate types of the genus *Lecanora* in the Arctic. Oesterreichisches Bot. Wochenblatt 112, 285–94.

Ekman, S., 1994. *Biatora Meiocarpa* (Nyl.) Arnold, A Misunderstood Species. Lichenol. 26, 31–37. https://doi.org/10.1006/lich.1994.1002

Ekman, S., 1993. A taxonomic study of *Ropalospora chlorantha*, and a comparison between *Ropalospora* and *Fuscidea*,

Bryologist. https://doi.org/10.2307/3243989

Eliasaro, S., Adler, M.T., 2000. The species of *Canomaculina*, *Myelochroa*, *Parmeunella*, and *Parmeunopsis* (Parmeliaceae, Lichenized Ascomycotina) from the" segundo planalto" in the state of. Acta Bot. Brasilica 14, 141–149.

Eliasaro, S., Donha, C.G., 2003. The genera *Canomaculina* and *Parmotrema* (Parmeliaceae, Lichenized Ascomycota) in Curitiba, Paraná State, Brazil. Rev. Bras. Botânica 26. https://doi.org/10.1590/s0100-84042003000200012

Elix, J.A., 2012. Four new species and a new record of *Tephromela* (lichenized Ascomycota) from Australia. Australas Lichenol 71, 3–11.

Elix, J.A., 2006. New species of *Xanthoparmelia* (Lichenized Ascomycota, Parmeliaceae) from southern and Western Australia. J. Hattori Bot. Lab. 635–649. https://doi.org/10.18968/jhbl.100.0_635

Elix, J.A., 2004. New species and new records of *Xanthoparmelia* (lichenized Ascomycota, Parmeliaceae) from eastern Australia. Lichenologist 36, 277–287. https://doi.org/10.1017/S0024282904014410

Elix, J.A., Barbero, M., Giralt, M., Lumbsch, H.T., McCaffery, L.F., 1995. 2″-*O*-methylgyrophoric acid, a new lichen tridepside. Aust. J. Chem. 48, 1761–1765. https://doi.org/10.1071/CH9951761

Elix, J.A., Engkaninan, U., 1976. 4, 5-Di-*O*-methylhiascic acid, a new tridepside from the lichens *Parmelia pseudofatiscens* and *Parmelia horrescens*. Aust. J. Chem. 29, 2701–2705. https://doi.org/10.1071/CH9762701

Elix, J.A., Jayanthi, V.K., 1981. 3-Methoxy-2, 4-di-*O*-methylgyrophoric Acid: A Novel Tridepside from the Lichen *Parmelia subfatiscens*. Aust. J. Chem. 34, 1153–1156. https://doi.org/10.1071/CH9811153

Elix, J.A., Jayanthi, V.K., 1977. 5-*O*-Methylhiascic acid, a new tridepside from Australian lichens. Aust. J. Chem. 30, 2695–2704. https://doi.org/10.1071/CH9772695

Elix, J.A., Jayanthi, V.K., Leznoff, C.C., 1981. 2, 4-Di-*O*-methylgyrophoric acid and 2, 4, 5-Tri-*O*-methylhiascic acid. new tridepsides from *Parmelia damaziana*. Aust. J. Chem. 34, 1757–1761. https://doi.org/10.1071/CH9811757

Elix, J.A., Jayanthi, V.K., Wardlaw, J.H., 1989a. 2-*O*-Methylhiascic acid, a new tridepside in the lichen *Parmelinopsis neodamaziana*. Aust. J. Chem. 42, 1423–1426. https://doi.org/10.1071/CH9891423

Elix, J.A., Jin, Y., Adler, M.T., 1989b. 3-Hydroxyurnbilicaric Acid and 3-Methoxyumbilicaric Acid, New Tridepsides From the Lichen *Parmelinopsis Bonariensis*. Aust. J. Chem. 42, 765–770. https://doi.org/10.1071/CH9890765

Elix, J.A., Lajide, L., 1981. 2'-*O*-methyltenuiorin, 2"-*O*-methyltenuiorin and 2', 2"-Di-*O*-methyltenuiorin. three new tridepsides from the lichen *Pseudocyphellaria faveolata*. Aust. J. Chem. 34, 2005–2011. https://doi.org/10.1071/CH9812005

Elix, J.A., Nash, T.H., 1995. New Species of Parmeliaceae (lichenized Ascomycotina) from South America. Trop. Bryol. 11, 161–167.

Elix, J.A., Wardlaw, J.H., 1997. New depsides from the Lichen *Neofuscelia depsidella*. Aust. J. Chem. 50, 1145–1150. https://doi.org/10.1071/C97091

Elix, J.A., Wirtz, N., Lumbsch, H.T., 2007. Studies on the chemistry of some *Usnea* species of the *Neuropogon* group (Lecanorales, Ascomycota). Nov. Hedwigia 85, 491–501. https://doi.org/10.1127/0029-5035/2007/0085-0491

Elix, J.A., Yu, J., Tonsberg, T., 1991. 4-*O*-methylhiascic acid and 5-*O*-Acetyl-4-*O*-methylhiascic acid, two new lichen tridepsides. Aust. J. Chem. 44, 157–163. https://doi.org/10.1071/CH9910157

Ertz, D., Fryday, A.M., Schmitt, I., Charrier, M., Dudek, M., Kukwa, M., 2016. *Ochrolechia kerguelensis* sp. Nov. from the Southern Hemisphere and O. Antarctica reinstated from the synonymy of *O. parella*. Phytotaxa 280, 129–140. https://doi.org/10.11646/phytotaxa.280.2.3

Ertz, D., Sérusiaux, E., 2009. A new species of *Lecanactis* (Arthoniales, Roccellaceae) from Madagascar. Lichenologist 41, 147–150. https://doi.org/10.1017/S0024282909008287

Esslinger, T.L., 1973. Nomenclatural Notes on Some Members of *Parmelia* Section *Melanoparmelia*. Bryologist 76, 306.

https://doi.org/10.2307/3241337

Esslinger, T.L., Barbero, M., Llimona, X., 1993. *Neofuscelia halei* sp. nov. (Lichen-Forming Ascomycota) from Spain and the Canary Islands. Bryologist 96, 355. https://doi.org/10.2307/3243866

Etayo, J., Giralt, M., Elix, J.A., 2010. *Buellia rhizocarpica*, a new corticolous species from Mexico. Lichenologist 42, 723–726. https://doi.org/10.1017/S0024282910000423

Fahselt, D., Alstrup, V., 1997. High performance liquid chromatography of phenolics in recent and subfossil lichens. Can. J. Bot. 75, 1148–1154. https://doi.org/10.1139/b97-826

Flakus, A., Kukwa, M., 2011. *Lepraria maderensis* Kukwa & Flakus, a new lichen species containing gyrophoric and lecanoric acids. Nov. Hedwigia 92, 95–99. https://doi.org/10.1127/0029-5035/2011/0092-0095

Flakus, A., Saavedra, P.R., Kukwa, M., 2012. A new species and new combinations and records of *Hypotrachyna* and *Remototrachyna* from Bolivia. Mycotaxon 119, 157–166. https://doi.org/10.5248/119.157

Frisch, A., Elix, J.A., Thor, G., 2010. *Herpothallon biacidum*, a new lichen species from tropical Australia. Lichenologist 42, 285–289. https://doi.org/10.1017/S0024282909990697

Frisch, A., Rudolphi, J., Thor, G., 2014a. *Herpothallon inopinatum* (Arthoniaceae), a new lichen species from Mexico. Ann. Bot. Fenn. 51, 63–68. https://doi.org/10.5735/085.051.0108

Frisch, A., Thor, G., 2010. *Crypthonia*, a new genus of byssoid Arthoniaceae (lichenised Ascomycota). Mycol. Prog. 9, 281–303. https://doi.org/10.1007/s11557-009-0639-8

Frisch, A., Thor, G., Ertz, D., Grube, M., 2014b. The Arthonialean challenge: Restructuring Arthoniaceae. Taxon 63, 727–744. https://doi.org/10.12705/634.20

Fritis, M.C., Lagos, C.R., Sobarzo, N.Q., Venegas, I.M., Sánchez, C.S., Altamirano, H.C., Catalán, L.E., Palma, W.Q., 2013. Depsides and triterpenes in *Pseudocyphellaria coriifolia* (lichens) and biological activity against *Trypanosoma cruzi*. Nat. Prod. Res. 27, 1607–1610. https://doi.org/10.1080/14786419.2012.740033

Fryday, A.M., 2019. Eleven new species of crustose lichenized fungi from the Falkland Islands (Islas Malvinas). Lichenologist 51, 235–267. https://doi.org/10.1017/S0024282919000185

Fryday, A.M., Coppins, B.J., 1997. Keys to sterile, crustose saxicolous and terricolous lichens occurring in the British Isles. Lichenologist 29, 301–332. https://doi.org/10.1006/lich.1997.0080

Fryday, A.M., Van Den Boom, P.P.G., 2019. *Lecidea phaeophysata*: A new saxicolous lichen species from western and southern Europe with a key to saxicolous lecideoid lichens present on Atlantic coasts. Lichenologist 51, 193–204. https://doi.org/10.1017/S0024282919000070

Gadea, A., Le Pogam, P., Biver, G., Boustie, J., Le Lamer, A.C., Le Dévéhat, F., Charrier, M., 2017. Which specialized metabolites does the native subantarctic gastropod notodiscus hookeri extract from the consumption of the lichens *Usnea taylorii* and *Pseudocyphellaria crocata*? Molecules 22, 425. https://doi.org/10.3390/molecules22030425

Galloway, D.J., 2010. Additions to the *Placopsis mycobiota* (Trapeliaceae, Ascomycota) of southern South America, with notes on new records (including *Aspiciliopsis macrophthalma*), and a revised regional key to the species. Lichenologist 42, 727–737. https://doi.org/10.1017/S0024282910000460

Galloway, D.J., 2004. New lichen taxa and names in the New Zealand mycobiota. New Zeal. J. Bot. 42, 105–120. https://doi.org/10.1080/0028825X.2004.9512893

Geyer, M., Feuerer, T., Feige, G.B., 1984. Chemistry and systematics of the genus Rhizocarpon: high performance liquid chromatography (HPLC) of the secondary lichen products in the *Rhizocarpon superficiale* group. I. Plant Syst. Evol. 145, 41–54.

Giordani, P., Benesperi, R., Rellini, I., Frati, L., Brunialti, G., Paoli, L., Isocrono, D., Elix, J.A., 2003. The lichen genus *Neofuscelia* (Ascomycota, Parmeliaceae) in Italy. Lichenologist 35, 377–385. https://doi.org/10.1016/j.lichenologist.2003.09.001

Giralt, M., Barbero, M., Van den Boom, P.P.G., 1996. *Rinodina algarvensis*, a new saxicolous sorediate species from Portugal containing the stictic acid complex. Lichenologist 28, 1–8. https://doi.org/10.1006/lich.1996.0001

Giralt, M., Bungartz, F., Elix, J.A., 2011. The identity of *Buellia sequax*. Mycol. Prog. 10, 115–119. https://doi.org/10.1007/s11557-010-0695-0

Giralt, M., Elix, J.A., 2010. New morphological and chemical data for *Buellia imshaugii*. Lichenologist 42, 763–765. https://doi.org/10.1017/S0024282910000472

Giralt, M., van den Boom, P.P.G., Mayrhofer, H., Elix, J.A., 2014. Three new species of crustose Physciaceae from Guatemala, with notes on some additional species. Phytotaxa 164, 79–90. https://doi.org/10.11646/phytotaxa.164.2.2

Goffinet, B., Hastings, R.I., 1995. Two New Sorediate Taxa Of *Peltigera*. Lichenol. 27, 43–58. https://doi.org/10.1006/lich.1995.0004

Goffinet, B., Miadlikowska, J., 1999. *Peltigera phyllidiosa* (Peltigeraceae, ascomycotina), a new species from the Southern Appalachians corroborated by its sequences. Lichenologist 31, 247–256. https://doi.org/10.1006/lich.1998.0201

González, A.G., Barrera, J.B., Marante, F.J.T., Castellano, A.G., 2002. The Chemistry and Allelopathic Effects of Phenolic Compounds from the Lichen *Evernia prunastri* (L.) Ach., in: Natural Products in the New Millennium: Prospects and Industrial Application. Springer Netherlands, pp. 195–210. https://doi.org/10.1007/978-94-015-9876-7_20

Gowan, S.P., 1989. A Character Analysis of the Secondary Products of the Porpidiaceae (Lichenized Ascomycotina). Syst. Bot. 14, 77. https://doi.org/10.2307/2419052

Goward, T., Goffinet, B., 2000. *Peltigera chionophila*, a new lichen (Ascomycetes) from the western cordillera of North America. Bryologist 103, 493–498. https://doi.org/10.1639/0007-2745(2000)103[0493:PCANLA]2.0.CO;2

Goward, T., Goffinet, B., Vitikainen, O., 1995. Synopsis of the genus *Peltigera* (lichenized Ascomycetes) in British Columbia, with a key to the North American species. Can. J. Bot. 73, 91–111. https://doi.org/10.1139/b95-012

Gruezo, W.S., 1983. *Lobaria clemensiae* Vain. (Lobariaceae, Lichenes) on Halmabeira Island, Indonesia. Gard. Bull. Singapore.

Hale, M.E., 1971. Two Species of *Parmelia* New to North America. Bryologist 74, 44. https://doi.org/10.2307/3241757

Hamada, N., Tanahashi, T., Goldsmith, S., Nash, T.H., 1997. Induction of secondary products in isolated mycobionts from North American lichens. Symbiosis 23, 219–224.

Henssen, A., 1991. *Omphalodiella patagonica*, a new peltate lichen genus and species from south America. Lichenol. 23, 333–342. https://doi.org/10.1017/S002428299100049X

Holtan-Hartwig, J., 1988. Two new species of *Peltigera*. Lichenol. 20, 11–17. https://doi.org/10.1017/S0024282988000040

Holzmann, G., Leuckert, C., 1990. Applications of negative fast atom bombardment and MS/MS to screening of lichen compounds. Phytochemistry 29, 2277–2283. https://doi.org/10.1016/0031-9422(90)83052-3

Huneck, S., 1974. Lichen Components .89. Secondary Substances of Some Lichens. Phytochemistry 11, 0–1493.

Huneck, S., Schreiber, K., 1974. Lichen compounds. 102. Secondary compounds of several European and Indian lichens. Phytochemistry 13, 2315–16.

Huneck, S., Schreiber, K., Sundholm, G., 1980. Ovosäure, ein neues tridepsid aus der flechte *Parmelia substygia*. Phytochemistry 19, 885–887. https://doi.org/10.1016/0031-9422(80)85131-4

Jagadeesh Ram, T.A.M., 2014. The genus *Herpothallon* (Arthoniaceae) in the Andaman Islands, India. Lichenologist 46, 39–49. https://doi.org/10.1017/S0024282913000571

Jagadeesh Ram, T.A.M., Sinha, G.P., 2016. A world key to *Cryptothecia* and *Myriostigma* (Arthoniaceae), with new species and new records from the Andaman and Nicobar Islands, India. Phytotaxa 266, 103–114. https://doi.org/10.11646/phytotaxa.266.2.4

Jagadeesh Ram, T.A.M., Sinha, G.P., 2009. New species and new records of *Herpothallon* (lichenized ascomycota) from India. Mycotaxon 110, 37–42. https://doi.org/10.5248/110.37

Jannah, M., Afifah, N., Hariri, M.R., Rahmawati, A., Wulansari, T.Y.I., 2020. Study of lichen (*Usnea* spp.) as a traditional medicine in Bogor, West Java. Berk. Penelit. Hayati 26, 32–38. https://doi.org/10.23869/bphjbr.26.1.20206

Jayalal, U., Joshi, S., Oh, S.O., Park, J.S., Koh, Y.J., Hur, J.-S., 2013. Notes on the lichen genus *Hypotrachyna* (Parmeliaceae) from South Korea. Mycobiology 41, 13–17. https://doi.org/10.5941/MYCO.2013.41.1.13

Jordan, W.P., 1972. Erumpent Cephalodia, an Apparent Case of Phycobial Influence on Lichen Morphology. J. Phycol. 8, 112–117. https://doi.org/10.1111/j.1529-8817.1972.tb04008.x

Jørgensen, P.M., 1993. *Pseudohepatica*, a Remarkable New Lichen Genus from Venezuela. Bryologist 96, 435. https://doi.org/10.2307/3243874

Joshi, Y., Jagadeesh Ram, T.A.M., Sinha, G.P., 2012. *Caloplaca gyrophorica* (lichenized Ascomycota), a new saxicolous lichen species from India. Mycotaxon 122, 303–306. https://doi.org/10.5248/122.303

Joshi, Y., Koh, Y.J., Hur, J.-S., 2010a. Three New Records of Lichen Genus *Rhizocarpon* from South Korea . Mycobiology 38, 219. https://doi.org/10.4489/myco.2010.38.3.219

Joshi, Y., Nguyen, T.T., Wang, X.Y., Lökös, L., Koh, Y.J., Hur, J.-S., 2011. Contribution to the lichen mycota of South Korea. Mycotaxon 116, 61–74. https://doi.org/10.5248/116.61

Joshi, Y., Wang, X.Y., Yamamoto, Y., Koh, Y.J., Hur, J.-S., 2010b. A first modern contribution to Caloplaca biodiversity in South Korea: Two new species and some new country records. Lichenologist 42, 715–722. https://doi.org/10.1017/S0024282910000368

Jungbluth, P., Marcelli, M.P., Elix, J.A., 2008. Five new species of Bulbothrix (Parmeliaceae) from cerrado vegetation in São Paulo State, Brazil. Mycotaxon 104, 51–63.

Kantvilas, G., 2018. *Micarea kartana* sp. nov.(lichenised Ascomycetes) from Kangaroo Island, South Australia. Jstor 31, 55–58.

Kantvilas, G., 2016. A synopsis and key for the lichen genus *Caloplaca* (Teloschistaceae) on Kangaroo Island , with the description of two new species. J. Adel. Bot. Gard. 29, 53–69.

Kantvilas, G., 1999. A new species of *Schaereria* from Tasmania. Lichenologist 31, 231–238. https://doi.org/10.1006/lich.1998.0202

Kantvilas, G., Coppins, B.J., 2019. Studies on *Micarea* in Australasia II. A synopsis of the genus in Tasmania, with the description of ten new species. Lichenol. 51, 431–481. https://doi.org/10.1017/s0024282919000343

Kantvilas, G., Elix, J.A., 2007. Additions to the lichen family Agyriaceae Corda from Tasmania. Bibl. Lichenol. 95, 317–333.

Kantvilas, G., Leavitt, S.D., Elix, J.A., Lumbsch, H.T., 2014. Additions to the genus *Trapelia* (Trapeliaceae: Lichenised Ascomycetes). Aust. Syst. Bot. 27, 395–402. https://doi.org/10.1071/SB14037

Kantvilas, G., McCarthy, P.M., Stuckey, B., 2008. A remarkable new species of *Rimularia* Nyl . (lichenized fungi : Trapeliaceae) from tropical Australia. Autrobaileya 7, 659–663.

Kantvilas, G., Stajsic, V., McCarthy, P.M., 2020. A new combination in *Angiactis* (lichenised Ascomycetes: Roccellaceae). Muelleria 38, 71–75.

Knudsen, K., 2008. *Acarospora Fuscescens* (Acarosporaceae), a Little Known Species of Western North America. Evansia 25, 82–84. https://doi.org/10.1639/0747-9859-25.4.82

Knudsen, K., Elix, J.A., Lendemer, J.C., 2006. Two new records of *Lepraria* from California. Bull. Calif. Lichen Soc. 13, 3–5.

Knudsen, K., Flakus, A., 2009. *Acarospora ramosa* (Acarosporaceae), A new effigurate yellow species from South America.

Nov. Hedwigia 89, 349–353. https://doi.org/10.1127/0029-5035/2009/0089-0349

Knudsen, K., Kocourková, J., 2018. Two new calciphytes from Western North America, *Acarospora brucei* and *Acarospora erratica* (acarosporaceae). Opusc. Philolichenum 17, 342–350.

Knudsen, K., Lendemer, J.C., 2009. Two new species of *Lecanora* with gyrophoric acid from North America. Opusc. Philolichenum 7, 21–28.

Knudsen, K., Lendemer, J.C., Elix, J.A., 2011. *Lecanora peninsularis* (Lecanoraceae, lichenized Ascomyctes), a second new species with gyrophoric acid from California. Nov. Hedwigia 92, 101–105. https://doi.org/10.1127/0029-5035/2011/0092-0101

Knudsen, K., Morse, C.A., 2009. *Acarospora nicolai* (Acarosporaceae), a rediscovered species. Bryologist 112, 147–151. https://doi.org/10.1639/0007-2745-112.1.147

Knudsen, K., Reeb, V., Westberg, M., Srikantha, R., Bhattacharya, D., 2010. *Acarospora rosulata* in Europe, North America and Asia. Lichenologist 42, 291–296. https://doi.org/10.1017/S0024282909990715

Kondratyuk, S.Y., Lokos, L., Halda, J.P., Upreti, D.K., Mishra, G.K., Haji Moniri, M., Farkas, E., Park, J.S., Lee, B.G., Liu, D., Woo, J.J., Jayalal, R.G.U., Oh, S.O., Hur, J.-S., 2016. New and noteworthy lichen-forming and lichenicolous fungi 5. Acta Bot. Hung. 59, 319–396. https://doi.org/10.1556/ABot.58.2016.3-4.7

Kondratyuk, S.Y., Lökös, L., Kim, J.A., Kondratiuk, A.S., Jeong, M.H., Jang, S.H., Oh, S.O., Hur, J.-S., 2015. Three new monotypic genera of the caloplacoid lichens (Teloschistaceae, Lichen-Forming Ascomycetes). Mycobiology 43, 195–202. https://doi.org/10.5941/MYCO.2015.43.3.195

Kondratyuk, S.Y., Tschabanenko, S.I., Elix, J.A., Oh, S.O., Thell, A., Hur, J.-S., 2013. *Nipponoparmelia perplicata* sp. nov. (Parmeliaceae, Ascomycota) from eastern Asia. Mycotaxon 126, 37–44. https://doi.org/10.5248/126.37

Kukwa, M., 2009. *Ochrolechia aegaea* and *O. alaskana*, two species with gyrophoric and variolaric acids in the cortex. Graphis Scripta 21, 42-48.

Kukwa, M., Łubek, A., Szymczyk, R., Zalewska, A., 2012. Seven lichen species new to Poland. Mycotaxon 120, 105–118. https://doi.org/10.5248/120.105

Kusmoro, J., Noer, I.S., Jatnika, M.F., Permatasari, R.E., Partasasmita, R., 2018. Lichen diversity in geothermal area of Kamojang, Bandung, West Java, Indonesia and its potential for medicines and dyes. Biodiversitas 19, 2335–2343. https://doi.org/10.13057/biodiv/d190643

Leavitt, S.D., Fankhauser, J.D., Leavitt, D.H., Porter, L.D., Johnson, L.A., St. Clair, L.L., 2011. Complex patterns of speciation in cosmopolitan “rock posy” lichens - Discovering and delimiting cryptic fungal species in the lichen-forming *Rhizoplaca melanophthalma* species-complex (Lecanoraceae, Ascomycota). Mol. Phylogenet. Evol. 59, 587–602. https://doi.org/10.1016/j.ympev.2011.03.020

Lendemer, J.C., Allen, J.L., 2020. A revision of *Hypotrachyna* subgenus *Parmelinopsis* (Parmeliaceae) in eastern North America. Bryologist 123, 265–332. https://doi.org/10.1639/0007-2745-123.2.265

Louwhoff, S.H.J.J., Elix, J.A., 2000. The lichens of Rarotonga, Cook Islands, South Pacific Ocean II: Parmeliaceae. Lichenologist 32, 49–55.

Lumbsch, H.T., Elix, J.A., 1989. Taxonomy of some *Diploschistes* spp. (lichenized ascomycetes, Thelotremataceae) containing gyrophoric acid. Plant Syst. Evol. 167, 195–199. https://doi.org/10.1007/BF00936406

Lumbsch, H.T., Kashiwadani, H., Streimann, H., 1993. A remarkable new species in the lichen genus *Placopsis* from Papua New Guinea (lichenized ascomycetes, Agyriaceae). Plant Syst. Evol. 185, 285–292. https://doi.org/10.1007/BF00937664

Lumbsch, H.T., Messuti, M.I., Nash, T.H., 2003. *Ochrolechia splendens* (Pertusariaceae), a new species from south-western North America. Lichenologist 35, 387–391. https://doi.org/10.1016/S0024-2829(03)00057-4

Lumbsch, H.T., Nash, T.H., Messuti, M.I., 1999. A revision of *Pertusaria* species with hyaline ascospores in southwestern

North America (Pertusariales, Ascomycotina). Bryologist 102, 215–239. https://doi.org/10.2307/3244362

Lumbsch, H.T., Schmitt, I., Döring, H., Wedin, M., 2001. ITS sequence data suggest variability of ascus types and support ontogenetic characters as phylogenetic discriminators in the Agyriales (Ascomycota). Mycol. Res. 105, 265–274. https://doi.org/10.1017/S0953756201003483

Lunke, T., Lumbsch, H.T., Feige, G.B., 1996. Anatomical and ontogenetic studies on the lichen family Schaereriaceae (Agyriineae, Lecanorales). Bryologist 99, 53–63. https://doi.org/10.2307/3244438

Maass, W.S.G., 1975a. Lichen Substances VII. Identification of Orsellinate Derivatives from *Lobaria linita*. Bryologist 78, 178. https://doi.org/10.2307/3242048

Maass, W.S.G., 1975b. The phenolic constituents of *Peltigera aphthosa*. Phytochemistry 14, 2487–2489. https://doi.org/10.1016/0031-9422(75)80370-0

Maass, W.S.G., 1975c. Lichen substances. V. Methylated derivatives of orsellinic acid, lecanoric acid, and gyrophoric acid from *Pseudocyphellaria crocata* . Can. J. Bot. 53, 1031–1039. https://doi.org/10.1139/b75-121

Maass, W.S.G., 1975d. Lichen Substances VIII. Phenolic Constituents of *Pseudocyphellaria quercifolia*. Bryologist 78, 183. https://doi.org/10.2307/3242049

Magain, N., Goffinet, B., Simon, A., Seelan, J.S.S., Medeiros, I.D., Lutzoni, F., Miadlikowska, J., 2020. *Peltigera serusiauxii* (Lecanoromycetes, Ascomycota), a new species from Papua New Guinea and Malaysia. Plant Fungal Syst. 139–146. https://doi.org/10.35535/pfsyst-2020-0009

Magain, N., Sérusiaux, E., Zhurbenko, M.P., Lutzoni, F., Miadlikowska, J., 2016. Disentangling the *Peltigera polydactylon* Species Complex by Recognizing Two New Taxa, *P. polydactylon* subsp. udeghe and *P. seneca* . Herzogia 29, 514–528. https://doi.org/10.13158/heia.29.2.2016.514

Maier, M.S., González Marimon, D.I., Stortz, C.A., Adler, M.T., 1999. A revised structure for (-)-dihydropertusaric acid, a γ-butyrolactone acid from the lichen *Punctelia microsticta*. J. Nat. Prod. 62, 1565–1567. https://doi.org/10.1021/np990110n

Marcano, V., Mohali, S., Palacios-Prü, E., 1997. Morphological and chemical observations on *Peltigera vainioi* Gyelnik (Lichenized Ascomycetes, Peltigeraceae) from South America. Lichens 1, 1–10.

Marcano, V., Mohali, S., Palacios-Prü, E., Morales Méndez, A., 1996. The lichen genus *Bulbothricella*, a new segregate in the Parmeliaceae from Venezuela. Lichenologist 28, 421–430. https://doi.org/10.1006/lich.1996.0040

Marcano, V., Palacios-Prü, E., Morales, A., 2000. *Pseudohepatica duidensis*, a new lichen from the venezuelan Amazonas. Trop. Bryol. 18, 203–212.

Matwiejuk, A., 2010. Lichen secondary metabolites occurring in lichens of the genus *Rhizocarpon* Ramond ex DC.(Rhizacarpaceae, lichenized Ascomcota) in Poland. Bot. – Steciana 14, 107–113.

Matzer, M., Mayrhofer, H., Elix, J.A., 1997. *Australiaena streimannii*, a new genus and species in the Physciaceae from tropical Australasia. Lichenologist 29, 35–44. https://doi.org/10.1006/lich.1996.0058

Matzer, M., Mayrhofer, H., Sattler, J., Clerc, P., 1994. *Rinodina canariensis* (lichenized Ascomycetes, Physciaceae), a new species parasitic on crustose lichens in Macaronesia and the Mediterranean region. Nord. J. Bot. 14, 105–111. https://doi.org/10.1111/j.1756-1051.1994.tb00576.x

McCune, B., Camacho, F., Ponzetti, J., 2002. Three new species of *Trapeliopsis* on soil in western North America. Bryologist 105, 78–85. https://doi.org/10.1639/0007-2745(2002)105[0078:TNSOTO]2.0.CO;2

McCune, B., Curtis, M.J., Di Meglio, J., 2017. New taxa and a case of ephemeral spore production in Lecideaceae from western North America. Bryologist 120, 115–124. https://doi.org/10.1639/0007-2745-120.2.115

Messuti, M.I., De La Rosa, I.N., 2007. *Byssoloma rubromarginatum* (Pilocarpaceae: Ascomycota), a new corticolous species from Nothofagus forests in Argentina. Mycol. Prog. 6, 235–238. https://doi.org/10.1007/s11557-007-0542-0

Messuti, M.I., Passo, A., Scervino, J.M., Vidal-Russell, R., 2016. The species pair *Pseudocyphellaria pilosella-piloselloides*

(lichenized Ascomycota: Lobariaceae) is a single species. Lichenologist 48, 141–146. https://doi.org/10.1017/S0024282915000511

Miralles, I., Edwards, H.G.M., Domingo, F., Jorge-Villar, S.E., 2015. Lichens around the world: A comprehensive study of lichen survival biostrategies detected by Raman spectroscopy. Anal. Methods 7, 6856–6868. https://doi.org/10.1039/c5ay00655d

Mirando, M., Fahselt, D., 1978. The effect of thallus age and drying procedure on extractable lichen substances. Can. J. Bot. 56, 1499–1504. https://doi.org/10.1139/b78-175

Moncada, B., Lücking, R., Betancourt-Macuase, L., 2013. Phylogeny of the Lobariaceae (lichenized Ascomycota: Peltigerales), with a reappraisal of the genus Lobariella. Lichenologist 45, 203–263. https://doi.org/10.1017/S0024282912000825

Moore, B., 1969. *Lobaria lobulifera*, a new species from the southeastern United States. Bryologist 72, 404-406. https://doi.org/10.2307/3241699

Morales Méndez, A., Marcano, V., Galiz, L., Mohali, S., Palacios-Prü, E., 1995. *Bulbothrix amazonensis* sp. nov., a new species of Parmeliaceae (Lecanorales) from Venezuelan Amazonia. Flecht. Follmann. Contrib. to Lichenol. Honour Gerhard Follmann (eds. FJA Daniels, M. Schulz y J. Peine) 281–286.

Myllys, L., Lindgren, H., Aikio, S., Häkkinen, L., Högnabba, F., 2016. Chemical diversity and ecology of the genus *Bryoria* section *Implexae* (Parmeliaceae) in Finland. Bryologist 119, 29–38. https://doi.org/10.1639/0007-2745-119.1.029

Narui, T., Culberson, C.F., Culberson, W.L., Johnson, A., Shibata, S., 1996a. A contribution to the chemistry of the lichen family Umbilicariaceae (Ascomycotina). Bryologist 99, 199–211. https://doi.org/10.2307/3244550

Narui, T., Sawada, K., Takatsuki, S., Okuyama, T., Culberson, C.F., Culberson, W.L., Shibata, S., 1998. NMR assignments of depsides and tridepsides of the lichen family Umbilicariaceae. Phytochemistry 48, 815–822. https://doi.org/10.1016/S0031-9422(97)00958-8

Narui, T., Takatsuki, S., Sawada, K., Okuyama, T., Culberson, C.F., Culberson, W.L., Shibata, S., 1996b. Lasallic acid, a tridepside from the lichen, *Lasallia asiae-orientalis*. Phytochemistry 42, 839–842. https://doi.org/10.1016/0031-9422(95)00960-4

Ngoc Tuan, N., Kuo, P.-C., Trung Hieu, T., Tuong Vi, L.N., Tong Hung, Q., Dunge, L.T., Duy Trinh, N., Quang Trung, N., Cuu Khoa, N., Viet Hai, H., Thang, T.D., 2019. A new triterpenoid and other compounds from lichens *Cryptothecia faveomaculata* Makhija & Patw . Nat. Prod. Res. 1–8. https://doi.org/10.1080/14786419.2019.1648466

Nicollier, G., Rebetez, M., Tabacchi, R., 1979. Identification et synthèse de nouveaux depsides isolés de la mousse de chêne (*Evernia Prunastri* (L.) ACH.). 4e Communication. Helv. Chim. Acta 62, 711–717. https://doi.org/10.1002/hlca.19790620310

Niu, D.-L., Harada, H., Wang, L.-S., Zhang, Y.-J., Yang, C.R., 2011a. Chemotaxonomic study of the *Lethariella cladonioides* complex (lichenized Ascomycota, Parmeliaceae). Lichenologist 43, 213–223. https://doi.org/10.1017/S0024282911000119

Niu, D.-L., Wang, L.-S., Zhang, Y.-J., Zang, C., 2011b. Chemical constituents of Acroscyphus sphaerophoroides. Plant Sci. J.

Nordin, A., 2004. Three species new to Sweden in material collected by Du Rietz. Graph. Scr. 16, 20–22.

Nurtai, L., Knudsen, K., Abbas, A., 2018. A preliminary study of the yellow *Acarospora* of China. Mycotaxon 133, 681–691. https://doi.org/10.5248/133.681

Nurtai, L., Knudsen, K., Abbas, A., 2017. A new species of the *Acarospora strigata* group (Acarosporaceae) from China . Bryologist 120, 382–387. https://doi.org/10.1639/0007-2745-120.4.382

Obermayer, W., 2001. on the Identity of Lethariella Sinensis Wei & Jiang , With New Reports of Tibetan *Lethariella* Species *. Bibl. Lichenol. 78, 321–326.

Obermayer, W., Blaha, J., Mayrhofer, H., 2004. Buellia centralis and chemotypes of *Dimelaena oreina* in Tibet and other Central-Asian regions. Symbolae Botanicae Upsalienses 34, 327-342.

Okuyama, E., Hossain, C.F., Yamazaki, M., 1991. Monoamine oxidase inhibitors from a lichen, *Solorina rocea* (L.) Ach. Japanese J. Pharmacogn. 45, 159–162.

Orange, A., 2018. A new species-level taxonomy for *Trapelia* (Trapeliaceae, Ostropomycetidae) with special reference to Great Britain and the Falkland Islands. Lichenologist 50, 3–42. https://doi.org/10.1017/S0024282917000639

Øvstedal, D.O., 1988. 1.2 On the Variation of *Ochrolechia parella* in the Western Antarctic and Subantarctic Area. Polarforschung 58, 77–81.

Pandit, G., 2014. *Immersaria* and *Koerberiella*, two new generic records to India. Curr. Res. Environ. Appl. Mycol. 4, 137–140. https://doi.org/10.5943/cream/4/1/12

Pandit, G., Sharma, B., 2012. New records in the lichen family Lobariaceae from the Western Ghats of India. Mycosphere 3, 430–435. https://doi.org/10.5943/mycosphere/3/4/6

Park, J.S., Oh, S.O., Woo, J.J., Liu, D., Park, S.Y., Hur, J.-S., 2019. First report of the lichen *Ochrolechia akagiensis* (Ochrolechiaceae, Ascomycota) in Korea. Korean J. Mycol. 47, 95–104. https://doi.org/10.4489/KJM.20190012

Park, Y.S., 1990. The Macrolichen Flora of South Korea. Bryologist 93, 105. https://doi.org/10.2307/3243619

Perez-Vargas, I., Padrón, C.H., Pérez De Paz, P.L., Elix, J.A., 2010. *Xanthoparmelia teydea*, a new brown *Xanthoparmelia* (Parmeliaceae) from the Canary Islands. Bryologist 113, 51–54. https://doi.org/10.1639/0007-2745-113.1.51

Piovano, M., Chamy, M.C., Garbarino, J.A., Lopez, C., Quilhot, W., 1995. Studies on Chilean lichens. XXV. Secondary products from *Leioderma pycnophorum* NyL. (Pannariaceae). Bol. la Soc. Chil. Quim. 40, 437–9.

Poelt, J., Černohorský, Z., Schaefer, J., 1988. Rhizocarpon RAM. em. TH. FR. subgen. *Rhizocarpon* in Europe. Taylor Fr. 20, 292–298. https://doi.org/10.1080/00040851.1988.12002677

Printzen, C., TØnsberg, T., 1999. The lichen genus *Biatora* in northwestern North America. Bryologist 102, 692–713. https://doi.org/10.2307/3244256

Prokopiev, I.A., Poryadina, L.N., Konoreva, L., Chesnokov, S., Shavarda, A.L., 2018. Variation in the Composition of Secondary Metabolites in *Flavocetraria* Lichens from Western Siberia. Russ. J. Ecol. 49, 401–405. https://doi.org/10.1134/S1067413618050107

Purvis, O.W., James, P.W., 1993. Studies on the lichens of the Azores. Part 1- Caldeira do Faial. Arquipélago. Life Mar. Sci. 11A, 1–15.

Rangaswami, S., Rao, V.S., 1954. Chemical investigation of Indian lichens. XVIII. *Parmelia nimandairana*. J. Sci. Ind. Res. 13, 403–405.

Rao, P., Shripathy, V., 1976. Chemical constituents of *Umbilicaria indica* Frey and *Ramalina farinacea* (L.) Ach. Curr. Sci. 45, 517–518.

Rao, P.S., Sarma, K.G., Seshadri, T.R., 1967. Chemical Investigation of Indian Lichens .27. Chemical Components of Some High Altitude Lichens from Western Himalayas. Indian J. Chem. 5, 0–177.

Ren, M.R., Wang, X.Y., Koh, Y.J., Hur, J.-S., 2012. Taxonomic study of the lichen genus Lobaria in South Korea. Mycobiology 40, 1–7. https://doi.org/10.5941/MYCO.2012.40.1.001

Ren, Q., 2017. A revision of the lichen genus *Ochrolechia* in China. Lichenologist 49, 67–84. https://doi.org/10.1017/S0024282916000529

Renner, B., 1982. The presence or absence of secondary metabolites in cephalodia and their possible implications. J. Hattori Bot. Lab. 52, 367–377.

Renner, B., Henssen, A., Gerstner, E., 1982. The Phytochemistry of South American *Nephroma*-Species. Zeitschrift fur Naturforsch. - Sect. C J. Biosci. 37, 739–747. https://doi.org/10.1515/znc-1982-0902

Samsudin, M.W., Said, I.M., Din, L.B., Yusoff, I., Elix, J.A., 1998. Chemotaxonomic studies of lichen from Sayap-Kinabalu, Sabah: Constituents of *Pseudocyphellaria*, *Lobaria* and *Peltigera*. ASEAN Rev. Biodivers. Environ. Conserv. 1–6.

Schiefelbein, U., Dolnik, C., Bruyn, U. de, Schultz, M., Thiemann, R., Stordeur, R., Van Den Boom, P.P.G., Litterski, B., Sipman, H.J.M., 2014. Interesting Records of Lichenized, Lichenicolous and Saprophytic Fungi from Northern Germany. Herzogia 27, 237–256. https://doi.org/10.13158/heia.27.2.2014.237

Schmitt, I., Kautz, S., Lumbsch, H.T., 2008. 6-MSAS-like polyketide synthase genes occur in lichenized ascomycetes. Mycol. Res. 112, 289–296. https://doi.org/10.1016/j.mycres.2007.08.023

Seavey, F., Seavey, J., 2015. Three new *Stirtonia* from Everglades National Park with a key to neotropical species. Lichenologist 47, 1–7. https://doi.org/10.1017/S0024282914000462

Seavey, F., Seavey, J., 2014. New additions to the lichen genus *Enterographa* (Roccellaceae) from Everglades National Park including an updated world key. Lichenologist 46, 83–93. https://doi.org/10.1017/S0024282913000662

Seavey, F., Seavey, J., Hernández M., J.E., Lücking, R., 2014. Three new *Opegrapha* species (Roccellaceae, Arthoniales) and several additions to the North American lichen mycota from Everglades National Park. Bryologist 117, 62–71. https://doi.org/10.1639/0007-2745-117.1.062

Serusiaux, E., Coppins, B.J., 2009. *Micarea sipmanii*, a new species with arbuscular pycnidia from the West Indies. Bibl. Lichenol. 99, 367–372.

Sérusiaux, E., Coppins, B.J., Diederich, P., Scheidegger, C., 2001. *Fellhanera gyrophorica*, a new European species with conspicuous pycnidia. Lichenologist 33, 285–289. https://doi.org/10.1006/lich.2001.0328

Sérusiaux, E., Goffinet, B., 2009. Taxonomy, phylogeny and biogeography of the the lichen genus *Peltigera* in Papua New Guinea. Fungal Divers. 38, 185–224.

Sipman, H.J.M., 2010. A Conspectus of the Lichens (Lichenized Fungi) of Singapore. Gard. Bull. Singapore. 61, 437–482.

Sipman, H.J.M., Raus, T., 2015. Lichens and Lichenicolous Fungi from the Island of Chios (Aegean Sea, Greece). Herzogia 28, 496–519. https://doi.org/10.13158/heia.28.2.2015.496

Sipman, H.J.M., Raus, T., 2002. An inventory of the lichen flora of Kalimnos and parts of Kos (Dodecanisos, Greece). Willdenowia 32, 351–392. https://doi.org/10.3372/wi.32.32216

Śliwa, L., Flakus, A., 2011. *Lecanora microloba*, a new saxicolous species from Poland. Lichenologist 43, 1–6. https://doi.org/10.1017/S0024282910000551

Solberg, Y., 1975. Studies on the Chemistry of Lichens, IVX Chemical Investigation of the Lichen Species *Anaptychia fusca*, *Peltigera canina*, and *Omphalodiscus spodochrous*. Zeitschrift fur Naturforsch. - Sect. C J. Biosci. 30, 445–450. https://doi.org/10.1515/znc-1975-7-805

Sparrius, L.B., Björk, C.R., 2008. *Enterographa oregonensis* (Roccellaceae), a new foliicolous species from the northwest coast of North America. Bryologist 111, 487–489. https://doi.org/10.1639/0007-2745(2008)111[487:EORANF]2.0.CO;2

Sparrius, L.B., Saipunkaew, W., 2005. *Cryptothecia punctosorediata*, a new species from Northern Thailand. Lichenologist 37, 507–509. https://doi.org/10.1017/S0024282905015495

Spielmann, A.A., Marcelli, M.P., 2008. *Punctelia* (Parmeliaceae , lichenized Ascomycota) from roadsides and slopes in the Serra Geral of Rio Grande do Sul, Brazil. Biociências 16, 79–91.

Stenroos, S., Stocker-Wörgötter, E., Yoshimura, I., Myllys, L., Thell, A., Hyvönen, J., 2003. Culture experiments and DNA sequence data confirm the identity of *Lobaria* photomorphs. Can. J. Bot. 81, 232–247. https://doi.org/10.1139/b03-027

Stepanchikova, I.S., Kukwa, M., Kuznetsova, E.S., Motiejūnaitė, J., Himelbrant, D.E., 2010. New records of lichens and allied fungi from the Leningrad Region, Russia. Folia Cryptogam. Est. 47, 77–84.

Stocker-Wörgötter, E., Elix, J.A., 2006. Morphogenetic strategies and induction of secondary metabolite biosynthesis in cultured lichen-forming Ascomycota, as exemplified by *Cladia retipora* (Labill.) Nyl. and Dactylina arctica (Richards)

Nyl. Symbiosis 41, 9–20.

Timdal, E., 2002. Three squamulose species of *Rimularia* (Lecanorales). Bryologist 105, 219–224. https://doi.org/10.1639/0007-2745(2002)105[0219:TSSORL]2.0.CO;2

Timdal, E., 1984. *Acarospora intricata* and *A. wahlenbergii* (Acarosporaceae) in Scandinavia. Nord. J. Bot. 4, 541–543. https://doi.org/10.1111/j.1756-1051.1984.tb02060.x

Timdal, E., Bendiksby, M., Kahraman, A.M., Halici, M.G., 2017. *Psora taurensis* (Psoraceae, Lecanorales), a new lichen species from Turkey. MycoKeys 21, 1–12. https://doi.org/10.3897/mycokeys.21.11726

Timdal, E., Obermayer, W., Bendiksby, M., 2016. *Psora altotibetica* (Psoraceae, Lecanorales), a new lichen species from the Tibetan part of the Himalayas. MycoKeys 13, 35–48. https://doi.org/10.3897/mycokeys.13.8824

Tønsberg, T., 2002. Additions to the lichen flora of North America XI. Bryologist 105, 122–125. https://doi.org/10.1639/0007-2745(2002)105[0122:ATTLFO]2.0.CO;2

Tønsberg, T., 1997. Additions to the Lichen Flora of North America. VI. Bryologist 100, 522. https://doi.org/10.2307/3244417

Truong, C., Clerc, P., 2003. The *Parmelia borreri* group (lichenized Ascomycetes) in Switzerland. Bot. Helv. 113, 49–61.

Tsurykau, A., Golubkov, V., Bely, P., 2018. The lichen genus *Xanthoparmelia* (Parmeliaceae) in Belarus. Folia Cryptogam. Est. 55, 125–132. https://doi.org/10.12697/fce.2018.55.13

Upreti, D.K., Joshi, Y., Bajpai, R., 2010. New records of lichen growing on monuments of Central India. Mycotaxon 38, 37–40.

Upreti, D.K., Joshi, Y., Divakar, P.K., Lumbsch, H.T., Nayaka, S., 2008. Notes on some interesting lichens from Western Ghats in India. Phytotaxonomy 8, 113–116.

Urbanavichene, I., Urbanavichus, G., 2019. New records of lichens and allied fungi from the Kostroma region, Russia. Folia Cryptogam. Est. 56, 53–62. https://doi.org/10.12697/fce.2019.56.06

Urbanavichus, G., Urbanavichene, I., 2018. New records of lichens and allied fungi from lapponia petsamoënsis, Murmansk Region, Russia. Folia Cryptogam. Est. 55, 1–6. https://doi.org/10.12697/FCE.2018.55.01

Urbanavichus, G., Vondrák, J., Urbanavichene, I., Palice, Z., Malíček, J., 2020. Lichens and allied non-lichenized fungi of virgin forests in the Caucasus State Nature Biosphere Reserve (Western Caucasus, Russia). Herzogia 33, 90. https://doi.org/10.13158/heia.33.1.2020.90

Van Den Boom, P.P.G., 2004. *Micarea subcinerea*, an additional species of the lichen flora from Western Europe. Österreichische Mykol. Gesellschaft 13, 2–5.

van den Boom, P.P.G., Brand, A.M., Coppins, B.J., 2018. A New *Micarea* Species from Western Europe, Belonging in the *Micarea denigrata* Group. Herzogia 31, 385. https://doi.org/10.13158/heia.31.1.2018.385

Van Den Boom, P.P.G., Ertz, D., 2014. A new species of *Micarea* (Pilocarpaceae) from Madeira growing on *Usnea*. Lichenologist 46, 295–301. https://doi.org/10.1017/S0024282913000698

Velmala, S., Myllys, L., Goward, T., Holien, H., Halonen, P., 2014. Taxonomy of *Bryoria* Section *Implexae* (Parmeliaceae, Lecanoromycetes) in North America and Europe, Based on Chemical, Morphological and Molecular Data. Ann. Bot. Fenn. 51, 345–371. https://doi.org/10.5735/085.051.0601

Vinet, C., Quilhot, W., Garbarino, J.A., 1990. Studies on Chilean Lichens, XIV. 2'-O-Methylhiascic Acid, a New Tridepside in *Catillaria corymbosa*, Journal of Natural Products.

Vitikainen, O., 2006. Peltigera tartarea, a new species from arctic America. J. Hattori Bot. Lab. 853–854.

Weber, W.A., 1971. A New Species of *Aspicilia* (Lichenes, Lecanoraceae) from Western United States. Bryologist 74, 183. https://doi.org/10.2307/3241835

Wei, X.L., Wang, X.Y., Koh, Y.J., Hur, J.-S., 2009. Taxonomic Study of *Peltigera* (Peltigeraceae, Ascomycota) in Korea . Mycobiology 37, 189. https://doi.org/10.4489/myco.2009.37.3.189

Wilkins, A.L., James, P.W., 1979. The chemistry of *Pseudocyphellaria impressa* s. lat. in new zealand. Lichenol. 11, 271–281. https://doi.org/10.1017/S0024282979000323

Yakovchenko, L., Davydov, E.A., Paukov, A., Frisch, A., Galanina, I., Han, J.E., Hee Moon, K., Kashiwadani, H., 2019. New Lichen Records from Korea – I. Mostly Arctic-Alpine and Tropical Species. Herzogia 31, 965. https://doi.org/10.13158/heia.31.2.2018.965

Yakovchenko, L., Davydov, E.A., Paukov, A., Konoreva, L., Chesnokov, S., Ohmura, Y., 2021. New records of arctic-alpine lichens from the Russian Far East. Herzogia 33, 455–472. https://doi.org/10.13158/heia.33.2.2020.455

Yoshimura, I., Kinoshita, Y., Yamamoto, Y., Huneck, S., Yamada, Y., 1994. Analysis of secondary metabolites from Lichen by high performance liquid chromatography with a photodiode array detector. Phytochem. Anal. 5, 197–205. https://doi.org/10.1002/pca.2800050405

Youn, U.J., So, J.E., Kim, J.H., Han, S.J., Park, H., Kim, I.C., Yim, J.H., 2018. Chemical constituents from the Antarctic lichen, *Stereocaulon caespitosum*. Biochem. Syst. Ecol. 80, 73–75. https://doi.org/10.1016/j.bse.2018.07.004

Zhang, H.J., Guo, H.F., Lou, H.X., 2006. Secondary metabolites from the Chinese lichen *Sticta nylanderiana* A. Z. Biochem. Syst. Ecol. 34, 760–762. https://doi.org/10.1016/j.bse.2006.05.007

Zhang, H.J., Sun, L.Y., Chen, C.H., Lou, H.X., 2008. Study on phenolic constituents from lichen *Lobaria yunnanensis* yoshim. Chinese Pharm. J. 43, 489.

Zhang, L., Ren, Q., 2016. Additional new species of the lichen genus *Pertusaria* from China. Telopea 19, 201–205. https://doi.org/10.7751/telopea10438

Zhang, Y.Y., Wang, X.Y., Li, L.J., Søchting, U., Yin, A.C., Wang, S.Q., Wang, L.-S., 2019. *Upretia squamulosa*, a new lichen species from the arid valley of Jinsha-Jiang river, China. Phytotaxa 402, 288–294. https://doi.org/10.11646/phytotaxa.402.6.3

Zhao, Z.T., Ren, Q., Aptroot, A., 2004. An annotated key to the lichen genus *Pertusaria* in China. Bryologist 107, 531–541. https://doi.org/10.1639/0007-2745(2004)107[531:AAKTTL]2.0.CO;2

Zhao, Z.T., Zhao, X., Gao, W., Zhou, G.L., Zhang, L.L., 2014. *Pertusaria yunnana*, a new species from south-west China. Lichenologist 46, 169–173. https://doi.org/10.1017/S0024282913000881
